# Supplementary material for: Bayesian modelling of high-throughput sequencing assays with malacoda
Source: PLoS Comput Biol. 2020 Jul 21;16(7):e1007504. doi: 10.1371/journal.pcbi.1007504 (PMC7394446; doi:10.1371/journal.pcbi.1007504)
Supplement: S1 Appendix — (PDF) [file pcbi.1007504.s001.pdf]

Model description, fitting, and diagnostics

Contents

1 Detailed model overview **1**
1.1 Data block . . . 3
1.2 Parameters block . . . 4
1.3 Model block . . . 5
1.4 Generated quantities block . . . 6

2 Model fitting walkthrough **7**
2.1 Prior MLE . . . 7
2.2 Variational first pass . . . 8
2.3 MCMC sampling . . . 9
2.4 Diagnostics . . . 10
2.5 Results visualization . . . 14

3 Session Info **15**

This document was rendered at 2020-04-19 16:58:47

This supplement provides an overview of the statistical details underlying malacoda. The code referenced here can be found at the package's Github repository, with R code in the R/ directory and Stan model files in the src/stan_files/ directory. The HTML version of this appendix features a button at the top right to Show/Hide all code blocks.

1 Detailed model overview

This section overviews the probability model underlying `malacoda`. The code block below gives a complete copy of `bc_mpra_model.stan`, the Stan file used in the package to analyze the MPRA data for a single variant given a previously estimated empirical prior (the prior estimation process is overviewed in the "Model fitting walkthrough" section below). Stan model files are broken into "blocks":

- `data` describes the numeric data to be passed to the model
- `parameters` describes the model parameters to be evaluated when fitting the model
- `model` describes the probabilistic relationships between the data and parameters
- `generated quantities` computes downstream quantities of interest that are directly computable from a given set of parameter values

The following subsections gives a detailed description and walkthrough of each block in the malacoda Stan model, which here is presented in full:

```
data {
  int<lower=0> n_rna_samples;
  int<lower=0> n_dna_samples;
  int<lower=1> n_ref; // number of reference barcodes
  int<lower=1> n_alt; // number of alternate barcodes
  int<lower=0> ref_dna_counts[n_ref, n_dna_samples];
  int<lower=0> alt_dna_counts[n_alt, n_dna_samples];
```

```
  int<lower=0> ref_rna_counts[n_ref, n_rna_samples];
  int<lower=0> alt_rna_counts[n_alt, n_rna_samples];

  real<lower=0> rna_depths[n_rna_samples];
  real<lower=0> dna_depths[n_dna_samples];

  real<lower=0> dna_m_a;
  real<lower=0> dna_m_b;

  real<lower=0> dna_p_a;
  real<lower=0> dna_p_b;

  real<lower=0> rna_m_a[2]; // 1 = ref, 2 = alt
  real<lower=0> rna_m_b[2];
  real<lower=0> rna_p_a[2];
  real<lower=0> rna_p_b[2];
}
parameters {
  vector<lower=0>[n_ref] dna_m_ref;
  vector<lower=0>[n_alt] dna_m_alt;
  real<lower=0> dna_p;

  vector<lower=0>[2] rna_m; // rna mean
  vector<lower=0>[2] rna_p; // rna phi aka size
}
model {
  // DNA priors
  for (bc in 1:n_ref) {
    dna_m_ref[bc] ~ gamma(dna_m_a, dna_m_b);
  }

  for (bc in 1:n_alt) {
    dna_m_alt[bc] ~ gamma(dna_m_a, dna_m_b);
  }

  dna_p ~ gamma(dna_p_a, dna_p_b);

  // DNA likelihood
  for (s in 1:n_dna_samples) {
    for (bc in 1:n_ref) {
      ref_dna_counts[bc,s] ~ neg_binomial_2(dna_m_ref[bc] * dna_depths[s], dna_p);
    }

    for (bc in 1:n_alt) {
      alt_dna_counts[bc,s] ~ neg_binomial_2(dna_m_alt[bc] * dna_depths[s], dna_p);
    }
  }

  // RNA priors
  for (allele in 1:2) {
    rna_m[allele] ~ gamma(rna_m_a[allele], rna_m_b[allele]);
    rna_p[allele] ~ gamma(rna_p_a[allele], rna_p_b[allele]);
  }
```

```
  // RNA likelihood
  for (s in 1:n_rna_samples) {
    for (bc in 1:n_ref) {
      ref_rna_counts[bc, s] ~ neg_binomial_2(rna_m[1] * rna_depths[s] * dna_m_ref[bc], rna_p[1]);
    }

    for (bc in 1:n_alt) {
      alt_rna_counts[bc, s] ~ neg_binomial_2(rna_m[2] * rna_depths[s] * dna_m_alt[bc], rna_p[2]);
    }
  }

}
generated quantities {
  real ref_act;
  real alt_act;
  real transcription_shift;
  ref_act = log(rna_m[1]);
  alt_act = log(rna_m[2]);
  transcription_shift = alt_act - ref_act;
}
```

In statistical notation, this model is as follows:

$$\mu_{DNA_{bc}} \sim Gamma(\alpha_{\mu_{DNA}}, \beta_{\mu_{DNA}})$$
$$\mu_{RNA} \sim Gamma(\alpha_{\mu_{RNA}}, \beta_{\mu_{RNA}})$$
$$\phi_{DNA} \sim Gamma(\alpha_{\phi_{DNA}}, \beta_{\phi_{DNA}})$$
$$\phi_{RNA} \sim Gamma(\alpha_{\phi_{RNA}}, \beta_{\phi_{RNA}})$$
$$Counts_{DNA_{s,bc}} \sim NegBin(mean = depth_s \times \mu_{DNA_{bc}}, dispersion = \phi_{DNA})$$
$$Counts_{RNA_{s,bc}} \sim NegBin(mean = depth_s \times \mu_{DNA_{bc}} \times \mu_{RNA}, dispersion = \phi_{RNA})$$

with s varying from 1 to the total number of sequencing samples of each type and bc varying from 1 to the total number of barcodes. μ_{RNA} and ϕ_{RNA} each have two elements for the ref and alt alleles.

The α and β parameters are estimated empirically, while the $depth_s$ factors are directly proportional to the number of barcode reads in each sequencing sample.

1.1 Data block

The data block describes the data that will be passed in to the model.

```
data {
  int<lower=0> n_rna_samples;
  int<lower=0> n_dna_samples;

  int<lower=1> n_ref; // number of reference barcodes
  int<lower=1> n_alt; // number of alternate barcodes

  int<lower=0> ref_dna_counts[n_ref, n_dna_samples];
  int<lower=0> alt_dna_counts[n_alt, n_dna_samples];
```

```
  int<lower=0> ref_rna_counts[n_ref, n_rna_samples];
  int<lower=0> alt_rna_counts[n_alt, n_rna_samples];

  real<lower=0> rna_depths[n_rna_samples];
  real<lower=0> dna_depths[n_dna_samples];

  real<lower=0> dna_m_a; // ref and alt DNA use the same prior, hence length = 1
  real<lower=0> dna_m_b;
  real<lower=0> dna_p_a;
  real<lower=0> dna_p_b;

  real<lower=0> rna_m_a[2]; // 1 = ref, 2 = alt
  real<lower=0> rna_m_b[2];
  real<lower=0> rna_p_a[2];
  real<lower=0> rna_p_b[2];
}
```

The data block takes in:

- the number of DNA and RNA sequencing samples
- the number of reference and alternate barcodes in the variant
- matrices of reference and alternate barcode counts in the DNA samples, with barcodes as rows and sequencing samples as columns
- matrices of reference and alternate barcode counts in the RNA samples
- the sequencing depths of the DNA and RNA samples
- the gamma parameters characterizing the empirical prior, each according to a name with three parts separated by underscores:
  - the first part refers to whether it gives the prior information for the DNA or RNA
  - the second part refers to whether it gives the prior for the mean (`m`) or dispersion (`p`)
  - the third part refers to whether it gives the alpha (`a`) or beta (`b`) parameter of the gamma prior.

In the case of the RNA prior parameters, they are given as two element vectors for the reference and alternate alleles, respectively. For example, `rna_m_a` is passed in as a two element vector, with the first element giving the alpha parameter of the gamma prior for the mean of the reference RNA counts.

1.2 Parameters block

The parameters block describes the statistical parameters whose distributions we are sampling (or fitting with ADVI) in our model:

```
parameters {
  vector<lower=0>[n_ref] dna_m_ref;
  vector<lower=0>[n_alt] dna_m_alt;
  real<lower=0> dna_p;

  vector<lower=0>[2] rna_m;
  vector<lower=0>[2] rna_p;
}
```

These parameters include:

- `dna_m_ref` and `dna_m_alt` - vectors of parameters for the DNA concentrations of the barcodes for reference and alternate alleles. These DNA concentrations are independent of sequencing depth; the model block introduces by-sample depth adjustment as a separate multiplicative factor. Notably the length of these two vectors is equal to the number of barcodes for each allele.

- `dna_p` - a single parameter that gives the dispersion of DNA barcode counts, irrespective of allele.
- `rna_m` - a length-two vector relating to the the RNA counts negative binomial distribution. `rna_m` is the multiplicative factor describing the effect of each allele on the mean of the RNA counts (similarly before adjustment by depth and DNA concentration – see the model block section).
- `rna_p` - a length-two vector relating to the the RNA counts negative binomial distribution. This gives the by-allele dispersion of RNA counts.

1.3 Model block

The model block describes the probabilistic relationships between the data and parameters.

The model block used by malacoda is fairly long, so in this subsection it is broken down further into grouped components of probabilistic statements. Each component of the model is described in the text just above where it appears.

The first two sampling statements say that the DNA mean parameters come from their input gamma priors. Notably the DNA concentration parameters come from the same prior for both the reference and alternate alleles:

```
model {

  for (bc in 1:n_ref) {
    dna_m_ref[bc] ~ gamma(dna_m_a, dna_m_b);
  }

  for (bc in 1:n_alt) {
    dna_m_alt[bc] ~ gamma(dna_m_a, dna_m_b);
  }

  ...

}
```

The next line states that the DNA dispersion parameter comes from its input gamma prior:

```
model {
  ...

  dna_p ~ gamma(dna_p_a, dna_p_b);

  ...
}
```

The next model component loops over the DNA samples and barcodes, stating that the count for barcode `bc` in sequencing sample `s` comes from a negative binomial distribution with a mean equal to the depth of sample `s` times the DNA concentration parameter for `bc` and dispersion `dna_p`.

```
model {
  ...

  for (s in 1:n_dna_samples) {
    for (bc in 1:n_ref) {
      ref_dna_counts[bc,s] ~ neg_binomial_2(dna_m_ref[bc] * dna_depths[s], dna_p);
    }

```

```
    for (bc in 1:n_alt) {
      alt_dna_counts[bc,s] ~ neg_binomial_2(dna_m_alt[bc] * dna_depths[s], dna_p);
    }
  }

  ...
}
```

The next model component describes the prior on the RNA parameters: for each allele, the prior on the mean and dispersion come from appropriately indexed gamma priors.

```
model {
  ...

  for (allele in 1:2) { // 1 = ref, 2 = alt
    rna_m[allele] ~ gamma(rna_m_a[allele], rna_m_b[allele]);
    rna_p[allele] ~ gamma(rna_p_a[allele], rna_p_b[allele]);
  }

  ...
}
```

The final lines of the model block describes the probability model for the RNA barcode counts themselves. The RNA barcode counts come from a negative binomial distribution. The mean of this binomial is equal to barcodes DNA concentration parameter (`dna_m_ref/alt[bc]`) times the sequencing sample's depth (`rna_depths[s]`) times the RNA mean parameter (`rna_m`) for the appropriate allele. Because the model includes separate terms for the depth and barcode DNA concentration in the mean of each negative binomial, the elements of `rna_m` represent the underlying transcriptional activity of the reference and alternate alleles that the assay is intended to infer. There is also a dispersion parameter for the RNA counts of each allele.

```
model {
  ...

  for (s in 1:n_rna_samples) {
    for (bc in 1:n_ref) {
      ref_rna_counts[bc, s] ~ neg_binomial_2(rna_m[1] * rna_depths[s] * dna_m_ref[bc], rna_p[1]);
    }

    for (bc in 1:n_alt) {
      alt_rna_counts[bc, s] ~ neg_binomial_2(rna_m[2] * rna_depths[s] * dna_m_alt[bc], rna_p[2]);
    }
  }
}
```

1.4 Generated quantities block

The generated quantities block of the model transforms and aggregates several parameters into quantities that are more easy to interpret. This is done for computational convenience and does not influence the model fitting process.

The model block below defines three quantities that are characteristic of the variant. `ref_act` is the reference allele's activity – the log-mean of the RNA counts after accounting for depth and DNA input concentration. `alt_act` provides a similar quantity for the alternate allele. `transcription_shift` is the difference between `alt_act` and `ref_act`. A negative transcription shift means that the alternate allele has lower transcriptional activity than the reference allele.

```
generated quantities {
  real ref_act;
  real alt_act;
  real transcription_shift;
  ref_act = log(rna_m[1]);
  alt_act = log(rna_m[2]);
  transcription_shift = alt_act - ref_act;
}
```

2 Model fitting walkthrough

This section provides a detailed overview of the model fitting process used by `malacoda`. It walks through each step called inside of malacoda's main fitting function `malacoda::fit_mpra_model()` and how it makes use of the probability model defined in the previous section. It also provides an overview of model diagnostics that can be used to check the fit's convergence and the method for visually assessing the model fit.

2.1 Prior MLE

After checking that all inputs are valid, `fit_mpra_model()` fits an empirical prior. By default it uses a marginal prior, however informative annotations or groupings can be provided with the `annotations` or `group_df` arguments. The function calls `fit_marg_prior()`, `fit_cond_prior()`, or `fit_grouped_prior()` prior as needed with the given inputs. All three of these functions take the input data table, fit per-variant maximum likelihood estimates, then return a prior object of the appropriate form (marginal, conditional, or grouped). Each prior estimation function also takes care to discard poorly represented barcodes according to the representation cutoff specified by the user.

The maximum likelihood estimation is performed using `rstan::optimizing()` using a simplified version of the malacoda model above without the gamma priors and a generic initialization. `rstan::optimizing()` uses the L-BFGS algorithm to maximize the log-density of this likelihood-only model.

Once maximum likelihood estimates have been obtained for each variant, `rstan::optimizing()` is used again to fit a gamma distribution acoss all variants for each parameter type (means and dispersions for DNA and ref/alt RNA). This gives the α and β parameters that define the empirical prior.

In the case of dispersion parameters, we discard the top 5% largest estimates before estimating the gamma priors. This is done to counteract the bias in the maximum likelihood estimation of disperion parameters (which is demonstrated by simulation in section 3 of the negative binomial variance estimation supplement). The choice of 5% is motivated by practicality. More principled methods of removing bias from dispersion parameter estimates exist, however because they typically have no closed form solution and require numerical optimization. Because this would need to be done for thousands of dispersion parameter estimates, this would drastically increase the computation time for prior estimation. Because the 5% largest dispersion estimates come from the variants with the lowest variance (recall that $V = \mu + \frac{\mu^2}{\phi}$), this amounts to slightly increasing the regularizing effect of the gamma prior that we estimate.

More informative priors can be fit with `fit_grouped_prior()` or `fit_cond_prior()`. These functions use user-supplied information to weight the prior-estimation process. `fit_grouped_prior()` is used when the variants in the assay fall into discrete categories, fitting a marginal prior within each group. `fit_cond_prior()` is used when the variant annotations are continuous. For each variant, all other variants in the assay are weighted according to how close they are to the variant in question in annotation space. The weights are taken from a t distribution which is centered at the variant in question and scaled so that pre-set number of additional variants provide non-negligible weight. These weights are then used to scale each variant's contribution to the gamma fit.

As an example, the table below shows the example marginal prior included in the `malacoda` package (which is derived from the Ulirsch dataset).

prior_type	prior	alpha_est	beta_est	acid_type	allele
mu_prior	gamma_fit[6]	0.98	0.22	DNA	NA
phi_prior	gamma_fit[6]	2.80	0.83	DNA	NA
mu_prior	gamma_fit[6]	1.05	1.13	RNA	Mut
mu_prior	gamma_fit[6]	1.04	1.11	RNA	Ref
phi_prior	gamma_fit[6]	1.78	1.14	RNA	Mut
phi_prior	gamma_fit[6]	1.75	1.12	RNA	Ref

Each row describes a gamma prior:

- the `prior_type` column indicates whether it's a mean or dispersion parameter
- the `prior` column is the gamma fit returned by `rstan::optimizing()`
- the `alpha_est` column are the estimates of the alpha (shape) parameters for each gamma distribution
- the `beta_est` column are the estimates of the beta (rate) parameters for each gamma distribution
- the `acid_type` column specifies whether the prior applies to DNA or RNA
- the `allele` column indicates the allele for the RNA rows

Empirical priors can also be provided to `fit_mpra_model()` directly as an input if they have been estimated separately beforehand using `fit_marg_prior()`, `fit_grouped_prior`, or `fit_cond_prior()`.

2.2 Variational first pass

After the empirical priors have been estimated, `fit_mpra_model()` moves on to fitting the core model (outlined in section 1 above) for each variant. If the `vb_pass` argument is enabled, the code initially tries a fast variational approximation for each variant to see if the variant is worth a time-consuming MCMC run.

Variational approximation is a method for evaluating a fast approximation to a posterior distribution by minimizing the Kullback-Leibler divergence between an approximating distribution and the posterior. Stan's interface for variational inference (Kucukelbir et al., Adv Neural Inf Process Syst 2017) is available through the `rstan::vb()` function. This function automatically transforms each parameter to support on the real number line (e.g. taking the log of strictly positive negative binomial mean parameters), then uses a Gaussian variational distribution and stochastic optimization to fit the approximating distribution. Because the transformed parameter distributions are usually not truly Gaussian, the posterior approximation will not perfectly match the true posterior regardless of how well the optimization converges. Nonetheless, in practice the variational approximation works well as demonstrated later in this section.

Because each of the mean and dispersion parameters estimated in a malacoda fit are all constrained to be positive real numbers, `rstan::vb()` will first apply a log transformation to each parameter (thus transforming their support to the entire real axis) before fitting the Gaussian variational distribution.

In malacoda this process is performed with the following command:

```
vb_res = rstan::vb(stanmodels$bc_mpra_model,
                   data = data_list, # a list of counts, sequencing depths, and prior parameters
                   tol_rel_obj = .0005)
```

In practice, this is roughly five to twenty times faster than drawing 2000 MCMC posterior samples with `rstan::sampling()` while producing estimates that are accurate when compared to the MCMC estimates. The plot below compares the variational estimates of RNA mean parameters and transcription shift to the MCMC estimates (using long MCMC chains with 50,000 samples).

```r
knitr::include_graphics(path = '/home/ghazi/dev_malacoda/outputs/ec_plot.png')
```

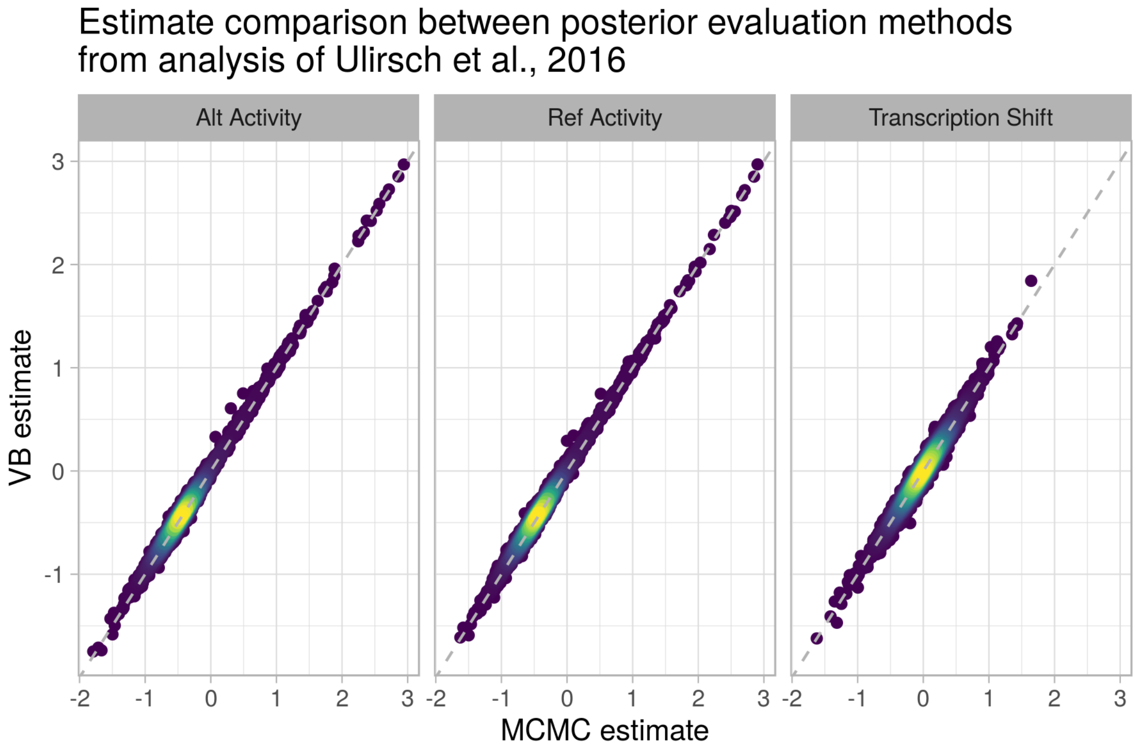

The variational fit is used to decide if the variant is worth the slower but asymptotically exact MCMC run. If an 80% posterior interval on transcription shift excludes zero as a credible value, the variant is passed on to the MCMC sampler, otherwise the variational result is passed to the global results table with a note that VB was used for posterior evaluation. The 80% threshold is intentionally easy to overcome; it is desirable to strike a balance between speed and precise, unbiased estimates for important variants. The 80% threshold can be adjusted with the `vb_prob` argument to `fit_mpra_model()`.

2.3 MCMC sampling

If the variational first pass deems a variant worthy of MCMC, the data is passed to `rstan::sampling()`. This is the function that calls Stan's No-U-Turn-Sampler (Gelman et al., JMLR 2014).

NUTS is an MCMC algorithm that improves upon Hamiltonian Monte Carlo (HMC) by checking for a high-dimensional generalization of a U turn at each leapfrog step within each iteration. When the trajectory starts to turn around, the Hamiltonian evolution is stopped and the next proposal is sampled with bias from the simulated path in parameter space. This prevents the computationally wasteful oscillatory behavior that can occur in a poorly tuned HMC system. Like other MCMC systems and unlike variational approximations, NUTS is asymptotically exact. The approximation error in posterior estimates can be reduced arbitrarily close to 0 by taking enough posterior samples.

The code block below gives the command that `fit_mpra_model` uses:

```r
sampler_res = rstan::sampling(stanmodels$bc_mpra_model,
                              data = data_list, # a list of counts, sequencing depths, and prior parame
                              chains = n_chains,
                              warmup = n_warmup,
                              iter = n_per_chain,
                              cores = 1,
```

```
                        verbose = verbose,
                        refresh = refresh_setting)
```

By default, malacoda uses 200 warmup samples (`n_warmup`) and enough samples per chain (`n_per_chain`) to achieve 2000 total post-warmup samples. This works quite well in practice for exploratory use (as demonstrated in the Diagnostics section below), though we recommend to users setting the `tot_samp` argument to `fit_mpra_model` to 50,000 or as high as the user's computational resources allow for publication quality analyses.

The `sampling()` command above otherwise uses `rstan`'s default arguments, which in practice perform well. These include (among many others):

- `adapt_delta`, the goal acceptance rate: 0.8
- `max_treedepth`, the log2 maximum number of post-warmup leapfrog steps: 20

The full details on how Stan works can be found at the Stan website.

If the `adaptive_precision` argument to `fit_mpra_model` is set to `TRUE`, then the function that calls the sampler will check if either edge of the posterior interval used to make a binary call of functionality is near zero. It does this by checking if the posterior meets two conditions: a slightly wider interval includes zero and a slightly narrower interval excludes zero. "Slightly" in this situation is defined as +/- half of the remaining probability mass, so a 95% interval cutoff would be checked with a 97.5% interval and a 92.5% interval. If the check meets the two conditions, the sampler length is doubled in order to increase the precision on the functionality-defining interval.

The variant's posterior is saved into the user-specified output directory into an RData file named according to the variant's ID in the data passed to `fit_mpra_model`.

2.4 Diagnostics

In practice Stan is able to fit the malacoda model easily and efficiently, but it is important to confirm this by checking the appropriate diagnostic outputs.

Because malacoda produces `stanfit` objects, the outputs can be used with any of the packages in the Stan ecosystem for posterior analysis. Additional details and references on the meaning and interpretation of these diagnostics can be found in the posterior analysis chapter of the Stan reference manual.

The examples below shows the diagnostic outputs for variant `1_155271258_1-3`, one of the top hits in the Ulirsch dataset. This variant's fit is used as a demonstrative example throughout the remainder of this document. The model fit in question used 4 excessively long chains with a total of 50,000 MCMC samples.

2.4.1 N_{eff} and $\hat{R}$

The "effective sample size" N_{eff} of a posterior simulation indicates the approximate number of posterior samples after discounting for auto-correlation within the MCMC chains, while the "potential scale reduction" statistic $\hat{R}$ compares the variance of parameter samples within and between chains. N_{eff} should be comparable to the number of posterior samples and as high as possible, while $\hat{R}$ should ideally be 1. Note that N_{eff} may sometimes exceed the total number of posterior samples if the samples tend to be anticorrelated; this is not problematic behavior. Stan provides warning messages when these diagnostics indicate imperfect convergence. More detail on these metrics can be found in the MCMC Sampling and Posterior Analyiss chapters of the online Stan reference manual.

Simply printing the `sampler_res` object output for each malacoda variant will display the N_{eff} and $\hat{R}$ diagnostics for each parameter in the posterior fit. The table below shows the values for these diagnostics for the fit of the example variant above.

```r
library(rstan)
summary(sampler_res)$summary %>%
  .[,c(9,10)] %>%
  knitr::kable(digits = 5)
```

	n_eff	Rhat
dna_m_ref[1]	54892.20	1.00000
dna_m_ref[2]	59087.69	1.00003
dna_m_ref[3]	59828.30	0.99994
dna_m_ref[4]	58328.04	1.00000
dna_m_ref[5]	56856.56	0.99995
dna_m_ref[6]	63303.59	1.00003
dna_m_ref[7]	57510.60	0.99996
dna_m_ref[8]	60798.48	0.99997
dna_m_ref[9]	58917.84	0.99999
dna_m_ref[10]	54581.71	0.99998
dna_m_ref[11]	60054.47	1.00007
dna_m_ref[12]	55686.28	0.99995
dna_m_ref[13]	61373.80	0.99996
dna_m_ref[14]	56355.86	0.99997
dna_m_alt[1]	64273.10	1.00001
dna_m_alt[2]	62992.34	0.99997
dna_m_alt[3]	63196.57	0.99996
dna_m_alt[4]	60980.55	0.99996
dna_m_alt[5]	52880.50	0.99999
dna_m_alt[6]	61945.70	0.99996
dna_m_alt[7]	61905.98	0.99997
dna_m_alt[8]	61738.42	0.99999
dna_m_alt[9]	56933.61	0.99998
dna_m_alt[10]	64188.33	0.99998
dna_m_alt[11]	60022.77	0.99995
dna_m_alt[12]	59351.22	1.00000
dna_m_alt[13]	64429.24	0.99998
dna_m_alt[14]	61266.41	1.00000
dna_p	52155.93	0.99993
rna_m[1]	31672.02	1.00000
rna_m[2]	39978.66	0.99997
rna_p[1]	62737.18	0.99995
rna_p[2]	64169.51	0.99999
ref_act	31554.81	1.00001
alt_act	40209.54	0.99997
transcription_shift	36321.55	0.99998
lp__	19389.34	1.00009

2.4.2 MCMC traces

MCMC traces shows the values of the parameter draws as a function of the iteration number in the MCMC chain. They ideally show rapid convergence from the initialization values, followed by full exploration of the set of reasonable values. There should not be any long-term drift in the chain which would imply the chain(s) have not yet converged. Separate MCMC chains should be well mixed.

MCMC traces can be displayed using the `mcmc_trace()` function in the `bayesplot` package. The example

below shows that the posterior on the variant's reference activity level seems to have converged well before exiting the warmup period used in this example (shaded area):

```
post_draws = rstan::extract(sampler_res,
                            inc_warmup = TRUE,
                            permuted = FALSE)

bayesplot::mcmc_trace(post_draws[1:4000,,],
                      pars = 'ref_act', # pars = c('alt_act', 'transcription_shift'),
                      n_warmup = 2000) +
  labs(x = 'MCMC iteration') +
  scale_color_viridis_d(end = .92)
```

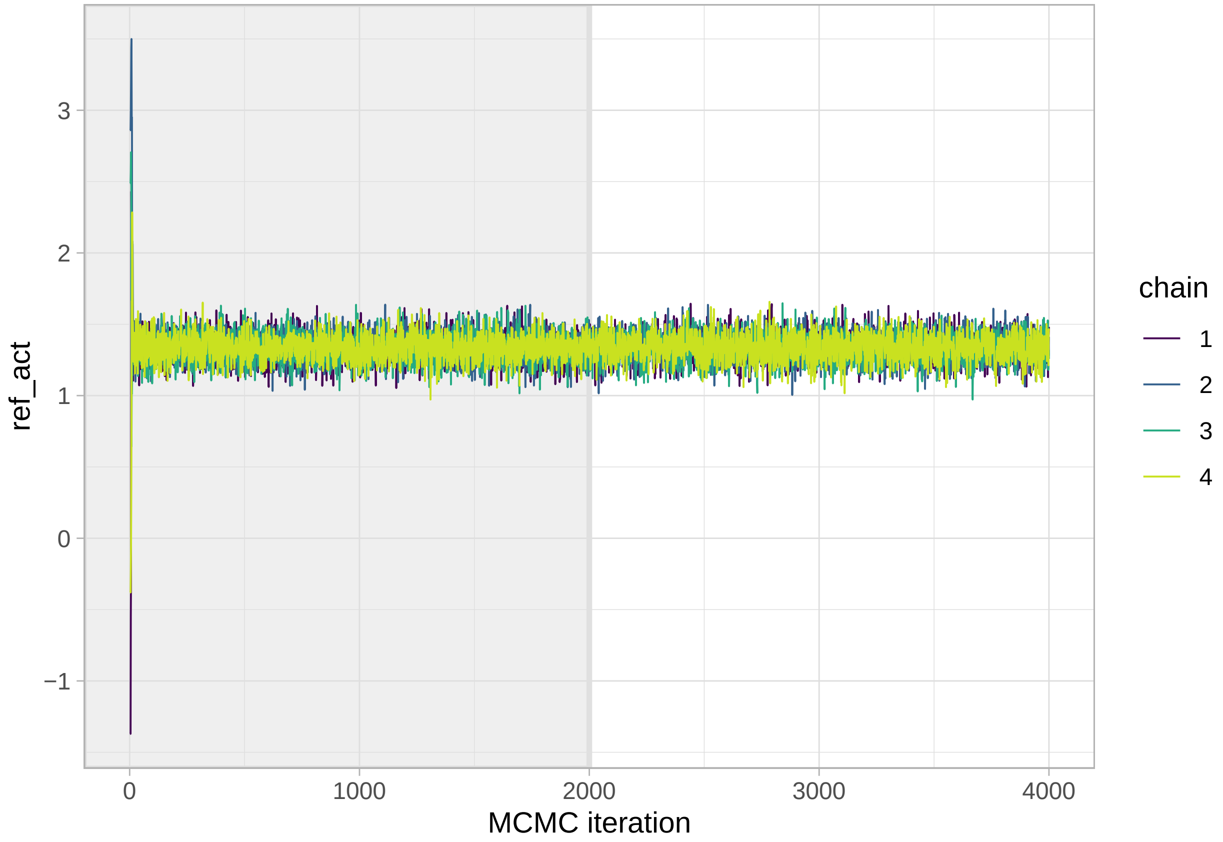

Other model parameters can be examined through the `pars` argument.

Zooming in to the first 100 samples, the samples of transcription shift seem to have adapted from the initialization within 25 samples.

```
bayesplot::mcmc_trace(post_draws[1:100,,], pars = 'transcription_shift') +
  labs(x = 'MCMC iteration') +
  scale_color_viridis_d(end = .92)
```

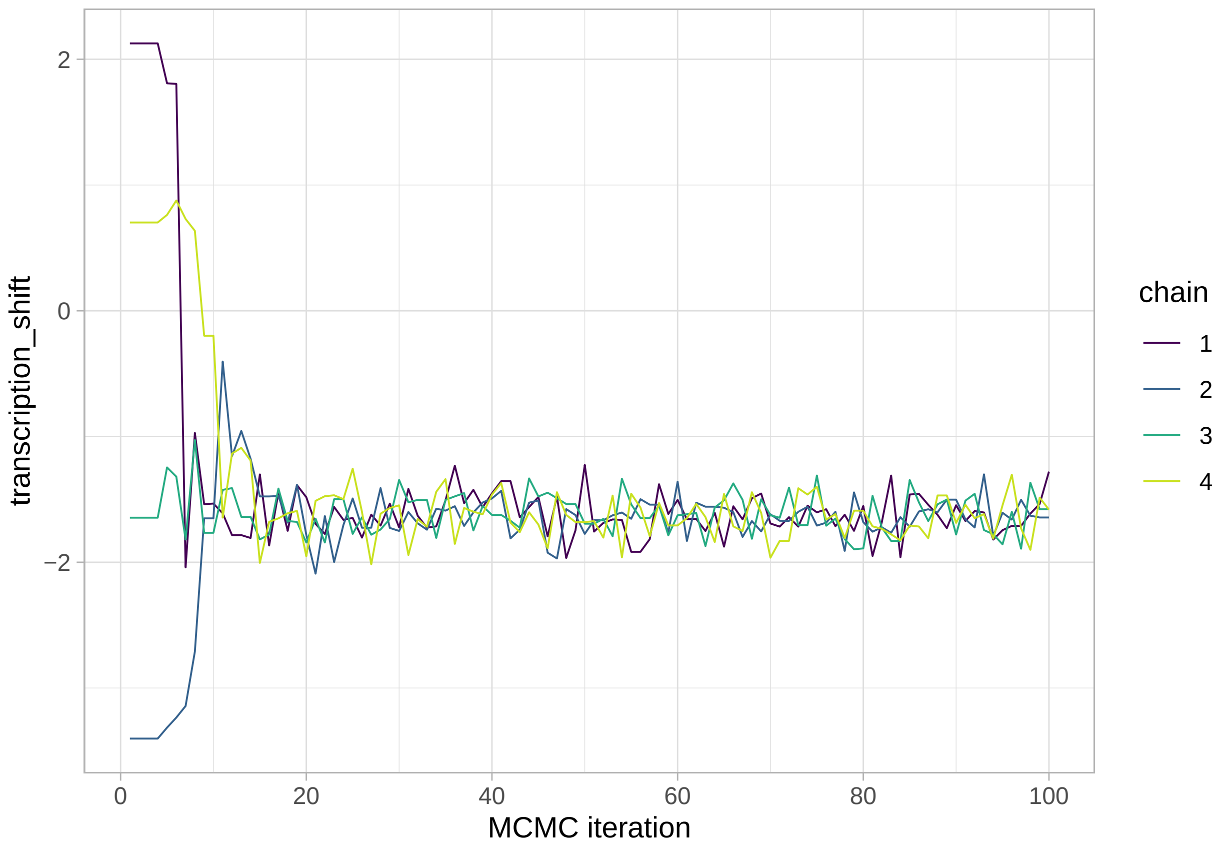

2.4.3 NUTS Energy diagnostic

The NUTS energy diagnostic compares the distribution of the transition energies $\pi_{\Delta E}$ and the marginal energy distribution π_E. When these distributions are close together (as they are in this case), it implies that the sampler is efficiently exploring the tails of the posterior. This can be used to double-check the sampler efficiency characteristics for important variants. This plot shows the NUTS Energy diagnostic plot for the model fit of our example variant:

```
sr_np = bayesplot::nuts_params(sampler_res)

mcmc_nuts_energy(sr_np) +
  labs(title = 'NUTS Energy diagnostic by chain')
```

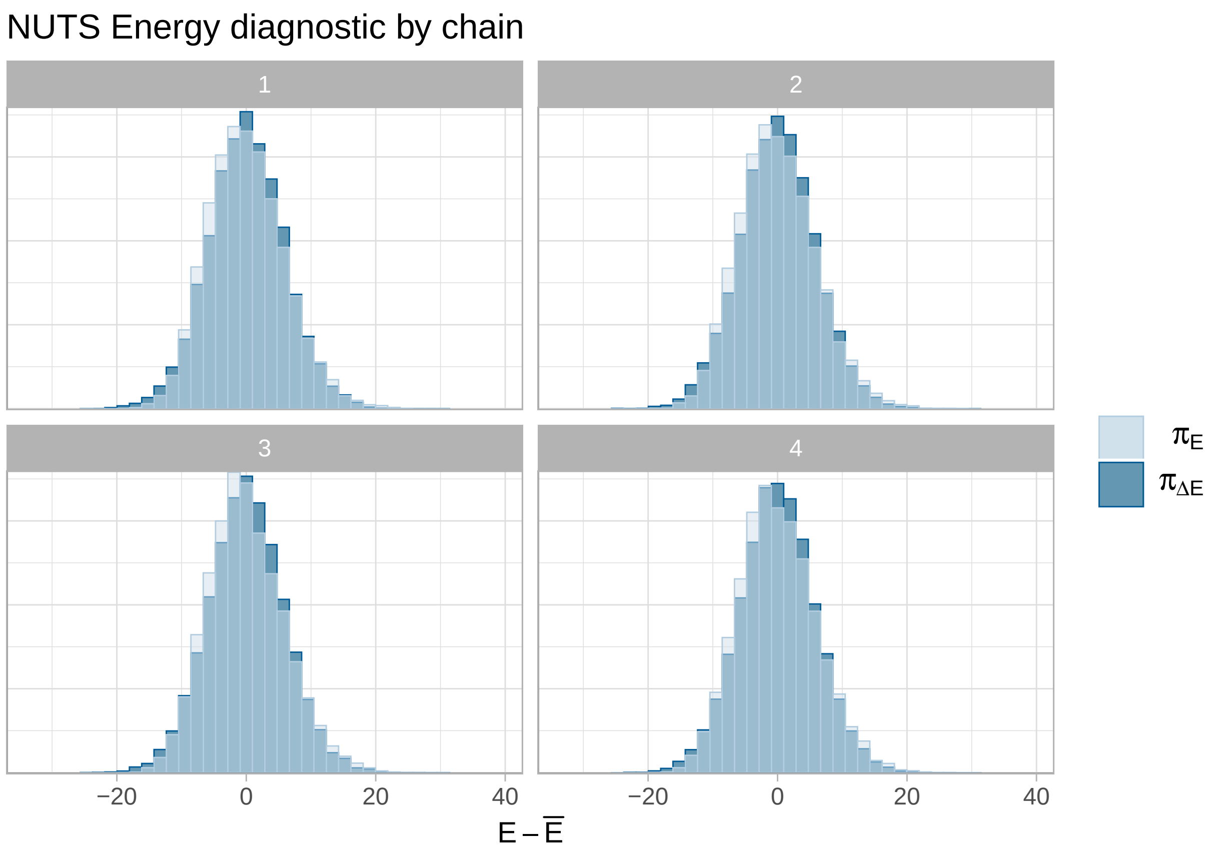

Again the functionality to perform this visualization comes from the `mcmc_nuts_energy()` function in the `bayesplot` package. More diagnostic visualization methods can be found on the bayesplot website

2.5 Results visualization

A given variant's posterior can be visualized by passing the sampler output to the function `malacoda_intervals`. This displays a simple posterior interval plot for each parameter in the model, broken down by the various types of parameters. The outer black bars default to 95% of the posterior mass, while the inner blue bars capture 80%. A dotted line at 0 can be used to assess at a glance whether 0 is a credible value for a given parameter.

```
malacoda::malacoda_intervals(sampler_res)
```

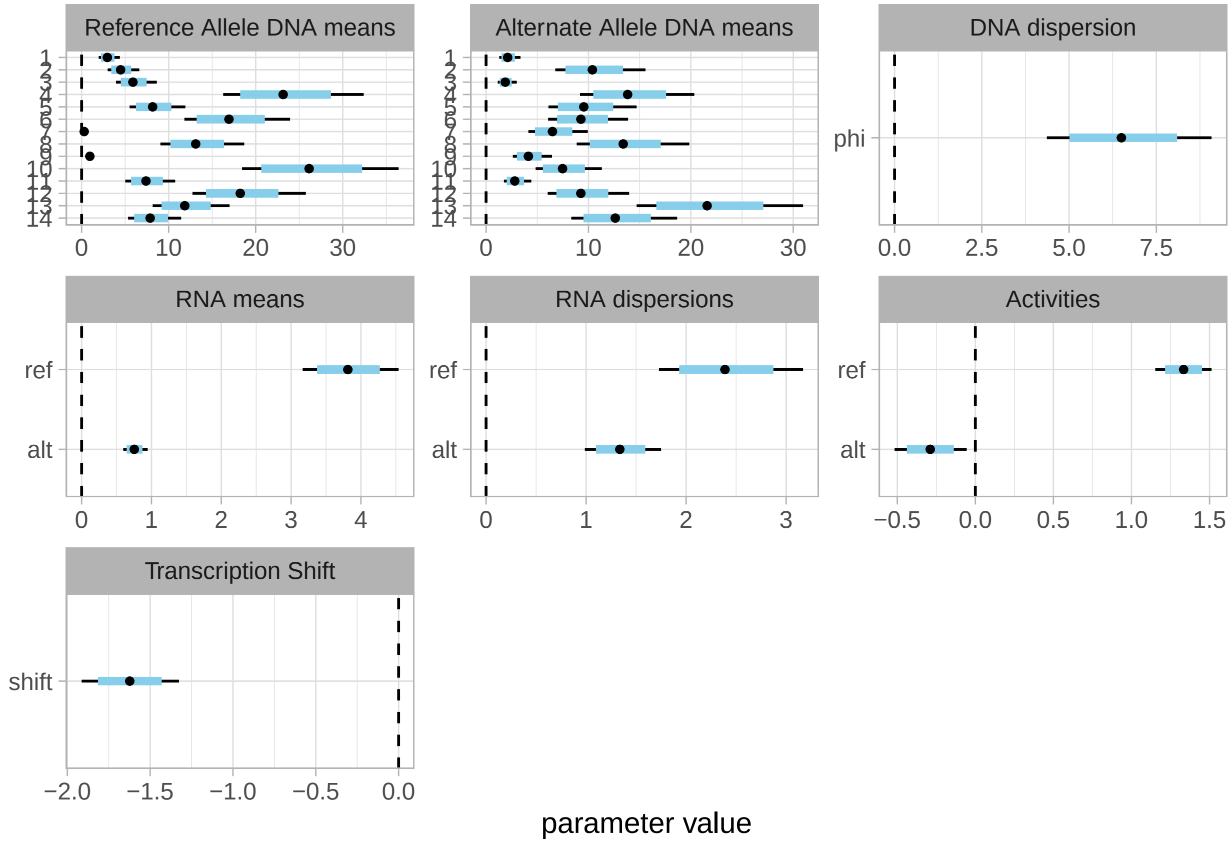

3 Session Info

```
devtools::session_info()
```

```
## - Session info ---------------------------------------------------------------
##  setting  value
##  version  R version 3.6.2 (2019-12-12)
##  os       Linux Mint 19.1
##  system   x86_64, linux-gnu
##  ui       X11
##  language en_US
##  collate  en_US.UTF-8
##  ctype    en_US.UTF-8
##  tz       America/Chicago
##  date     2020-02-20
##
## - Packages -------------------------------------------------------------------
##  package     * version date       lib source
##  assertthat    0.2.1   2019-03-21 [1] CRAN (R 3.6.0)
##  backports     1.1.5   2019-10-02 [1] CRAN (R 3.6.1)
##  bayesplot   * 1.7.1   2019-12-01 [1] CRAN (R 3.6.2)
##  broom         0.5.4   2020-01-27 [1] CRAN (R 3.6.2)
##  callr         3.4.1   2020-01-24 [1] CRAN (R 3.6.2)
##  cellranger    1.1.0   2016-07-27 [1] CRAN (R 3.6.0)
```

```
##  cli           2.0.1   2020-01-08 [1] CRAN (R 3.6.2)
##  codetools     0.2-16  2018-12-24 [4] CRAN (R 3.5.2)
##  colorspace    1.4-1   2019-03-18 [1] CRAN (R 3.6.0)
##  crayon        1.3.4   2017-09-16 [1] CRAN (R 3.6.0)
##  DBI           1.1.0   2019-12-15 [1] CRAN (R 3.6.2)
##  dbplyr        1.4.2   2019-06-17 [1] CRAN (R 3.6.1)
##  desc          1.2.0   2018-05-01 [1] CRAN (R 3.6.0)
##  devtools      2.2.1   2019-09-24 [1] CRAN (R 3.6.2)
##  digest        0.6.23  2019-11-23 [1] CRAN (R 3.6.2)
##  dplyr       * 0.8.4   2020-01-31 [1] CRAN (R 3.6.2)
##  ellipsis      0.3.0   2019-09-20 [1] CRAN (R 3.6.1)
##  evaluate      0.14    2019-05-28 [1] CRAN (R 3.6.0)
##  fansi         0.4.1   2020-01-08 [1] CRAN (R 3.6.2)
##  farver        2.0.3   2020-01-16 [1] CRAN (R 3.6.2)
##  forcats     * 0.4.0   2019-02-17 [1] CRAN (R 3.6.0)
##  fs            1.3.1   2019-05-06 [1] CRAN (R 3.6.0)
##  generics      0.0.2   2018-11-29 [1] CRAN (R 3.6.0)
##  ggplot2     * 3.2.1   2019-08-10 [1] CRAN (R 3.6.1)
##  ggridges      0.5.2   2020-01-12 [1] CRAN (R 3.6.2)
##  glue          1.3.1   2019-03-12 [1] CRAN (R 3.6.0)
##  gridExtra     2.3     2017-09-09 [1] CRAN (R 3.6.0)
##  gtable        0.3.0   2019-03-25 [1] CRAN (R 3.6.0)
##  haven         2.2.0   2019-11-08 [1] CRAN (R 3.6.2)
##  highr         0.8     2019-03-20 [1] CRAN (R 3.6.0)
##  hms           0.5.3   2020-01-08 [1] CRAN (R 3.6.2)
##  htmltools     0.4.0   2019-10-04 [1] CRAN (R 3.6.2)
##  httr          1.4.1   2019-08-05 [1] CRAN (R 3.6.2)
##  inline        0.3.15  2018-05-18 [1] CRAN (R 3.6.0)
##  jsonlite      1.6.1   2020-02-02 [1] CRAN (R 3.6.2)
##  knitr         1.27    2020-01-16 [1] CRAN (R 3.6.2)
##  labeling      0.3     2014-08-23 [1] CRAN (R 3.6.0)
##  lattice       0.20-38 2018-11-04 [4] CRAN (R 3.5.1)
##  lazyeval      0.2.2   2019-03-15 [1] CRAN (R 3.6.0)
##  lifecycle     0.1.0   2019-08-01 [1] CRAN (R 3.6.1)
##  loo           2.2.0   2019-12-19 [1] CRAN (R 3.6.2)
##  lubridate     1.7.4   2018-04-11 [1] CRAN (R 3.6.0)
##  magrittr    * 1.5     2014-11-22 [1] CRAN (R 3.6.0)
##  malacoda      0.2.2   2020-02-03 [1] local
##  matrixStats   0.55.0  2019-09-07 [1] CRAN (R 3.6.2)
##  memoise       1.1.0   2017-04-21 [1] CRAN (R 3.6.0)
##  modelr        0.1.5   2019-08-08 [1] CRAN (R 3.6.2)
##  munsell       0.5.0   2018-06-12 [1] CRAN (R 3.6.0)
##  nlme          3.1-144 2020-02-06 [4] CRAN (R 3.6.2)
##  pillar        1.4.3   2019-12-20 [1] CRAN (R 3.6.2)
##  pkgbuild      1.0.6   2019-10-09 [1] CRAN (R 3.6.2)
##  pkgconfig     2.0.3   2019-09-22 [1] CRAN (R 3.6.1)
##  pkgload       1.0.2   2018-10-29 [1] CRAN (R 3.6.0)
##  plyr          1.8.5   2019-12-10 [1] CRAN (R 3.6.2)
##  prettyunits   1.1.1   2020-01-24 [1] CRAN (R 3.6.2)
##  processx      3.4.1   2019-07-18 [1] CRAN (R 3.6.1)
##  ps            1.3.0   2018-12-21 [1] CRAN (R 3.6.0)
##  purrr       * 0.3.3   2019-10-18 [1] CRAN (R 3.6.1)
##  R6            2.4.1   2019-11-12 [1] CRAN (R 3.6.2)
##  Rcpp          1.0.3   2019-11-08 [1] CRAN (R 3.6.2)
```

```
##  readr       * 1.3.1   2018-12-21 [1] CRAN (R 3.6.0)
##  readxl        1.3.1   2019-03-13 [1] CRAN (R 3.6.0)
##  remotes       2.1.0   2019-06-24 [1] CRAN (R 3.6.1)
##  reprex        0.3.0   2019-05-16 [1] CRAN (R 3.6.0)
##  reshape2      1.4.3   2017-12-11 [1] CRAN (R 3.6.0)
##  rlang         0.4.4   2020-01-28 [1] CRAN (R 3.6.2)
##  rmarkdown     2.1     2020-01-20 [1] CRAN (R 3.6.2)
##  rprojroot     1.3-2   2018-01-03 [1] CRAN (R 3.6.0)
##  rstan       * 2.19.2  2019-07-09 [1] CRAN (R 3.6.1)
##  rstantools    2.0.0   2019-09-15 [1] CRAN (R 3.6.2)
##  rstudioapi    0.10    2019-03-19 [1] CRAN (R 3.6.0)
##  rvest         0.3.5   2019-11-08 [1] CRAN (R 3.6.2)
##  scales        1.1.0   2019-11-18 [1] CRAN (R 3.6.2)
##  sessioninfo   1.1.1   2018-11-05 [1] CRAN (R 3.6.0)
##  StanHeaders * 2.19.0  2019-09-07 [1] CRAN (R 3.6.2)
##  stringi       1.4.5   2020-01-11 [1] CRAN (R 3.6.2)
##  stringr     * 1.4.0   2019-02-10 [1] CRAN (R 3.6.0)
##  testthat      2.3.1   2019-12-01 [1] CRAN (R 3.6.2)
##  tibble      * 2.1.3   2019-06-06 [1] CRAN (R 3.6.0)
##  tidyr       * 1.0.2   2020-01-24 [1] CRAN (R 3.6.2)
##  tidyselect    1.0.0   2020-01-27 [1] CRAN (R 3.6.2)
##  tidyverse   * 1.3.0   2019-11-21 [1] CRAN (R 3.6.2)
##  usethis       1.5.1   2019-07-04 [1] CRAN (R 3.6.1)
##  vctrs         0.2.2   2020-01-24 [1] CRAN (R 3.6.2)
##  viridisLite   0.3.0   2018-02-01 [1] CRAN (R 3.6.0)
##  withr         2.1.2   2018-03-15 [1] CRAN (R 3.6.0)
##  xfun          0.12    2020-01-13 [1] CRAN (R 3.6.2)
##  xml2          1.2.2   2019-08-09 [1] CRAN (R 3.6.2)
##  yaml          2.2.1   2020-02-01 [1] CRAN (R 3.6.2)
##
## [1] /home/ghazi/R/x86_64-pc-linux-gnu-library/3.6
## [2] /usr/local/lib/R/site-library
## [3] /usr/lib/R/site-library
## [4] /usr/lib/R/library
```
