# Supplementary material for: Bayesian modelling of high-throughput sequencing assays with malacoda
Source: PLoS Comput Biol. 2020 Jul 21;16(7):e1007504. doi: 10.1371/journal.pcbi.1007504 (PMC7394446; doi:10.1371/journal.pcbi.1007504)
Supplement: S2 Appendix — (PDF) [file pcbi.1007504.s002.pdf]

Negative Binomial variance estimation

Contents

1 Negative binomial parameterization **1**

2 Description of point estimates of variance parameters **1**

3 Simulating variance parameter point estimates **2**

4 NB likelihood surfaces **4**

5 Data requirements for size estimation **7**

6 Effect on mean estimation **9**

7 Conclusion **13**

8 Session Information **13**

1 Negative binomial parameterization

A negative binomial distribution is characterized by its mean μ and dispersion ϕ parameters. The density function over the non-negative integers k under this parameterization is as follows:

$$Pr(X = k) = \frac{\Gamma(\phi + k)}{k!\Gamma(\phi)} \left(\frac{\phi}{\phi + \mu}\right)^{\phi} \left(\frac{\mu}{\phi + \mu}\right)^{k}$$

The variance of this distribution is $V(X) = \mu + \frac{\mu^2}{\phi}$, so as the dispersion ϕ goes to infinity, $V(X)$ goes to the mean μ. As the dispersion goes to zero, the variance goes to infinity. We use this parameterization partly for the natural interpretability of the mean parameter and partly for consistency with the `*nbinom` family of functions in R and `neg_binomial_2()` in Stan.

2 Description of point estimates of variance parameters

Most software for non-Bayesian analysis of sequencing counts uses point estimates of a variance parameter at some point. Typically this is done by fitting a smooth curve between empirical estimates of means and variances, and taking either points near or above the curve.

For example, DESeq2 uses a two step dispersion estimation process using the $\alpha = \frac{1}{\phi}$ negative binomial parameterization. It fits a global inverse relationship between the gene-wise normalized means $\bar{\mu}$ and α estimates: $\alpha_{trend}(\bar{\mu}) = \frac{\alpha_1}{\bar{\mu}} + \alpha_0$ with α_0 and α_1 being estimated globally; the second step chooses α that is either near the curve or more than a set distance above the curve (see Figure 1 and Methods of Love et al., Genome Biology 2014). In practice, this works out to choosing an estimate for α that maximizes the estimated variance: $\hat{V_i} = max(V_{i,empirical}, V_{i,trend})$

This procedure is effective at preventing estimates that erroneously indicate data are underdispersed. However, the first term inside this max() operation can commonly be off by a large factor as is shown in the next section. In a high-throughput parallel assay, an overly large variance estimate would lower the sensitivity of the analysis. Furthermore, even small inaccuracies in dispersion parameter estimates can have a large impact on the inference on the mean parameter, as shown in section 6 "Effect on mean estimation".

3 Simulating variance parameter point estimates

The code chunk below simulates 10,000 samples of 10 draws from a negative binomial distribution with $\mu = 100$ and $\phi = 1$. We chose these parameter values as round numbers that are roughly representative of a given cell in an array of MPRA barcode counts. For comparison, the DNA counts in the Ulirsch dataset feature lower μ and ϕ as shown in the table below. In practice, lower μ and ϕ would exacerbate the issues being identified in this sction.

```
load("~/malacoda/data/umpra.RData")

empirical_phi = function(V, m){
  # V = m + m**2 / phi
  m**2 / (V - m)
}

umpra %>%
  select(matches('DNA')) %>%
  gather(sample_id, counts) %>%
  group_by(sample_id) %>%
  summarise(empirical_mean = mean(counts),
            empirical_variance = var(counts),
            approx_phi = empirical_phi(empirical_variance, empirical_mean)) %>%
  knitr::kable(digits = 3)
```

sample_id	empirical_mean	empirical_variance	approx_phi
K562_DNA1	76.488	29148.27	0.201
K562_DNA2	52.819	13796.95	0.203

Given that $Var(X) = \mu + \frac{\mu^2}{\phi}$ for the negative binomial distribution, this means that the true value of the variance of these samples is 10,100. It then fits maximum likelihood estimators to each 10-draw sample (by directly calling `optim()` on the log-likelihood) and extracts the mean, dispersion, and variance estimates from this fit. It then draws a histogram of the sampling distribution of these variance, with a solid line at the true value and dotted lines at 2/3x and 3/2x the true value.

Because the maximum likelihood estimation process must estimate the mean in order to estimate the variance, the maximum likelihood estimates of sample variance are systematically lower than the true value. Furthermore, a substantial fraction are off by a large factor. Critically, around 20% are above 3/2x the true value (right dotted line). This means that the analyses of 20% of subgroups using variance estimates chosen in the manner from the previous section will be much less statistically powerful than they should be.

```
library(tidyverse)
library(magrittr)

set.seed(1234)
get_nb_mle = function(nb_draws){
  fn_to_min = function(param_vec){
```

```
    -sum(dnbinom(nb_draws,
                 mu = param_vec[1],
                 size = param_vec[2],
                 log = TRUE))
  }

  optim(par = c(100,1),
        fn_to_min)
}

sim_df = tibble(data_samples = map(1:10000, ~rnbinom(10, mu = 100, size = 1)),
                ml_estimates = parallel::mclapply(data_samples, get_nb_mle, mc.cores = 4),
                mean_estimate = map_dbl(ml_estimates, ~.x$par[1]),
                disp_estimate = map_dbl(ml_estimates, ~.x$par[2]),
                variance_estimate = mean_estimate + mean_estimate**2 / disp_estimate)

ggplot(sim_df, aes(variance_estimate)) +
  geom_histogram(boundary = 0) +
  geom_vline(xintercept = 10100*c(2/3, 1, 3/2),
             lty = c(2,1,2), color = 'grey10') +
  scale_x_log10() +
  theme_light() +
  labs(title = 'Maximum likelihood estimates of negative binomial variances\nare highly dispersed',
      x = expression('MLE variance of 10 NB('~mu~'=100, '~phi~'=1) samples'),
      subtitle = paste0(round(100*mean(sim_df$variance_estimate > 3/2 * 10100), digits = 3), '% fall al
```

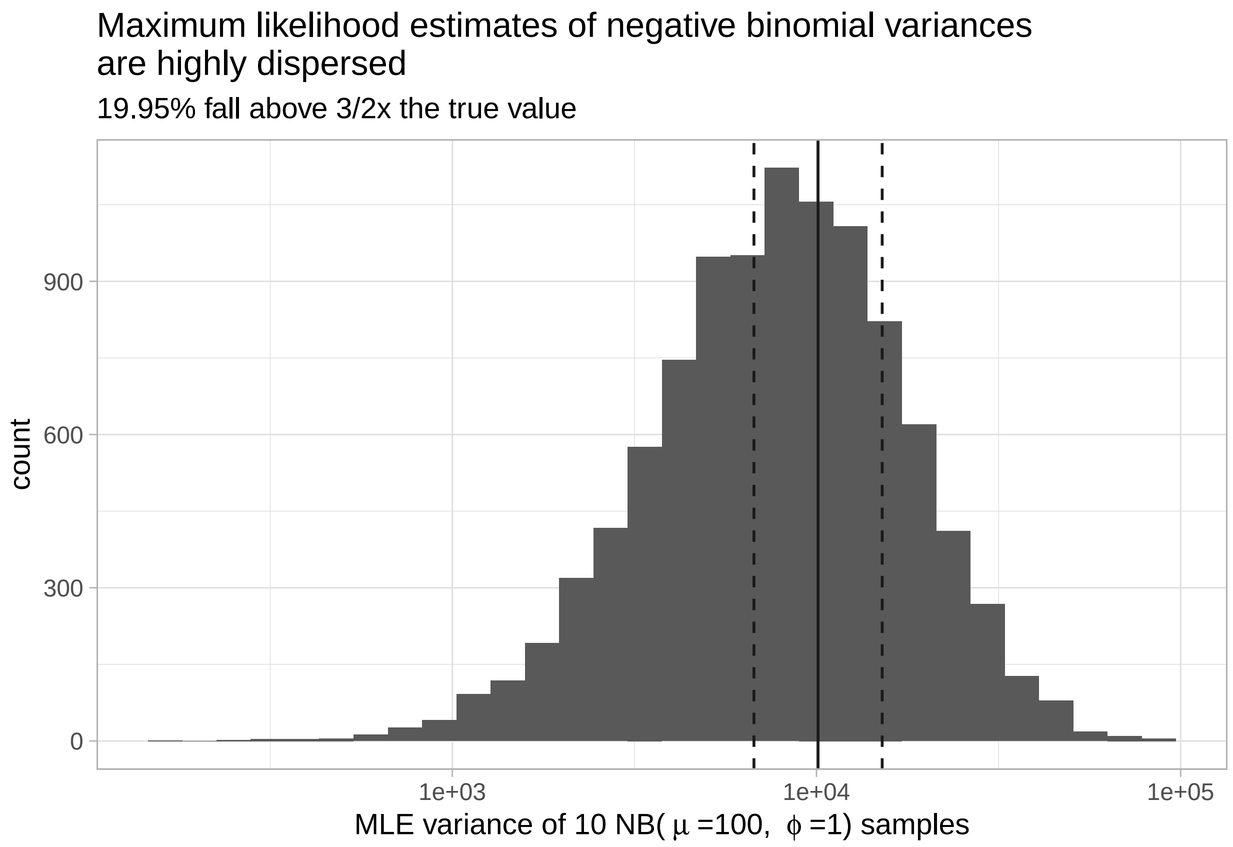

4 NB likelihood surfaces

In order to gain insight into the behavior of dispersion parameter estimates, it is useful to inspect the probability surface implied by a negative binomial model. This plot shows the likelihood surface of a call to `rnbinom(n = 10, mu = 100, size = 1)`, with a dot at the true parameter value.

```
set.seed(1234)
nb_ll = function(param_vec, nb_draws){
  sum(dnbinom(nb_draws, # no minus
              mu = param_vec[1],
              size = param_vec[2],
              log = TRUE))
}

surface_grid = expand.grid(list(mean_value = seq(10, 300, length.out = 20),
                                dispersion_value = 10**seq(-5, 4.5, length.out = 20)))

get_ll_surface = function(nb_draws){
  surface_grid %>%
    mutate(ll_value = map2_dbl(mean_value, dispersion_value,
                               ~nb_ll(c(.x, .y), nb_draws = nb_draws)),
           variance_value = mean_value + mean_value^2 / dispersion_value)
}

nb_draw_example = rnbinom(10, mu = 100, size = 1)

ll_df = surface_grid %>%
  mutate(ll_value = map2_dbl(mean_value, dispersion_value,
                             ~nb_ll(c(.x, .y), nb_draws = nb_draw_example)),
         variance_value = mean_value + mean_value^2 / dispersion_value)

true_value = tibble(mean_value = 100,
                    dispersion_value = 1)


nb_l = function(param_vec, nb_draws){
    prod(dnbinom(nb_draws, # prod, no minus
                mu = param_vec[1],
                size = param_vec[2])) # no log
}


surface_grid = expand.grid(list(mean_value = seq(10, 300, length.out = 20),
                                dispersion_value = 10**seq(-1, 1, length.out = 20)))

get_l_surface = function(nb_draws){
  surface_grid %>%
    mutate(ll_value = map2_dbl(mean_value, dispersion_value,
                               ~nb_l(c(.x, .y), nb_draws = nb_draws)),
           variance_value = mean_value + mean_value^2 / dispersion_value)
}
ll_df = surface_grid %>%
  mutate(ll_value = map2_dbl(mean_value, dispersion_value,
                             ~nb_l(c(.x, .y), nb_draws = nb_draw_example)),
```

```
         variance_value = mean_value + mean_value^2 / dispersion_value)

true_value = tibble(mean_value = 100,
                    dispersion_value = 1)

ll_df %>%
  ggplot(aes(mean_value, dispersion_value)) +
  geom_tile(aes(fill = ll_value,
                color = ll_value)) +
  scale_y_log10() +
  scale_fill_viridis_c(labels = scales::scientific_format(digits = 2)) +
  scale_color_viridis_c(guide = FALSE) +
  geom_point(data = true_value, size = 1, color = 'red') +
  theme_light() +
  labs(fill = 'likelihood',
       title = 'The likelihood surface of 10 random draws shows the majority\nof the probability mass b
       color = NULL)
```

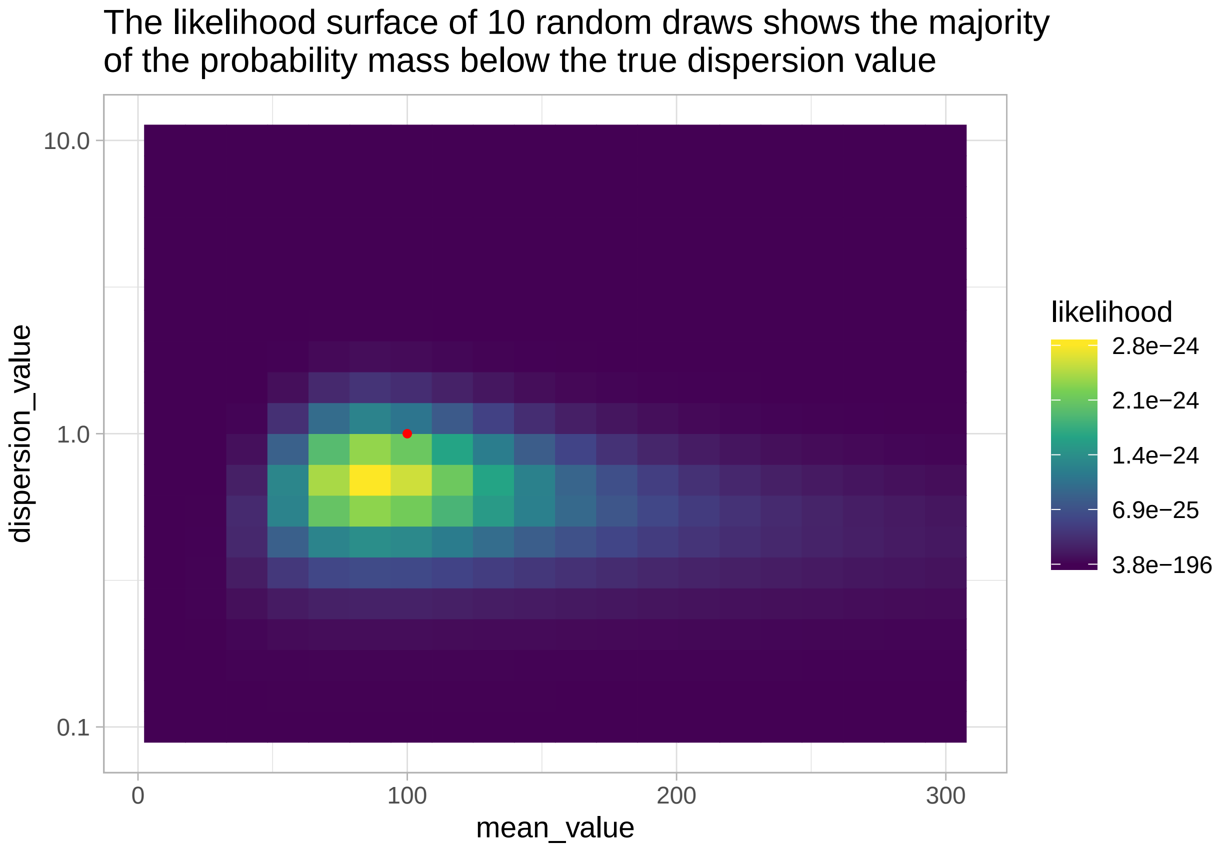

Note the log scale on the vertical axis. This shows that it's easy to occasionally allocate statistical credibility to tiny values of ϕ (meaning large variance values) for negative binomial draws. We want to avoid doing choosing small values of ϕ because it reduces the sensitivity of the analysis. It's harder to detect a difference between samples if the variance estimate is huge.

Repeating this for sixteen more example size 10 draws shows that this behavior is not rare across repeated samples.

```r
set.seed(123)
sim_df = tibble(data_samples = map(1:16, ~rnbinom(10, mu = 100, size = 1)),
                l_grids = map(data_samples,
                              get_l_surface),
                ll_grids = map(data_samples,
                               get_ll_surface),
                draw_id = 1:16) %>%
  select(-1)

make_sim_plot = function(sim_grid){
  sim_grid %>%
    ggplot(aes(mean_value, dispersion_value)) +
    geom_tile(aes(fill = ll_value,
                  color = ll_value), show.legend = FALSE) +
    scale_y_log10() + scale_fill_viridis_c() +
    scale_color_viridis_c(guide = FALSE) +
    geom_point(data = true_value, size = 2, color = 'red') +
    theme_light() +
    labs(fill = 'likelihood',
         y = NULL,
         x = NULL) +
    theme(plot.margin = margin(unit(c(1,1,1,1), 'pt')))
}

sim_df %<>%
  mutate(sim_plot = map(l_grids, make_sim_plot))

gridExtra::grid.arrange(grobs = sim_df$sim_plot)
```

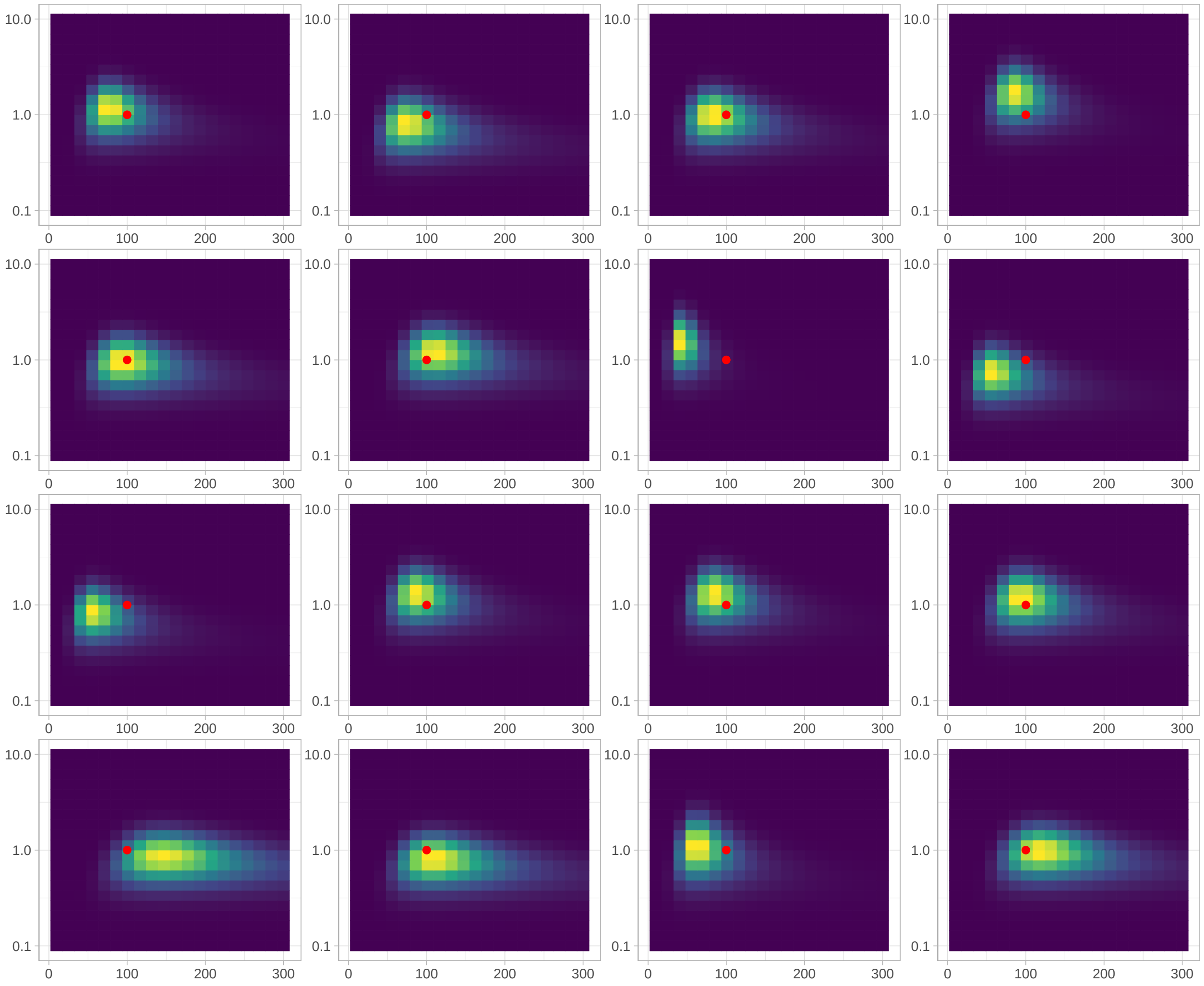

Notably, they usually ascribe non-negligible amounts of density near the true value. This means that through the use of a carefully chosen prior distribution we can regularize our dispersion parameter estimates towards the truth without a significant clash between the prior and likelihood.

5 Data requirements for size estimation

How much data is necessary to precisely characterize the size parameter? In the code chunk below we take 200 random samples from a $NegBin(\mu = 100, \phi = 1)$ 30 times. For each of the 30 example datasets, we fit a maximum likelihood estimate for the dispersion for the first k samples, ranging k from 4 to 200. This demonstrates how the maximum likelihood estimate of the dispersion evolves as more data is drawn. Each of the 30 samples is plotted as a grey line, with the average across all 30 shown in red.

```
get_nb_fit_dispersion = function(counts){
  fn_to_min = function(par_vec){
    -sum(dnbinom(x = counts,
                 mu = par_vec[1],
                 size = par_vec[2],
                 log = TRUE))
  }
```

```r
  optim(fn = fn_to_min,
        par = c(100,1),
        method = 'L-BFGS-B',
        control = list(parscale = c(10,.1)),
        lower = c(0,0))$par[2]
}

# sample NB(mu = 100, phi = 1) 200 times. For each sample, compute the
# dispersion parameter estimate up to that sample.
disp_sim = tibble(x = rnbinom(200,
                              mu = 100,
                              size = 1)) %>%
  mutate(cumulative_disp_fit = c(rep(NA, 3), map_dbl(4:200,
                                                     ~get_nb_fit_dispersion(.data$x[1:.x]))))

run_disp_sim = function(t){
  success = FALSE

  while(!success){ # the optimization fails sometimes, just retry if it does
    sim_res = try(tibble(x = rnbinom(200,
                                     mu = 100,
                                     size = 1)) %>%
                    mutate(cumulative_disp_fit = c(rep(NA, 3),
                                                   map_dbl(4:200,
                                                           ~get_nb_fit_dispersion(.data$x[1:.x])))),
                  silent = TRUE)
    if(!all(class(sim_res) == 'try-error')){
      success = TRUE
    }
  }

  return(sim_res)
}

# Run 30 simulations
multi_sim = tibble(x = 1:30,
                   y = parallel::mclapply(1:30, run_disp_sim, mc.cores = 4))

mean_line = multi_sim %>%
  unnest_legacy %>%
  mutate(iter = rep(1:200, times = 30)) %>%
  group_by(iter) %>%
  summarise(mean_disp_est = mean(cumulative_disp_fit))

multi_sim_plot = multi_sim %>%
  unnest_legacy %>%
  mutate(iter = rep(1:200, times = 30)) %>%
  ggplot(aes(iter, cumulative_disp_fit)) +
  geom_line(aes(group = x),
            alpha = .33) +
  geom_line(data = mean_line,
            aes(iter, mean_disp_est),
            color = 'red') +
```

```
  ylim(c(0,3)) +
  xlim(c(0,200)) +
  geom_hline(yintercept = 1,
             lty = 2,
             color = 'grey90') +
  geom_hline(yintercept = 1,
             lty = 3,
             color = 'black') +
  theme_light() +
  labs(x = 'sample size',
       y = 'cumulative dispersion\nestimate') +
  labs(title = 'Dispersion estimates are commonly strongly biased\nuntil well above of 50 samples')

print(multi_sim_plot)
ggsave(multi_sim_plot,
       filename = '/home/ghazi/dev_malacoda/outputs/multi_sim_plot.png')
```

```
knitr::include_graphics('/home/ghazi/dev_malacoda/outputs/multi_sim_plot.png')
```

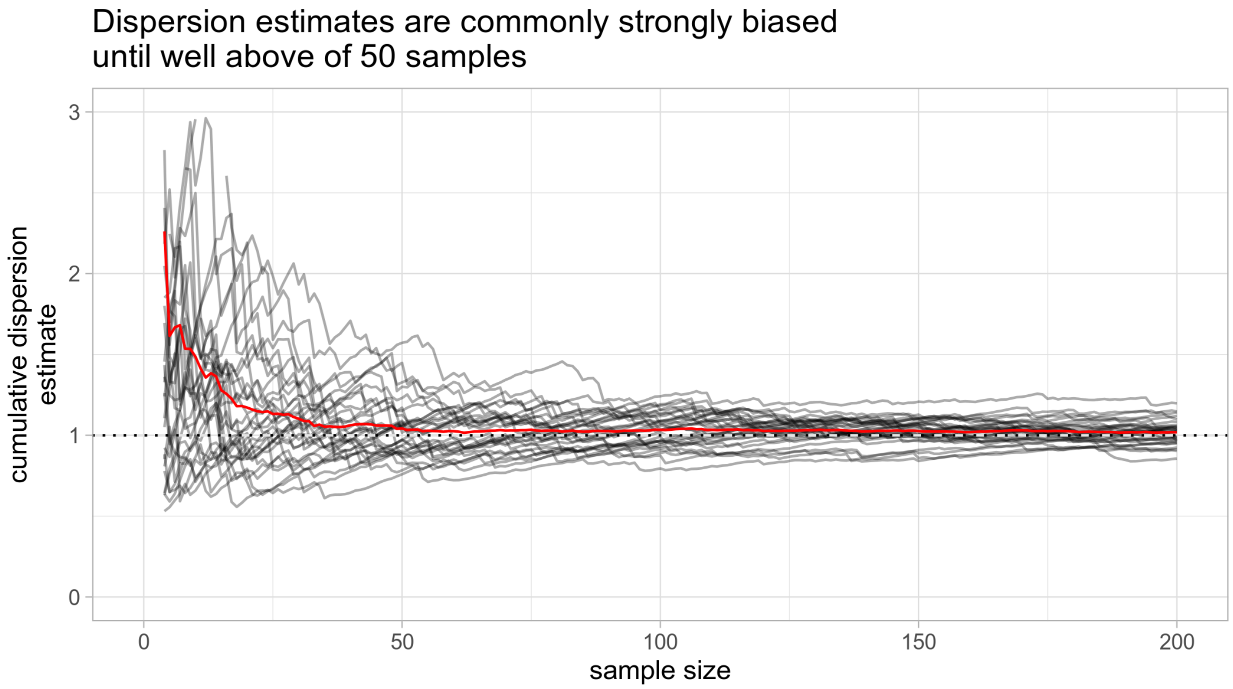

The maximum likelihood estimates tend to be too large, meaning estimates of the variance are too small. Even with simulated data, it often takes upwards of 50 data points to overcome this bias. In realistic experiments with oligonucleotide synthesis costs, sequencing costs, and complex experimental structures, it could take even more data to precisely characterize ϕ.

6 Effect on mean estimation

Why care about the accuracy of dispersion estimates?

Because the model for ϕ impacts the posterior on μ. To demonstrate this in the simple case of estimating a single negative binomial distribution, we take 30 draws from $NB(\mu = 100, \phi = 1)$ on which we'll fit two

Stan models. The first models μ and ϕ jointly (with no prior on ϕ and a relatively flat $Gamma(1, .01)$ prior on μ), and another that takes a single point estimate as a guess at ϕ as an input. We then examine the posterior on μ. When the guess at ϕ is exactly right ($\phi = 1$, green), it's comparable to the first model:

```
library(rstan)
library(tidyverse)

set.seed(1234)
obs_to_model = rnbinom(30,
                       mu = 100,
                       size = 1)

free_model = '
data{
int N;
int obs[N];
}
parameters{
real mean_val;
real dispersion;
}
model{
  // no prior on dispersion
  mean_val ~ gamma(1, .01);
  obs ~ neg_binomial_2(mean_val, dispersion);
}'

free_model_obj = stan_model(model_code = free_model)
free_result = sampling(object = free_model_obj,
                       data = list(N = length(obs_to_model),
                                   obs = obs_to_model),
                       cores = 4,
                       iter = 6000,
                       warmup = 1000,
                       control = list(adapt_delta = .95),
                        show_messages = FALSE)

free_post_plot = free_result %>%
  rstan::extract(pars = 'mean_val') %>%
  as_tibble %>%
  ggplot(aes(mean_val)) +
  geom_histogram(bins = 40)

set_model = '
data {
  int N;
  int obs[N];
  real dispersion_val;
}
parameters {
  real mean_val; // only one free parameter now
}
model {
  mean_val ~ gamma(1, .01);
```

```
  obs ~ neg_binomial_2(mean_val, dispersion_val);
}
'

set_model_obj = stan_model(model_code = set_model)

get_set_dispersion = function(dispersion_val){

  set_result = sampling(object = set_model_obj,
                        data = list(N = length(obs_to_model),
                                    obs = obs_to_model,
                                    dispersion_val = dispersion_val),
                        cores = 4,
                        iter = 6000,
                        warmup = 1000,
                        control = list(adapt_delta = .95),
                        show_messages = FALSE)
  return(set_result)
}

sim_df = tibble(dispersion_guess = c(.3, .75, 1, 1.33, 3)) %>%
  mutate(sampler_res = map(dispersion_guess, get_set_dispersion)) %>%
  mutate(mean_hdi = map(sampler_res,
                        ~quantile(rstan::extract(.x, pars = 'mean_val')$mean_val,probs = c(.025, .975))
  mutate(hdi_string = map_chr(mean_hdi, ~paste0(format(.x, digits = 3), collapse = ' - ')))

free_post = free_result %>%
  rstan::extract(pars = 'mean_val') %>%
  as_tibble %>%
  rename(mean_val_post = mean_val)

mean_sim_plot = sim_df %>%
  mutate(mean_val_post = map(sampler_res, ~rstan::extract(.x, pars = 'mean_val')[[1]])) %>%
  select(dispersion_guess, mean_val_post) %>%
  unnest_legacy %>%
  mutate(dispersion_guess = factor(round(dispersion_guess, digits = 3))) %>%
  ggplot(aes(mean_val_post)) +
  stat_density(geom = 'line', adjust = 1.5,
               mapping = aes(color = dispersion_guess),
               position = 'identity',
               lwd = 1.25) +
  stat_density(color = 'grey30',
               geom = 'line',
               position = 'identity',
               data = free_post, adjust = 1.5,
               lty = 2) +
  labs(title = 'Using a set dispersion parameter strongly impacts the posterior on the mean',
       x = 'mean',
       color = 'Input dispersion guess',
       y = 'posterior density',
       subtitle = 'Dotted line shows posterior when modelling dispersion as a free parameter') +
  theme_light() +
  annotate('text',
```

```
           x = 150,
           y = seq(.03, .017, length.out = 5),
           label = sim_df$hdi_string,
           color = RColorBrewer::brewer.pal(5, 'Set1')) +
  annotate('text',
           x = 153,
           y = .033,
           label = '95% intervals:')+
  xlim(c(25, 200)) +
  scale_color_brewer(palette = 'Set1')


ggsave(mean_sim_plot,
       filename = '/home/ghazi/dev_malacoda/outputs/mean_sim_plot.png')

print(mean_sim_plot)
```

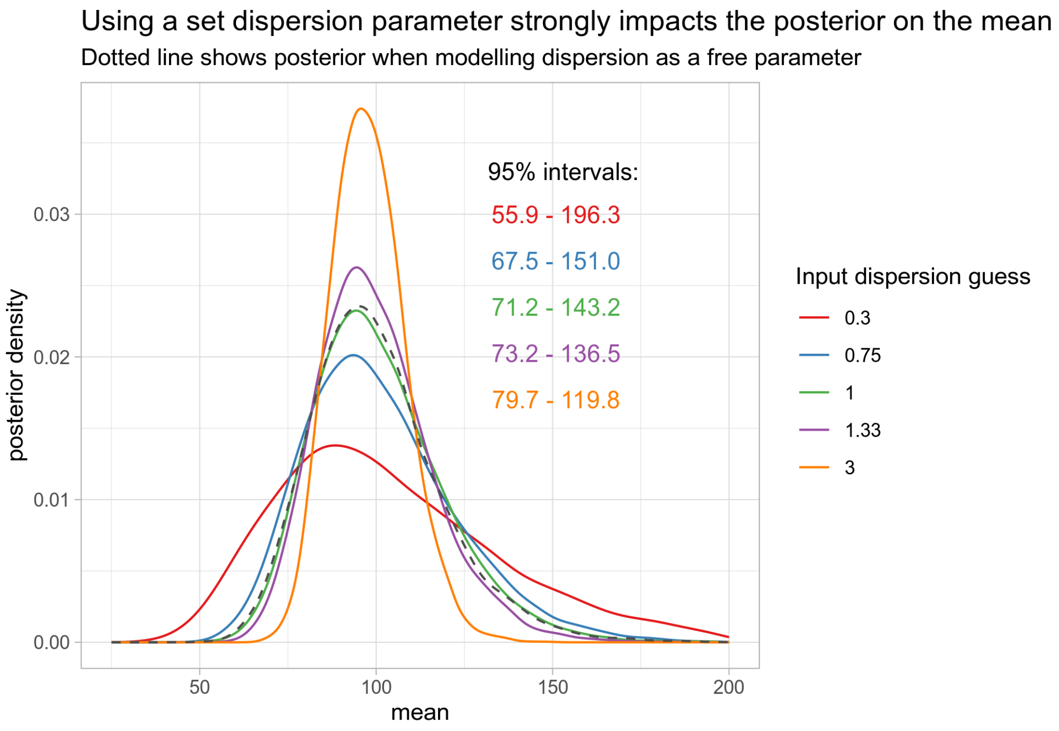

Modelling ϕ freely, even with an improper prior, recapitulates the mean posterior when the ϕ guess is exactly right (the green line).

Using incorrect guesses can greatly widen or narrow the mean posterior however, as shown by the intervals listed on the plot. These intervals are how we make downstream decisions on statistically credible values of variant activity and in turn transcription shift. A procedure that gives us dispersion guesses that are too small (red, blue) yields a posterior that is overbroad and unsure. Too large (purple, orange) and the posterior is overconfident. Both of these situations could happen using the point estimates resulting from the procedure in section 2.

Putting a prior distribution on ϕ doesn't make much of a difference in a small example like this, but it helps in larger, more structurally complex models. Importantly it imposes the desirable shrinkage effect comparable to what originally motivated the empirical smoothing procedure described in the second section.

The model used by malacoda is more complicated than estimating a single negative binomial distribution as shown in this example. Nonetheless, the value of quantifying the uncertainty on ϕ is clear. In an MPRA context, ϕ is unknown. By modelling ϕ as a unknown parameter rather than utilizing a point estimate, we obtain more accurate inference on not only ϕ but also μ and related quantities including the transcription shift. The simulation results presented in Figure 3 of the manuscript drive home the performance benefits of this approach.

7 Conclusion

Allowing the dispersion parameter to vary freely as part of the posterior simulation is one of the key benefits from malacoda. Allowing the model to allocate statistical credibility to a range of dispersion values avoids making over-confident claims about the precision of our dispersion parameter estimates and avoids the inferential downside associated with potentially inaccurate point guesses. Nonetheless, malacoda's empirical Bayesian prior on ϕ naturally regularizes dispersion estimates, avoiding the overfitting that originally motivated early work on empirical shrinkage of dispersion estimates.

8 Session Information

```r
devtools::session_info()
```

```
## - Session info ---------------------------------------------------------------
##  setting  value
##  version  R version 3.6.2 (2019-12-12)
##  os       Linux Mint 19.1
##  system   x86_64, linux-gnu
##  ui       X11
##  language en_US
##  collate  en_US.UTF-8
##  ctype    en_US.UTF-8
##  tz       America/Chicago
##  date     2020-02-19
##
## - Packages -------------------------------------------------------------------
##  package     * version date       lib source
##  assertthat    0.2.1   2019-03-21 [1] CRAN (R 3.6.0)
##  backports     1.1.5   2019-10-02 [1] CRAN (R 3.6.1)
##  broom         0.5.4   2020-01-27 [1] CRAN (R 3.6.2)
##  callr         3.4.1   2020-01-24 [1] CRAN (R 3.6.2)
##  cellranger    1.1.0   2016-07-27 [1] CRAN (R 3.6.0)
##  cli           2.0.1   2020-01-08 [1] CRAN (R 3.6.2)
##  codetools     0.2-16  2018-12-24 [4] CRAN (R 3.5.2)
##  colorspace    1.4-1   2019-03-18 [1] CRAN (R 3.6.0)
##  crayon        1.3.4   2017-09-16 [1] CRAN (R 3.6.0)
##  DBI           1.1.0   2019-12-15 [1] CRAN (R 3.6.2)
##  dbplyr        1.4.2   2019-06-17 [1] CRAN (R 3.6.1)
##  desc          1.2.0   2018-05-01 [1] CRAN (R 3.6.0)
##  devtools      2.2.1   2019-09-24 [1] CRAN (R 3.6.2)
##  digest        0.6.23  2019-11-23 [1] CRAN (R 3.6.2)
##  dplyr       * 0.8.4   2020-01-31 [1] CRAN (R 3.6.2)
##  ellipsis      0.3.0   2019-09-20 [1] CRAN (R 3.6.1)
##  evaluate      0.14    2019-05-28 [1] CRAN (R 3.6.0)
```

```
##  fansi         0.4.1   2020-01-08 [1] CRAN (R 3.6.2)
##  farver        2.0.3   2020-01-16 [1] CRAN (R 3.6.2)
##  forcats     * 0.4.0   2019-02-17 [1] CRAN (R 3.6.0)
##  fs            1.3.1   2019-05-06 [1] CRAN (R 3.6.0)
##  generics      0.0.2   2018-11-29 [1] CRAN (R 3.6.0)
##  ggplot2     * 3.2.1   2019-08-10 [1] CRAN (R 3.6.1)
##  glue          1.3.1   2019-03-12 [1] CRAN (R 3.6.0)
##  gridExtra     2.3     2017-09-09 [1] CRAN (R 3.6.0)
##  gtable        0.3.0   2019-03-25 [1] CRAN (R 3.6.0)
##  haven         2.2.0   2019-11-08 [1] CRAN (R 3.6.2)
##  highr         0.8     2019-03-20 [1] CRAN (R 3.6.0)
##  hms           0.5.3   2020-01-08 [1] CRAN (R 3.6.2)
##  htmltools     0.4.0   2019-10-04 [1] CRAN (R 3.6.2)
##  httr          1.4.1   2019-08-05 [1] CRAN (R 3.6.2)
##  inline        0.3.15  2018-05-18 [1] CRAN (R 3.6.0)
##  jsonlite      1.6.1   2020-02-02 [1] CRAN (R 3.6.2)
##  knitr         1.27    2020-01-16 [1] CRAN (R 3.6.2)
##  labeling      0.3     2014-08-23 [1] CRAN (R 3.6.0)
##  lattice       0.20-38 2018-11-04 [4] CRAN (R 3.5.1)
##  lazyeval      0.2.2   2019-03-15 [1] CRAN (R 3.6.0)
##  lifecycle     0.1.0   2019-08-01 [1] CRAN (R 3.6.1)
##  loo           2.2.0   2019-12-19 [1] CRAN (R 3.6.2)
##  lubridate     1.7.4   2018-04-11 [1] CRAN (R 3.6.0)
##  magrittr    * 1.5     2014-11-22 [1] CRAN (R 3.6.0)
##  matrixStats   0.55.0  2019-09-07 [1] CRAN (R 3.6.2)
##  memoise       1.1.0   2017-04-21 [1] CRAN (R 3.6.0)
##  modelr        0.1.5   2019-08-08 [1] CRAN (R 3.6.2)
##  munsell       0.5.0   2018-06-12 [1] CRAN (R 3.6.0)
##  nlme          3.1-144 2020-02-06 [4] CRAN (R 3.6.2)
##  pillar        1.4.3   2019-12-20 [1] CRAN (R 3.6.2)
##  pkgbuild      1.0.6   2019-10-09 [1] CRAN (R 3.6.2)
##  pkgconfig     2.0.3   2019-09-22 [1] CRAN (R 3.6.1)
##  pkgload       1.0.2   2018-10-29 [1] CRAN (R 3.6.0)
##  prettyunits   1.1.1   2020-01-24 [1] CRAN (R 3.6.2)
##  processx      3.4.1   2019-07-18 [1] CRAN (R 3.6.1)
##  ps            1.3.0   2018-12-21 [1] CRAN (R 3.6.0)
##  purrr       * 0.3.3   2019-10-18 [1] CRAN (R 3.6.1)
##  R6            2.4.1   2019-11-12 [1] CRAN (R 3.6.2)
##  Rcpp          1.0.3   2019-11-08 [1] CRAN (R 3.6.2)
##  readr       * 1.3.1   2018-12-21 [1] CRAN (R 3.6.0)
##  readxl        1.3.1   2019-03-13 [1] CRAN (R 3.6.0)
##  remotes       2.1.0   2019-06-24 [1] CRAN (R 3.6.1)
##  reprex        0.3.0   2019-05-16 [1] CRAN (R 3.6.0)
##  rlang         0.4.4   2020-01-28 [1] CRAN (R 3.6.2)
##  rmarkdown     2.1     2020-01-20 [1] CRAN (R 3.6.2)
##  rprojroot     1.3-2   2018-01-03 [1] CRAN (R 3.6.0)
##  rstan       * 2.19.2  2019-07-09 [1] CRAN (R 3.6.1)
##  rstudioapi    0.10    2019-03-19 [1] CRAN (R 3.6.0)
##  rvest         0.3.5   2019-11-08 [1] CRAN (R 3.6.2)
##  scales        1.1.0   2019-11-18 [1] CRAN (R 3.6.2)
##  sessioninfo   1.1.1   2018-11-05 [1] CRAN (R 3.6.0)
##  StanHeaders * 2.19.0  2019-09-07 [1] CRAN (R 3.6.2)
##  stringi       1.4.5   2020-01-11 [1] CRAN (R 3.6.2)
##  stringr     * 1.4.0   2019-02-10 [1] CRAN (R 3.6.0)
```

```
##  testthat      2.3.1   2019-12-01 [1] CRAN (R 3.6.2)
##  tibble      * 2.1.3   2019-06-06 [1] CRAN (R 3.6.0)
##  tidyr       * 1.0.2   2020-01-24 [1] CRAN (R 3.6.2)
##  tidyselect    1.0.0   2020-01-27 [1] CRAN (R 3.6.2)
##  tidyverse   * 1.3.0   2019-11-21 [1] CRAN (R 3.6.2)
##  usethis       1.5.1   2019-07-04 [1] CRAN (R 3.6.1)
##  vctrs         0.2.2   2020-01-24 [1] CRAN (R 3.6.2)
##  viridisLite   0.3.0   2018-02-01 [1] CRAN (R 3.6.0)
##  withr         2.1.2   2018-03-15 [1] CRAN (R 3.6.0)
##  xfun          0.12    2020-01-13 [1] CRAN (R 3.6.2)
##  xml2          1.2.2   2019-08-09 [1] CRAN (R 3.6.2)
##  yaml          2.2.1   2020-02-01 [1] CRAN (R 3.6.2)
##
## [1] /home/ghazi/R/x86_64-pc-linux-gnu-library/3.6
## [2] /usr/local/lib/R/site-library
## [3] /usr/lib/R/site-library
## [4] /usr/lib/R/library
```
