# Supplementary material for: Bayesian modelling of high-throughput sequencing assays with malacoda
Source: PLoS Comput Biol. 2020 Jul 21;16(7):e1007504. doi: 10.1371/journal.pcbi.1007504 (PMC7394446; doi:10.1371/journal.pcbi.1007504)
Supplement: S3 Appendix — (PDF) [file pcbi.1007504.s003.pdf]

malacoda simulations

Andrew Ghazi

Contents

1 Goal **1**

2 Core simulation functions **1**

3 Define Simulation grid **6**

4 Evaluate methods on simulated data **7**

4.1 Run malacoda on simulations 7
4.2 Run t-test on simulations 13
4.3 Run mpralm on simulations 15
4.4 Run MPRAscore on simulations 19
4.5 Run QuASAR-MPRA on simulations 21
4.6 Run MPRAnalyze on simulations 24

5 Compare all methods **26**

6 malacoda regularization **36**

7 Conclusion **37**

8 Session Info **38**

1 Goal

This document describes the simulations used to statistical gains from using the malacoda method of MPRA analysis under ideal situations, while varying important parameters. Simulation is a key tool in computational studies because it permits the evaluation of methods under controlled statistical settings. For this study our objective is to examine the performance of malacoda compared to the alternative MPRA analysis methods. To construct the simulations requires the identification of the key parameters in the MPRA problem. These parameters are:

- assay size (number of variants)
- barcodes per allele
- dispersion
- effect size distribution (and fraction of truly functional variants)
- and for the use of conditional priors, the strength of the association between transcription shift and a single functional predictor.

2 Core simulation functions

Simulation assumptions used in this study which are consistent with real experimental MPRA studies:

- there are 3 DNA sequencing samples
- 6 RNA sequencing samples
- 200 million reads in the NGS assay in total

Using the assumptions above to generate an example MPRA dataset with 3000 variants and 14 barcodes per allele, with 15% failed barcodes, there are on average 200M / 9 * 0.85 / 3000 / (14*2) = 224 reads of a single barcode in a single sample.

This function simulates the sequencing depth of an assay across samples. The `rdirichlet` alpha parameters are chosen by inspection. It assumes that the 9 multiplexed samples are evenly sequenced on average, and that the per-sample depths typically range from about 15M to 30M:

```
library(tidyverse)
library(magrittr)
simulate_depth_factors = function(depth_multiplier = 1e6,
                                  tot_reads = 200e6,
                                  n_dna = 3,
                                  n_rna = 6,
                                  conc_par = 30){
  tibble(sample_id = c(paste0('DNA_', 1:n_dna),
                       paste0('RNA_', 1:n_rna)),
         depth_factor = as.vector(floor(gtools::rdirichlet(1, alpha = rep(conc_par, n_dna + n_rna)) * t[illegible]
           depth_multiplier))
}

depth_sim = {200e6*gtools::rdirichlet(50000, alpha = rep(30, 9))} %>%
  set_colnames(1:9) %>%
  as_tibble %>%
  gather(sample_id, depth)

depth_sim %>%
  ggplot(aes(depth)) +
  geom_histogram(aes(y = ..density..), bins = 50) +
  geom_vline(lty = 2,
             xintercept = quantile(depth_sim$depth, prob = c(.025, .975))) +
  theme_light() +
  labs(title = 'Sampling distribution of assay sample depths',
       subtitle = '95% interval: 15.3M - 30.2M',
       x = 'Depth of a single sample')
```

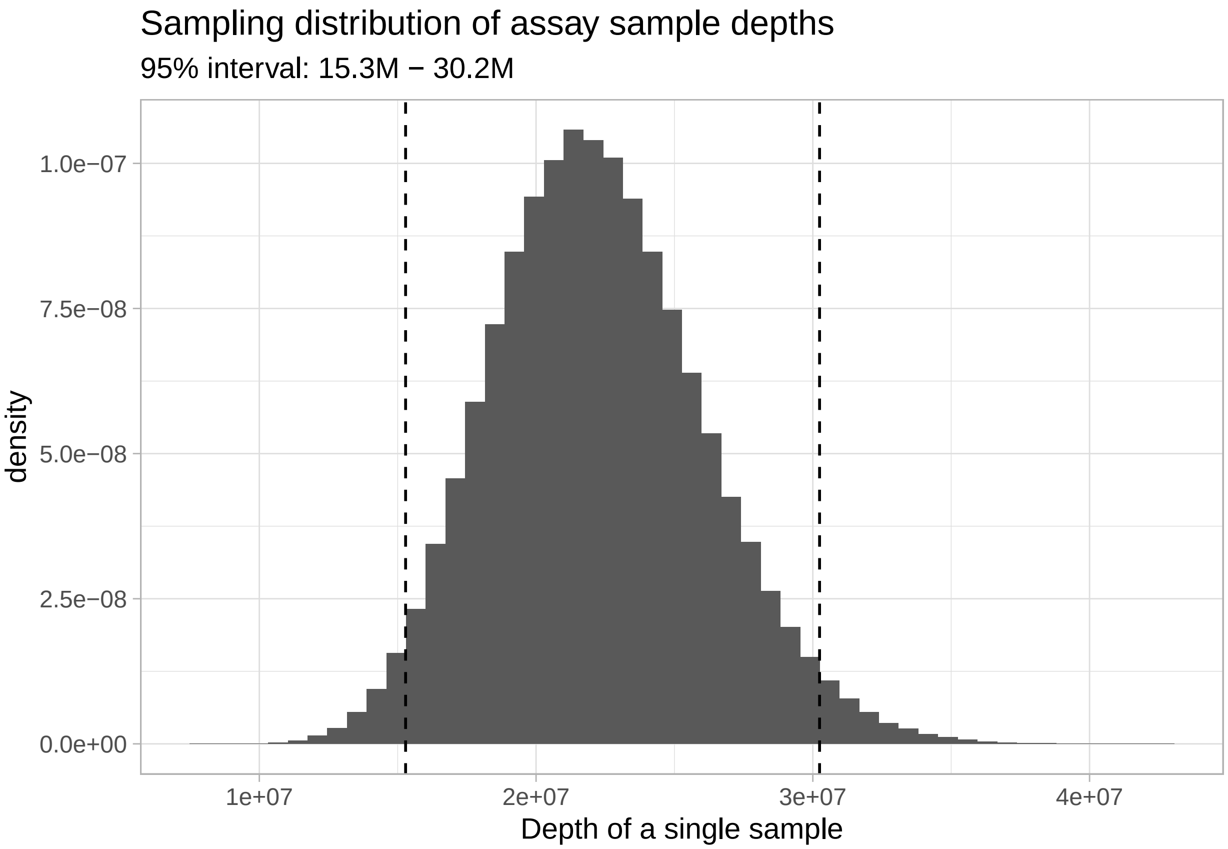

Th code block below defines a function to simulate a single variant and a function to simulate an entire assay by repeatedly calling the single variant code. By default the method assumes that roughly 15% of barcodes "fail" at some point during the oligonucleotide synthesis or sequencing library preparation stages, which is modelled as reducing their DNA concentration by a factor of 50. The other simulation parameters are randomly drawn from appropriate gamma distributions. These are:

- $\mu_{DNA,bc} \sim Gamma(\alpha = 3.42, \beta = .203)$
- $\mu_{RNA,ref} \sim Gamma(\alpha = 3, \beta = 3)$
- $\mu_{RNA,alt} \sim exp(ES) * \mu_{RNA,ref}$
- $\phi_{DNA} \sim Gamma(\alpha = 18.3, \beta = 16.7)$
- $\phi_{RNA,ref} \sim Gamma(\alpha = 7.43, \beta = 8.99)$
- $\phi_{RNA,alt} \sim Gamma(\alpha = 7.62, \beta = 9.41)$

The α and β parameters were chosen from inspection based on the marginal prior from our primary MPRA dataset.

`simulate_variant()` simulates a single variant. `simulate_assay()` assigns random effect sizes and calls `simulate_variant()` a set number of times. The effect sizes ES in `simulate_assay()` are set to 0 at a defined fraction (1 - `p_func`), and the remainder drawn from +/- 1 times a random draw from $Gamma(\alpha = 2, \beta = 2)$ distribution.

```
initial_depths = simulate_depth_factors()

simulate_draws = function(n, means, disp){
  map_dbl(means, ~rnbinom(n = n,
                          mean = .x,
                          size = disp))
}
```

```r
simulate_variant = function(variant_id ,
                            effect_size, # input using log-scale, it get's exp()'d inside
                            depth_factors, # depth factors of the samples
                            n_barcodes = 14,
                            dna_mu = rgamma(n_barcodes * 2, shape = 3.42, rate = .203),
                            dna_phi = rgamma(1, shape = 18.4, rate = 16.7),
                            rna_mu = rgamma(1, shape = 3, rate = 3),
                            ref_rna_phi = rgamma(1, shape = 7.43, rate = 8.99),
                            alt_rna_phi = rgamma(1, shape = 7.62, rate = 9.41),
                            fail_rate = 0.15,
                            fail_reduction = 50){

  ref_rna_mu = rna_mu
  alt_rna_mu = exp(effect_size) * rna_mu

  out_df = tibble(allele = c(rep('ref', n_barcodes),
                             rep('alt', n_barcodes)),
                  bc_id = 1:(2*n_barcodes),
                  dna_means = dna_mu,
                  dna_size = dna_phi,
                  rna_mean = rep(c(ref_rna_mu, alt_rna_mu), each = n_barcodes),
                  rna_phi = rep(c(ref_rna_phi, alt_rna_phi), each = n_barcodes),
                  failed = runif(2*n_barcodes) < fail_rate)

  out_df$dna_means[out_df$failed] = 1/fail_reduction * out_df$dna_means[out_df$failed]

  mean_by_cell = outer(Y = depth_factors %>%
                         filter(grepl('DNA', sample_id)) %>%
                         pull(depth_factor),
                       X = out_df$dna_means) %>%
    set_colnames( depth_factors %>%
                    filter(grepl('DNA', sample_id)) %>% pull(sample_id)) %>%
    as_tibble %>%
    mutate(bc_id = 1:(2*n_barcodes)) %>%
    gather(sample_id, depth_mean, -bc_id)

  dna_draws = mean_by_cell %>% mutate(draws = map_dbl(depth_mean, ~rnbinom(n = 1, mu = .x,
                                                                         size = dna_phi))) %>%
    dplyr::select(-depth_mean) %>%
    spread(sample_id, draws)

  rna_adjusts = tibble(bc_id = 1:(2*n_barcodes),
                       rna_adj = rep(c(ref_rna_mu, alt_rna_mu),
                                     each = n_barcodes))

  disp_by_id = tibble(bc_id = 1:(2*n_barcodes),
                      disp = c(rep(ref_rna_phi, n_barcodes),
                               rep(alt_rna_phi, n_barcodes)))

  rna_mean_by_cell = outer(Y = depth_factors %>%
                             filter(grepl('RNA', sample_id)) %>%
                             pull(depth_factor),
                           X = out_df$dna_means) %>%
```

```r
    set_colnames( depth_factors %>%
                    filter(grepl('RNA', sample_id)) %>% pull(sample_id)) %>%
    as_tibble %>%
    mutate(bc_id = 1:(2*n_barcodes)) %>%
    gather(sample_id, depth_mean, -bc_id)

  rna_draws = rna_mean_by_cell %>%
    left_join(rna_adjusts, by = 'bc_id') %>%
    mutate(rna_depth_mean = depth_mean * rna_adj) %>%
    dplyr::select(-depth_mean, -rna_adj) %>%
    left_join(disp_by_id, by = 'bc_id') %>%
    mutate(draws = map2_dbl(rna_depth_mean,
                            disp,
                           ~rnbinom(1, mu = .x, size = .y))) %>%
    dplyr::select(-rna_depth_mean, -disp) %>%
    spread(sample_id, draws)

  out_df %<>%
    mutate(variant_id = variant_id) %>%
    left_join(dna_draws, by = 'bc_id') %>%
    left_join(rna_draws, by = 'bc_id')

  return(out_df)
}

simulate_assay = function(n_variants = 5000,# number of variants in the MPRA
                          p_func = .05, # probability of functionality
                          n_bpa){  # number barcodes per allele
  initial_depths = simulate_depth_factors()

  tibble(variant_id = map_chr(1:n_variants, ~paste0(sample(letters, size = 8), collapse = '')),
         truly_functional = runif(n_variants) < p_func,
         effect_size = truly_functional *
           sample(c(-1,1), size = n_variants, replace = TRUE) *
           rgamma(n_variants, shape = 2, rate = 2),
         variant_data = map2(variant_id, effect_size,
                             simulate_variant,
                             depth_factors = initial_depths,
                             n_barcodes = n_bpa))
}
```

The next two tables show one example call to `simulate_depth_factors` followed by one call to `simulate_variant()` with effect size -1 and 5 barcodes per allele.

```r
set.seed(1234)
knitr::kable(initial_depths)
```

sample_id	depth_factor
DNA_1	24.66545
DNA_2	22.93912
DNA_3	25.50553
RNA_1	21.72887
RNA_2	19.85586
RNA_3	16.90123

sample_id	depth_factor
RNA_4	22.47062
RNA_5	21.16074
RNA_6	24.77257

```
simulate_variant(variant_id = 'test_variant',
                 effect_size = -1,
                 depth_factors = initial_depths, n_barcodes = 5) %>%
  select(-variant_id, -bc_id) %>%
  knitr::kable(digits = 3, format =  'latex') %>%
  kableExtra::kable_styling(full_width = TRUE, font_size = 6)
```

allele	dna_mean	dna_size	rna_mean	rna_phi	failed	DNA_1	DNA_2	DNA_3	RNA_1	RNA_2	RNA_3	RNA_4	RNA_5	RNA_6
ref	17.152	1.329	0.319	0.739	FALSE	264	61	91	93	13	170	59	347	354
ref	17.568	1.329	0.319	0.739	FALSE	516	520	164	363	3	12	148	256	134
ref	8.892	1.329	0.319	0.739	FALSE	29	122	87	93	79	14	48	2	137
ref	18.959	1.329	0.319	0.739	FALSE	805	134	158	19	90	66	127	981	15
ref	9.953	1.329	0.319	0.739	FALSE	468	133	618	7	26	230	76	4	60
alt	23.853	1.329	0.117	0.762	FALSE	1147	335	235	12	68	115	239	11	126
alt	9.622	1.329	0.117	0.762	FALSE	417	193	437	14	31	25	4	0	0
alt	10.648	1.329	0.117	0.762	FALSE	29	176	310	22	25	15	58	108	8
alt	3.422	1.329	0.117	0.762	FALSE	30	30	64	3	3	13	0	1	15
alt	14.932	1.329	0.117	0.762	FALSE	259	250	421	19	6	10	55	48	34

3 Define Simulation grid

The code block below defines a parameter grid, altering the following values:

- `p_func` - the fraction of simulated variants that are truly functional
  - values: 0, 0.05, 0.1
- `n_variants` - the total number of simulated variants in each simulated assay
  - values: 100, 300, 1000, 3000
- `n_bpa` - the number of barcodes per allele assigned to each simulated allele of each variant
  - values: 10, 30

```
sim_grid = expand.grid(list(p_func = c(.0, .05, .1),
                            n_variants = c(300, 1000, 3000),
                            n_bpa = c(10, 30))) %>%
  as_tibble %>%
  mutate(point_ids = map2(p_func, n_variants, ~tibble(sim_id = map_chr(1:100, ~paste0(sample(letters, s
  unnest

save(sim_grid,
     file = '/mnt/bigData2/andrew/MPRA/sim_mpra/many_sims/sim_grid.RData')

sim_once_save = function(sim_id, p_func, n_variants, n_bpa){
  current_sim = simulate_assay(p_func = p_func, n_variants = n_variants, n_bpa = n_bpa)

  save(current_sim,
       file = paste0('/mnt/bigData2/andrew/MPRA/sim_mpra/many_sims/', sim_id, '.RData'))

  return('donesk')
}

sim_grid %>%
  mutate(make_sims = parallel::mcmapply(sim_once_save,
```

```
                                             sim_id, p_func, n_variants, n_bpa,
                                             mc.preschedule = FALSE,
                                             mc.cores = 16))

print('SUPER DONESK')
```

4 Evaluate methods on simulated data

Each subsection below runs a given MPRA analysis method on the simulated assays (or a substantial subset of them for malacoda and MPRAnalyze, the two slowest methods) and assesses their performance according to the four metrics outlined in the "Compare all methods" section below.

4.1 Run malacoda on simulations

The code block below fits the malacoda model (marginal prior) on a random subset of the simulation grid using a marginal prior and 10000 posterior samples. The outputs for a single large simulation are on the order of 10 GB, so we instead only analyze 26 simulated assays from each simulation grid point.

```
library(tidyverse)
library(magrittr)
library(malacoda)

load('/mnt/bigData2/andrew/MPRA/sim_mpra/many_sims/sim_grid.RData')

malacoda_sim = function(sim_id){
  # make the output directory
  sim_out_dir = paste0('/mnt/bigData2/andrew/MPRA/sim_mpra/many_sims_malacoda/', sim_id)
  dir.create(sim_out_dir)

  # load the simulated assay
  load(paste0('/mnt/bigData2/andrew/MPRA/sim_mpra/many_sims/', sim_id, '.RData'))

  # malacoda needs a barcode column, so add one
  fake_bcs = sample(x = c('A', 'C', 'G', 'T'),
                    size = nrow(unnest_legacy(current_sim))*16,
                    replace = TRUE) %>%
    matrix(nrow = nrow(unnest_legacy(current_sim))) %>%
    apply(MARGIN = 1, FUN = paste0, collapse = '')

  if (n_distinct(fake_bcs) < nrow(current_sim)){
    while (n_distinct(fake_bcs) < nrow(current_sim)) {
      fake_bcs = sample(x = c('A', 'C', 'G', 'T'),
                        size = nrow(current_sim)*16,
                        replace = TRUE) %>%
        matrix(nrow = nrow(current_sim)) %>%
        apply(MARGIN = 1, FUN = paste0, collapse = '')
    }
  }

  # Format simulated mpra data
  md = current_sim %>%
```

```r
    unnest_legacy %>%
    mutate(barcode = fake_bcs) %>%
    select(variant_id, allele, barcode, matches('[DR]NA', ignore.case = FALSE))

  sim_sd = get_sample_depths(md)

  wr = get_well_represented(md,
                            sample_depths = sim_sd,
                            rep_cutoff = .15,
                            plot_rep_cutoff = FALSE,
                            verbose = FALSE)

  # fit the marginal prior
  sim_prior = fit_marg_prior(md,
                             n_cores = 16,
                             rep_cutoff = .15,
                             plot_rep_cutoff = FALSE)

  # fit the model
  model_fit = fit_mpra_model(md,
                             n_cores = 16,
                             save_nonfunctional = TRUE,
                             tot_samp = 10000,
                             n_warmup = 500,
                             vb_pass = FALSE,
                             verbose = FALSE,
                             rep_cutoff = .15,
                             out_dir = sim_out_dir,
                             priors = sim_prior)


  return('done :)')
}

sim_grid %>%
  group_by(p_func, n_variants, n_bpa) %>%
  do(head(., 26)) %>%
  mutate(group_index = 1:n()) %>% # Hoping to make the timing uniform
  ungroup %>%
  arrange(group_index) %>%
  mutate(malacoda_fit = parallel::mclapply(sim_id,
                                           malacoda_sim,
                                           mc.cores = 10,
                                           mc.preschedule = FALSE))
```

The code block below evaluates malacoda's performance on each simulated assay.

```r
library(tidyverse)
library(magrittr)
library(malacoda)
library(pROC)

load('/mnt/bigData2/andrew/MPRA/sim_mpra/many_sims/sim_grid.RData')
```

```r
variant_cutoff = function(sim_id, variant_id){
  # for a given variant, this function returns the TS HDI threshold necessary to call it functional

  load(paste0('/mnt/bigData2/andrew/MPRA/sim_mpra/many_sims_malacoda/', sim_id, '/', variant_id, '.RData[illegible]

  ts_samples = sampler_res %>% rstan::extract(pars = 'transcription_shift') %>% .$transcription_shift

  above_zero = rstan::summary(sampler_res)$summary['transcription_shift', 'mean'] > 0

  # If the variant has a negative posterior shift, you need to see how wide the
  # HDI needs to be to make the region ABOVE zero plausible. Otherwise it's vice
  # versa.
  if(above_zero){
    frac_opposite = sum(ts_samples < 0) / length(ts_samples)
  } else {
    frac_opposite = sum(ts_samples > 0) / length(ts_samples)
  }

  if (frac_opposite > .5) {
    frac_opposite = 1 - frac_opposite
  }

  necessary_cutoff = 1 - 2*frac_opposite # The width of the HDI necessary to include 0

  return(necessary_cutoff)
}

malacoda_performance = function(sim_id){

  load(paste0('/mnt/bigData2/andrew/MPRA/sim_mpra/many_sims_malacoda/', sim_id, '/analysis_res.RData'))
  load(paste0('/mnt/bigData2/andrew/MPRA/sim_mpra/many_sims/', sim_id, '.RData'))


  file_check = file.exists(paste0('/mnt/bigData2/andrew/MPRA/sim_mpra/many_sims_malacoda/', sim_id, '/j[illegible]

  if (file_check){
    load(paste0('/mnt/bigData2/andrew/MPRA/sim_mpra/many_sims_malacoda/', sim_id, '/joined_df.RData'))

    typical_table =  joined_df %>%
      select(truly_functional, is_functional) %>%
      table
  } else{
    joined_df = analysis_res %>%
      select(-`variant_data`) %>%
      left_join(current_sim,
                by = 'variant_id')

    typical_table =  joined_df %>%
      select(truly_functional, is_functional) %>%
      table

    joined_df %<>% # this is slow, save the result just in case
      mutate(hdi_cutoff = map_dbl(variant_id,
```

```r
                                   variant_cutoff,
                                   sim_id = sim_id))

    save(joined_df,
         file = paste0('/mnt/bigData2/andrew/MPRA/sim_mpra/many_sims_malacoda/', sim_id, '/joined_df.RDa

  }

  if (!any(joined_df$truly_functional)){
    auc_val = NA
    aupr = NA
  } else {
    roc_obj = pROC::roc(response = joined_df$truly_functional,
                        predictor = joined_df$hdi_cutoff)
    auc_val = pROC::auc(roc_obj);

    approx_df = joined_df %>%
      mutate(all_over = hdi_cutoff == 1 | hdi_cutoff == 0)

    approx_lm = approx_df %>%
      filter(!all_over) %>%
      lm(atanh(hdi_cutoff) ~ abs(ts_post_mean),
         data = .) %>%
      summary

    approx_coef = approx_lm$coefficients

    # approx_df %>%
    #   ggplot(aes(abs(ts_post_mean), hdi_cutoff)) +
    #   geom_point(aes(color = all_over)) +
    #   scale_y_continuous(trans = 'atanh') +
    #   geom_smooth(method = 'lm')

    # ^ tanh(-.13 + 7.05*abs(ts_post_mean)) is a pretty good approximation of the 1- "p-value"

    approx_df %<>%
      mutate(approx_cutoff = case_when(!all_over ~ hdi_cutoff,
                                       TRUE ~ tanh(approx_coef[2,'Estimate']*abs(ts_post_mean) - approx_

    approx_roc_obj = pROC::roc(predictor = 1-approx_df$approx_cutoff,
                               response = approx_df$truly_functional)

    approx_coords = pROC::coords(approx_roc_obj,
                                 ret = c('recall', 'precision'),
                                 transpose = FALSE)

    # Compute AUPR using the trapezoidal rule
    pr_mat = approx_coords %>%  .[nrow(.):1,] %>% .[!duplicated(.$recall),]
    nrow_pr = nrow(pr_mat)
    drecall = pr_mat[2:nrow_pr,1] - pr_mat[1:(nrow_pr - 1),1]
    avg_precision = (pr_mat[2:nrow_pr, 2] + pr_mat[1:(nrow_pr - 1), 2]) / 2
    avg_precision[1] = pr_mat[2,2] # the first precision value is returned as NaN -- set to next value
    daupr = drecall * avg_precision
```

```r
    aupr = sum(daupr)
  }


  spread_at_zero = joined_df %>%
    filter(!truly_functional) %>%
    pull(ts_post_mean) %>%
    sd

  nonzero_cor = joined_df %>%
    filter(truly_functional) %$%
    cor(effect_size, ts_post_mean);

  res_df = tibble(auc = auc_val,
                  aupr = aupr,
                  spread_at_zero = spread_at_zero,
                  nonzero_cor = nonzero_cor,
                  typical_table = list(typical_table))

  return(res_df)
}

malacoda_perf = list.files('/mnt/bigData2/andrew/MPRA/sim_mpra/many_sims_malacoda') %>%
  pbmcapply::pbmclapply(malacoda_performance,
                     mc.cores = 16,
                     mc.preschedule = FALSE)


malacoda_perf = tibble(sim_id = list.files('/mnt/bigData2/andrew/MPRA/sim_mpra/many_sims_malacoda')) %>%
  mutate(perf_res = malacoda_perf) %>%
  unnest_legacy()

save(malacoda_perf,
     file = '~/dev_malacoda/outputs/malacoda_perf.RData')
```

In order to calculate the ROC and precision-recall metrics, we needed to derive a quantity similar to a p-value for each variant. This was done in two steps:

- define the minimal HDI probability mass that would be necessary to call the variant non-functional (the bold black line in the demonstrative figure below). This is the “HDI cutoff”.
- take 1 - the HDI cutoff to define p_i, the pseudo-pvalue for variant i. In the demonstrative figure, variant 1 requires a 31% HDI cutoff and variant 2 requires 97.2%, so this gives $p_1 = 0.689$ and $p_2 = 0.028$.

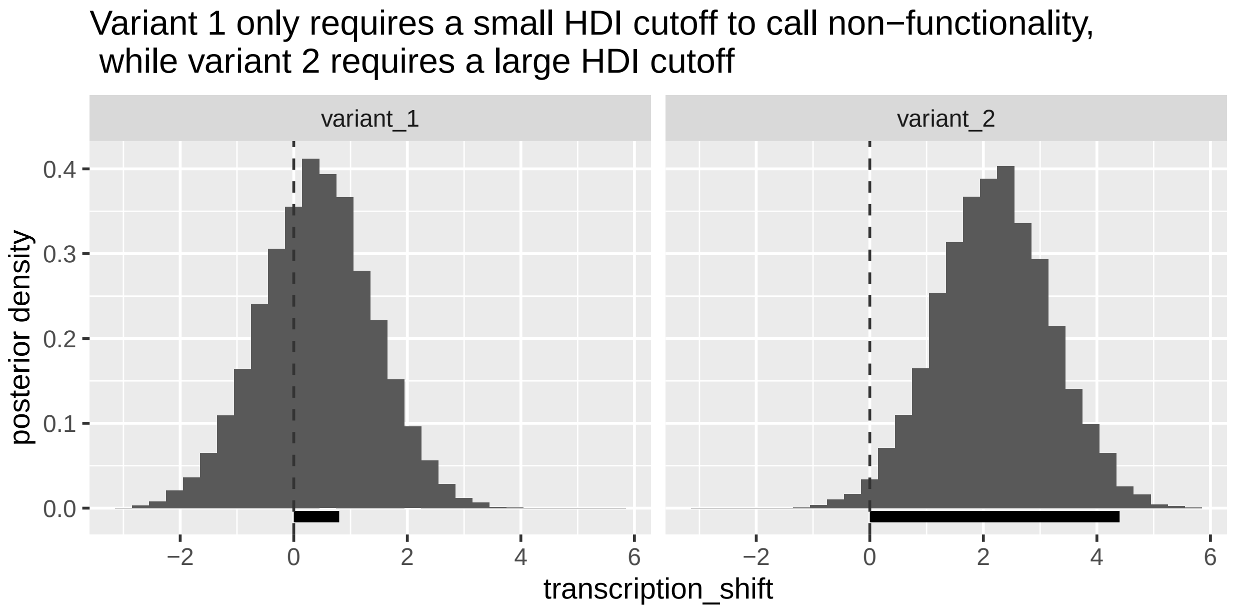

The fact that malacoda is fit with MCMC adds an additional complication to this procedure: some simulated variants with very strong effects will have all posterior samples of TS on one side of zero. This will lead to p_i being exactly 0 or exactly 1, which in turn produces an incomplete precision-recall curve. In order to mitigate this, for variants falling into this category we use a linear approximation based off the relationship between the absolute estimated effect size and atanh(minimal HDI). In the figure below, we use the black line trained on the orange dots, to approximate the HDI cutoff needed for the blue dots. It can be seen that the orange points are gently curving upward above the approximation, so the extrapolated HDI cutoffs will tend to be conservative.

```
sim_id = "zwnxvebu"

load(paste0('/mnt/bigData2/andrew/MPRA/sim_mpra/many_sims_malacoda/', sim_id, '/joined_df.RData'))

approx_lm = joined_df %>%
  filter(!(hdi_cutoff %in% c(0,1))) %>%
  lm(data = .,
     atanh(hdi_cutoff) ~ abs(ts_post_mean)) %>%
  summary

joined_df %>%
  mutate(needs_approximation = hdi_cutoff %in% c(0,1)) %>%
  ggplot(aes(abs(ts_post_mean),
             atanh(hdi_cutoff))) +
  geom_point(aes(color = needs_approximation)) +
  geom_abline(slope = approx_lm$coefficients[2,1],
              intercept = approx_lm$coefficients[1,1])+
  theme_light()
```

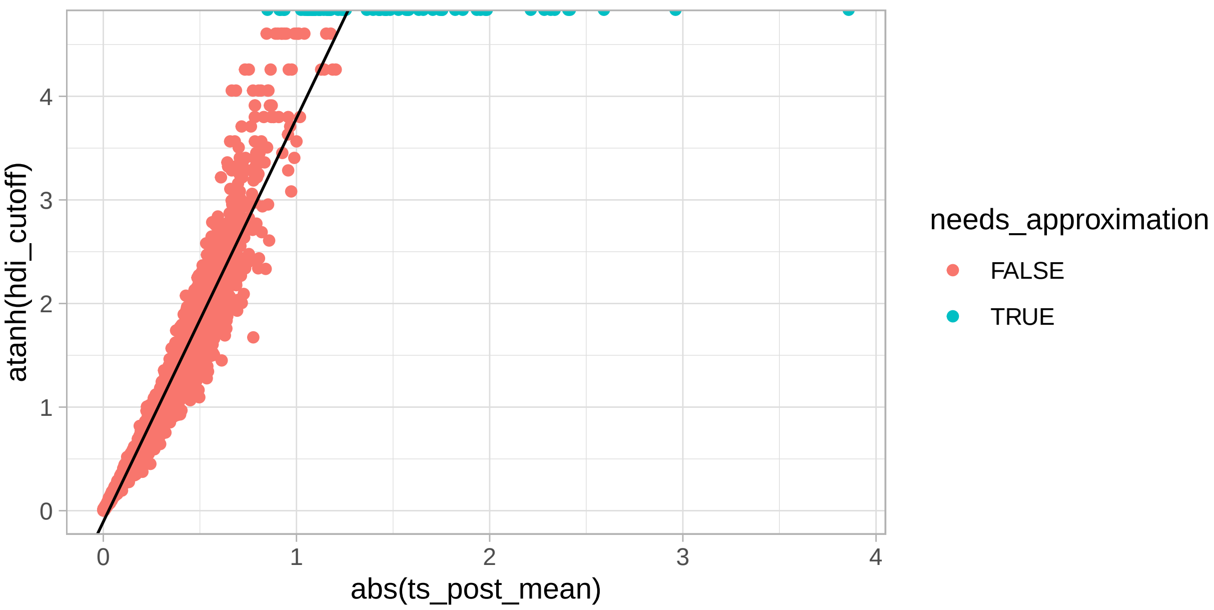

4.2 Run t-test on simulations

The code block below executes malacoda's implementation of the activity t-test method to analyze the simulations and assess the performance in each.

```
library(tidyverse)
library(malacoda)
library(pROC)

load('/mnt/bigData2/andrew/MPRA/sim_mpra/many_sims/sim_grid.RData')

get_t_sim_performance = function(sim_id){
  load(paste0('/mnt/bigData2/andrew/MPRA/sim_mpra/many_sims/', sim_id, '.RData'))

  fake_bcs = sample(x = c('A', 'C', 'G', 'T'),
                    size = nrow(unnest_legacy(current_sim))*16,
                    replace = TRUE) %>%
    matrix(nrow = nrow(unnest_legacy(current_sim))) %>%
    apply(MARGIN = 1, FUN = paste0, collapse = '')

  if (n_distinct(fake_bcs) < nrow(current_sim)){
    while (n_distinct(fake_bcs) < nrow(current_sim)) {
      fake_bcs = sample(x = c('A', 'C', 'G', 'T'),
                        size = nrow(current_sim)*16,
                        replace = TRUE) %>%
        matrix(nrow = nrow(current_sim)) %>%
        apply(MARGIN = 1, FUN = paste0, collapse = '')
    }
  }

  # Format simulated mpra data
  md = current_sim %>%
    unnest_legacy() %>%
    mutate(barcode = fake_bcs) %>%
```

```r
  select(variant_id, allele, barcode, matches('[DR]NA', ignore.case = FALSE))

sim_sd = get_sample_depths(md)

wr = get_well_represented(md,
                          sample_depths = sim_sd,
                          rep_cutoff = .15,
                          plot_rep_cutoff = FALSE,
                          verbose = FALSE)

precomputed_check = file.exists(paste0('/mnt/bigData2/andrew/MPRA/sim_mpra/many_sims_t/', sim_id, '_te

if (precomputed_check){
  load(paste0('/mnt/bigData2/andrew/MPRA/sim_mpra/many_sims_t/', sim_id, '_test_df.RData'))
} else{

  act_df = compute_activities(md,
                              rep_cutoff = .15,
                              plot_rep_cutoff = FALSE)

  test_df = run_activity_tests(act_df,
                               plot_p_hist = FALSE)

  save(test_df,
       file = paste0('/mnt/bigData2/andrew/MPRA/sim_mpra/many_sims_t/', sim_id, '_test_df.RData'))
}


if (any(current_sim$truly_functional)){
  perf_input = test_df %>%
    left_join(current_sim, by = 'variant_id') %>%
    mutate(fdr_hit = q_value < .05) %>%
    ungroup

    perf_df = perf_input %>%
    summarise(auc = auc(roc(predictor = .data$p.value,
                            response = .data$truly_functional,
                            quiet = TRUE)),
              pr = list(pROC::coords(roc(predictor = .data$p.value,
                            response = .data$truly_functional,
                            quiet = TRUE),
                          ret = c('recall', 'precision'),
                          transpose = FALSE)),
              mean_nzc = cor(ts_estimate[truly_functional], #mean non-zero correlation
                             effect_size[truly_functional]),
              mean_saz = sd(ts_estimate[!truly_functional]),
              typical_table = list(table(truly_functional,
                                         fdr_hit))) # mean spread at zero

    # Compute AUPR using the trapezoidal rule
    pr_mat = perf_df$pr[[1]] %>% .[nrow(.):1,] %>% .[!duplicated(.$recall),]
    nrow_pr = nrow(pr_mat)
    drecall = pr_mat[2:nrow_pr,1] - pr_mat[1:(nrow_pr - 1),1]
```

```
      avg_precision = (pr_mat[2:nrow_pr, 2] + pr_mat[1:(nrow_pr - 1), 2]) / 2
      avg_precision[1] = pr_mat[2,2] # the first precision value is returned as NaN -- set to next valu
      daupr = drecall * avg_precision
      aupr = sum(daupr)

      perf_df$pr = NULL
      perf_df$aupr = aupr
  } else {
    perf_df =
      test_df %>%
      left_join(current_sim, by = 'variant_id') %>%
      mutate(fdr_hit = q_value < .05) %>%
      ungroup %>%
      summarise(auc = NA,
                mean_nzc = NA,
                mean_saz = sd(ts_estimate[!truly_functional]),
                typical_table = list(table(truly_functional,
                                           fdr_hit))) # mean spread at zero
  }

  save(perf_df,
       file = paste0('/mnt/bigData2/andrew/MPRA/sim_mpra/many_sims_t/', sim_id, '_perf_df.RData'))

  return(perf_df)
}

t_perf = suppressWarnings(sim_grid %>%
                            mutate(t_perf_call = parallel::mclapply(sim_id,
                                                                    get_t_sim_performance,
                                                                    mc.cores = 20,
                                                                    mc.preschedule = FALSE)) %>%
                            unnest_legacy() %>%
                            arrange(p_func, n_variants, n_bpa))

save(t_perf,
     file = '/mnt/bigData2/andrew/MPRA/sim_mpra/t_performance.RData')
```

4.3 Run mpralm on simulations

The code block below executes mpralm on the simulation grid defined above. The mpralm requires summing across RNA samples to ensure that there are the same number of DNA and RNA samples.

```
library(tidyverse)
library(mpra)

mpralm_sim = function(sim_id){
  load(paste0('/mnt/bigData2/andrew/MPRA/sim_mpra/many_sims/', sim_id, '.RData'))

  mpra_data = current_sim %>% unnest()

  dna = mpra_data %>%
    select(variant_id, allele, matches('DNA')) %>%
  group_by(variant_id, allele) %>%
```

```
  summarise_at(paste0('DNA_', 1:3), sum) %>%
  ungroup %>% gather(sample_id, counts, matches('DNA')) %>%
  unite( col = 'allele_sample', allele, sample_id, sep = '_') %>%
  spread(allele_sample, counts) %>%
  column_to_rownames(var = 'variant_id') %>%
  as.matrix

rna = mpra_data %>%
    select(variant_id, allele, matches('RNA')) %>%
  group_by(variant_id, allele) %>%
  summarise_at(paste0('RNA_', 1:6), sum) %>%
  ungroup %>%
  gather(sample_id, counts, matches('RNA')) %>%
  mutate(sample_group = as.numeric(str_extract(sample_id, '[0-9]')) %% 3) %>%
  group_by(variant_id, allele, sample_group) %>%
  summarise(counts = sum(counts)) %>%
  unite( col = 'allele_sample', allele, sample_group, sep = '_') %>%
  spread(allele_sample, counts) %>%
  column_to_rownames(var = 'variant_id') %>%
  as.matrix %>%
  .[,1:6]


  element_ids = rownames(dna)
  colnames(rna) = colnames(dna)

  sim_mpra_set = MPRASet(DNA = dna,
                         RNA = rna,
                         eid = element_ids,
                         barcode = NULL,
                         eseq = NULL)

  design = data.frame(intcpt = 1,
                      alt_allele = grepl('alt', colnames(sim_mpra_set)))
  block_vector = rep(1:3, times = 2)

  mpralm_allele_fit <- mpralm(object = sim_mpra_set,
                              design = design,
                              aggregate = "none",
                              normalize = TRUE,
                              block = block_vector,
                              model_type = "indep_groups",
                              plot = TRUE)
  save(mpralm_allele_fit,
       file = paste0('/mnt/bigData2/andrew/MPRA/sim_mpra/many_sims_mpralm/', sim_id, '.RData'))

  return(':)')
}

load('/mnt/bigData2/andrew/MPRA/sim_mpra/many_sims/sim_grid.RData')

sim_grid %>%
  mutate(mpralm_res = parallel::mclapply(sim_id,
```

```
                                        mpralm_sim,
                                        mc.cores = 16,
                                        mc.preschedule = FALSE))
```

This code code block evaluates the performance of mpralm on the simulations.

```
library(magrittr)
library(pROC)
eval_mpralm_fit = function(sim_id){
  load(paste0('/mnt/bigData2/andrew/MPRA/sim_mpra/many_sims/', sim_id, '.RData'))
  load(paste0('/mnt/bigData2/andrew/MPRA/sim_mpra/many_sims_mpralm/', sim_id, '.RData'))

  p_df = mpralm_allele_fit$p.value %>%
    as.data.frame() %>%
    rownames_to_column('variant_id') %>%
    as_tibble %>%
    mutate(q_value = p.adjust(alt_allele, method = 'fdr')) %>%
    left_join(current_sim %>% select(-variant_data),
              by = 'variant_id') %>%
    dplyr::rename(intcpt_p = intcpt, alt_allele_p = alt_allele, alt_allele_q = q_value)

  top_table = mpralm_allele_fit %>% topTable(number = nrow(p_df)) %>%
    rownames_to_column(var = 'variant_id') %>%
    as_tibble %>%
    select(-(4:7))

  if (all(!current_sim$truly_functional)){
    res_df = p_df %>% left_join(top_table, by = 'variant_id')

    spread_at_0 = res_df %>%
      mutate(rescaled = log(2**alt_allele)) %>%
      filter(!truly_functional) %>%
      pull(rescaled) %>%
      sd

    typical_table = res_df %>%
      mutate(fdr_call = alt_allele_q < .05) %>%
      select(fdr_call, truly_functional) %>%
      table

    out_df = tibble(spread_at_0 = spread_at_0,
                    auc = NA,
                    nonzero_cor = NA,
                    aupr = NA,
                    `5%FDR_calls` = list(typical_table))
    return(out_df)
  }

  res_df = p_df %>% left_join(top_table, by = 'variant_id')

  roc_obj = roc(predictor = res_df$alt_allele_p,
        response = res_df$truly_functional, quiet = TRUE)

  auc_val = auc(roc_obj)
```

```r
  pr_obj = pROC::coords(roc_obj,
                ret = c('recall', 'precision'),
                transpose = FALSE)

  # Compute AUPR using the trapezoidal rule
  pr_mat = pr_obj %>% .[nrow(.):1,] %>% .[!duplicated(.$recall),]
  nrow_pr = nrow(pr_mat)
  drecall = pr_mat[2:nrow_pr,1] - pr_mat[1:(nrow_pr - 1),1]
  avg_precision = (pr_mat[2:nrow_pr, 2] + pr_mat[1:(nrow_pr - 1), 2]) / 2
  avg_precision[1] = pr_mat[2,2] # the first precision value is returned as NaN -- set to next value
  daupr = drecall * avg_precision
  aupr = sum(daupr)


  save(roc_obj, auc_val, aupr,
       file = paste0('/mnt/bigData2/andrew/MPRA/sim_mpra/many_sims_mpralm/', sim_id, '_roc.RData'))

  nonzero_cor = res_df %>%
    filter(truly_functional) %$%
    cor(alt_allele, effect_size)

  spread_at_0 = res_df %>%
    mutate(rescaled = log(2**alt_allele)) %>%
    filter(!truly_functional) %>%
    pull(rescaled) %>%
    sd

  save(nonzero_cor, spread_at_0,
       file = paste0('/mnt/bigData2/andrew/MPRA/sim_mpra/many_sims_mpralm/', sim_id, '_es.RData'))

  typical_table = res_df %>%
    mutate(fdr_call = alt_allele_q < .05) %>%
    select(fdr_call, truly_functional) %>%
    table

  out_df = tibble(spread_at_0 = spread_at_0,
                auc = auc_val,
                nonzero_cor = nonzero_cor,
                aupr = aupr,
                `5%FDR_calls` = list(typical_table))
  return(out_df)
}
```

```r
mpralm_sim_performance = sim_grid %>%
  mutate(eval_metrics = mclapply(sim_id, eval_mpralm_fit, mc.cores = 18)) %>%
  unnest_legacy

save(mpralm_sim_performance,
     file = '/mnt/bigData2/andrew/MPRA/sim_mpra/mpralm_sim_performance.RData')
```

4.4 Run MPRAscore on simulations

```
library(tidyverse)
library(magrittr)

mprascore_sim = function(sim_id){
  load(paste0('/mnt/bigData2/andrew/MPRA/sim_mpra/many_sims/', sim_id, '.RData'))

    fake_bcs = sample(x = c('A', 'C', 'G', 'T'),
                      size = nrow(unnest(current_sim))*18,
                      replace = TRUE) %>%
    matrix(nrow = nrow(unnest(current_sim))) %>%
    apply(MARGIN = 1, FUN = paste0, collapse = '')

  current_sim %<>%
    unnest_legacy %>%
    mutate(Barcode = fake_bcs)

  # make the "oligo" file

  og_table = current_sim %>%
    mutate(Oligo = paste(variant_id, allele, sep = '_'),
           Strand = '+',
           baseOffset = 0) %>%
    select(-matches('[DR]NA'), -Barcode, -bc_id, -failed, -variant_id1, -truly_functional, -effect_size
    unique %>%
    group_by(variant_id) %>%
    mutate(allele_letter = base::sample(c('A', 'C', 'G', 'T'), size  = 2, replace = FALSE)) %>%
    ungroup

  spread_table = og_table %>%
    select(variant_id, allele, allele_letter) %>%
    spread(allele, allele_letter)

  oligo_output = current_sim %>%
    mutate(Oligo = paste(variant_id, allele, sep = '_')) %>%
    left_join(spread_table, by = 'variant_id') %>%
    dplyr::rename(Ref = ref, Alt = alt) %>%
    mutate(Allele_in_oligo = case_when(allele == 'ref' ~ Ref,
                                       TRUE ~ Alt)) %>%
    mutate(Strand = '+',
           baseOffset = 0) %>%
    select(Oligo, RSID = variant_id, Ref, Alt, Allele_in_oligo, Strand, baseOffset) %>%
    unique

 oligo_out_path = paste0('/mnt/bigData2/andrew/MPRA/sim_mpra/many_sims_mprascore/', sim_id, '_oligos.tx
  write_tsv(oligo_output,
            path = oligo_out_path)

  # make the "bc counts" file

  bc_counts = current_sim %>%
    mutate(Oligo = paste(variant_id, allele, sep = '_')) %>%
```

```
    select(BC = Barcode, Oligo, matches('[DR]NA', ignore.case = FALSE))

  bc_out_path = paste0('/mnt/bigData2/andrew/MPRA/sim_mpra/many_sims_mprascore/', sim_id, '_bc_counts.t
  write_tsv(bc_counts,
            path = bc_out_path)

  # make output path & command

  out_path = paste0('/mnt/bigData2/andrew/MPRA/sim_mpra/many_sims_mprascore/', sim_id, '_out.txt')

  mprascore_cmd = paste0('mprascore ',
                         oligo_out_path, ' ',
                         bc_out_path, ' ',
                         '-RNAcols:', paste0('RNA_', 1:6, collapse = ','), ' ',
                         '-DNAcols:DNA_1,DNA_2,DNA_3 ',
                         out_path, ' ',
                         '-p:1000')

  # run command

  system(mprascore_cmd)

  return('donesk')
}

load('/mnt/bigData2/andrew/MPRA/sim_mpra/many_sims/sim_grid.RData')

sim_grid %>%
  mutate(ms_res = parallel::mclapply(sim_id,
                                     mprascore_sim,
                                     mc.cores = 16,
                                     mc.preschedule = FALSE))
```

The code block below evaluates the performance of MPRAscore on each simulation.

```
library(pROC)

assess_ms_output = function(sim_id){
  id_outputs = read_tsv(paste0('/mnt/bigData2/andrew/MPRA/sim_mpra/many_sims_mprascore/', sim_id, '_out
  load(paste0('/mnt/bigData2/andrew/MPRA/sim_mpra/many_sims/', sim_id, '.RData'))

  current_sim %<>% left_join(id_outputs, by = c('variant_id' = 'RSID'))

  if (any(current_sim$truly_functional)){
    roc_obj = roc(response = current_sim$truly_functional,
                  predictor = current_sim$p_perm)

    auc_val = auc(roc_obj)
    pr_obj = pROC::coords(roc_obj,
                          ret = c('recall', 'precision'),
                          transpose = FALSE)

    # Compute AUPR using the trapezoidal rule
    pr_mat = pr_obj %>% .[nrow(.):1,] %>% .[!duplicated(.$recall),]
```

```
    nrow_pr = nrow(pr_mat)
    drecall = pr_mat[2:nrow_pr,1] - pr_mat[1:(nrow_pr - 1),1]
    avg_precision = (pr_mat[2:nrow_pr, 2] + pr_mat[1:(nrow_pr - 1), 2]) / 2
    avg_precision[1] = pr_mat[2,2] # the first precision value is returned as NaN -- set to next value
    daupr = drecall * avg_precision
    aupr = sum(daupr)

  } else{
    auc_val = NA
    aupr = NA
  }

  typical_table = current_sim %>%
    mutate(fdr_hit = q_perm < .05) %>%
    select(truly_functional, fdr_hit) %>%
    table

  nonzero_cor = current_sim %>%
    filter(truly_functional) %$%
    cor(effect_size, score)

  spread_at_zero = current_sim %>%
    filter(!truly_functional) %>%
    pull(score) %>%
    sd

  res_df = tibble(auc = auc_val,
                  aupr = aupr,
                  typical_table = list(typical_table),
                  spread_at_zero = spread_at_zero,
                  nonzero_cor = nonzero_cor)
}

load('/mnt/bigData2/andrew/MPRA/sim_mpra/many_sims/sim_grid.RData')

ms_performance = sim_grid %>%
  mutate(perf_table = pbmcapply::pbmclapply(sim_id,
                                            assess_ms_output,
                                            mc.cores = 16))

save(ms_performance,
     file = '~/dev_malacoda/outputs/mprascore/ms_performance.RData')
```

4.5 Run QuASAR-MPRA on simulations

```
library(QuASAR)
library(tidyverse)
library(pROC)
library(magrittr)
load('/mnt/bigData2/andrew/MPRA/sim_mpra/many_sims/sim_grid.RData')

fit_and_assess_qm = function(sim_id){
```

```r
load(paste0('/mnt/bigData2/andrew/MPRA/sim_mpra/many_sims/', sim_id, '.RData'))

unnested = current_sim %>%
  select(-variant_id) %>%
  unnest(c(variant_data))

qm_input = unnested %>%
  select(variant_id, allele, matches('[DR]NA', ignore.case = FALSE)) %>%
  gather(sample_id, count, -(1:2)) %>%
  group_by(variant_id) %>%
  summarise(ref_rna = sum(count[grepl('RNA', sample_id) & allele == 'ref']),
            alt_rna = sum(count[grepl('RNA', sample_id) & allele != 'ref']),
            dna_prop = sum(count[grepl('DNA', sample_id) & allele == 'ref']) / sum(count[grepl('DNA',

qm_sim = fitQuasarMpra(qm_input$ref_rna,
                       qm_input$alt_rna,
                       qm_input$dna_prop) %>%
  bind_cols(qm_input %>% select(variant_id))

save(qm_sim,
     file = paste0('/mnt/bigData2/andrew/MPRA/sim_mpra/many_sims_qm/', sim_id, '.RData'))

res_df = current_sim %>% left_join(qm_sim, by = 'variant_id') %>%
  mutate(betas.beta.binom = -betas.beta.binom) # QM uses an unintuitive directionality

if (any(current_sim$truly_functional)){
  roc_obj = roc(predictor = res_df$pval3,
                response = res_df$truly_functional)

  auc_val = auc(roc_obj)

  pr_obj = pROC::coords(roc_obj,
                        ret = c('recall', 'precision'),
                        transpose = FALSE)

  # Compute AUPR using the trapezoidal rule
  pr_mat = pr_obj %>% .[nrow(.):1,] %>% .[!duplicated(.$recall),]
  nrow_pr = nrow(pr_mat)
  drecall = pr_mat[2:nrow_pr,1] - pr_mat[1:(nrow_pr - 1),1]
  avg_precision = (pr_mat[2:nrow_pr, 2] + pr_mat[1:(nrow_pr - 1), 2]) / 2
  avg_precision[1] = pr_mat[2,2] # the first precision value is returned as NaN -- set to next value
  daupr = drecall * avg_precision
  aupr = sum(daupr)

  save(roc_obj, aupr, auc_val,
       file = paste0('/mnt/bigData2/andrew/MPRA/sim_mpra/many_sims_qm/', sim_id, '_roc.RData'))

  nonzero_cor = res_df %>%
    filter(truly_functional) %$%
    cor(betas.beta.binom, effect_size)

  spread_at_0 = res_df %>%
    filter(!truly_functional) %>%
```

```r
      pull(betas.beta.binom) %>%
      sd

    save(nonzero_cor, spread_at_0,
         file = paste0('/mnt/bigData2/andrew/MPRA/sim_mpra/many_sims_qm/', sim_id, '_es.RData'))

    perf_df = tibble(spread_at_0 = spread_at_0,
                     auc = auc_val,
                     nonzero_cor = nonzero_cor,
                     aupr = aupr)

    save(perf_df,
         file = paste0('/mnt/bigData2/andrew/MPRA/sim_mpra/many_sims_qm/', sim_id, '_perf_df.RData'))
  } else {

    spread_at_0 = res_df %>%
      filter(!truly_functional) %>%
      pull(betas.beta.binom) %>%
      sd

    perf_df = tibble(spread_at_0 = spread_at_0,
                     auc = as.numeric(NA),
                     nonzero_cor = as.numeric(NA),
                     aupr = as.numeric(NA))
    save(perf_df,
         file = paste0('/mnt/bigData2/andrew/MPRA/sim_mpra/many_sims_qm/', sim_id, '_perf_df.RData'))
  }

  return('donesk :)')

}

cl = parallel::makeCluster(20, type = "FORK")
pbapply::pblapply(sim_grid$sim_id,
                  fit_and_assess_qm,
                  cl = cl)

get_qm_perf = function(sim_id){
  load(paste0('/mnt/bigData2/andrew/MPRA/sim_mpra/many_sims_qm/', sim_id, '_perf_df.RData'))
  return(perf_df)
}

qm_perf = sim_grid %>%
  mutate(perf_dfs = map(sim_id, get_qm_perf)) %>%
  unnest_legacy() %>%
  group_by(p_func, n_variants, n_bpa) %>%
  summarise(med_saz = median(`spread_at_0`),
            med_nzc = median(nonzero_cor),
            med_auc = median(auc),
            med_aupr = median(aupr)) %>%
  ungroup %>%
  gather(metric, value, matches('med_')) %>%
  mutate(method = 'QuASAR-MPRA')
```

```
save(qm_perf,
     file = '/home/andrew/dev_malacoda/outputs/qm_perf.RData')
```

4.6 Run MPRAnalyze on simulations

```
library(tidyverse)
library(MPRAnalyze)
library(pROC)

load('/mnt/bigData2/andrew/MPRA/sim_mpra/many_sims/sim_grid.RData')

run_ma = function(sim_id){

  load(paste0('/mnt/bigData2/andrew/MPRA/sim_mpra/many_sims/', sim_id, '.RData'))

  current_sim %<>% select(-variant_id) %>% unnest(c(variant_data))

  dna_mat = current_sim %>%
    group_by(variant_id) %>%
    mutate(barcode_i = 1:n()) %>%
    ungroup %>%
    select(variant_id, barcode_i, allele, matches('DNA', ignore.case = FALSE)) %>%
    gather(sample_id, counts, matches('DNA')) %>%
    unite(bsa, barcode_i, sample_id, allele, sep = ';') %>%
    spread(bsa, counts) %>%
    column_to_rownames('variant_id') %>%
    as.matrix

  dna_col_annot = dna_mat %>%
    colnames %>% str_split(';', simplify = TRUE) %>%
    as.data.frame() %>%
    mutate_all(factor) %>%
    set_names(c('barcode', 'batch', 'allele'))

  rna_mat = current_sim %>%
    group_by(variant_id) %>%
    mutate(barcode_i = 1:n()) %>%
    ungroup %>%
    select(variant_id, barcode_i, allele, matches('RNA', ignore.case = FALSE)) %>%
    gather(sample_id, counts, matches('RNA')) %>%
    unite(bsa, barcode_i, sample_id, allele, sep = ';') %>%
    spread(bsa, counts) %>%
    column_to_rownames('variant_id') %>%
    as.matrix

  rna_col_annot = rna_mat %>%
    colnames %>% str_split(';', simplify = TRUE) %>%
    as.data.frame() %>%
    mutate_all(factor) %>%
    set_names(c('barcode', 'batch', 'allele'))
```

```r
obj = MpraObject(dnaCounts = dna_mat, rnaCounts = rna_mat,
                 dnaAnnot = dna_col_annot, rnaAnnot = rna_col_annot)

obj = estimateDepthFactors(obj , lib.factor = 'batch',  which.lib = 'both')
                             rnaDesign = ~ allele )
obj = analyzeComparative(obj = obj,
                         dnaDesign = ~barcode,
                         rnaDesign = ~allele ,
                         reducedDesign = ~ 1)

res <- testLrt(obj) %>%
  rownames_to_column('variant_id')

perf_df = current_sim  %>% select(variant_id, truly_functional, effect_size) %>% unique %>%
  left_join(res)

if (any(current_sim$truly_functional)){

  roc_obj = roc(predictor = perf_df$pval,
                response = perf_df$truly_functional, quiet = TRUE)
  auc_val = auc(roc_obj)

  pr_obj = pROC::coords(roc_obj,
                        ret = c('recall', 'precision'),
                        transpose = FALSE)

  # Compute AUPR using the trapezoidal rule
  pr_mat = pr_obj %>% .[nrow(.):1,] %>% .[!duplicated(.$recall),]
  nrow_pr = nrow(pr_mat)
  drecall = pr_mat[2:nrow_pr,1] - pr_mat[1:(nrow_pr - 1),1]
  avg_precision = (pr_mat[2:nrow_pr, 2] + pr_mat[1:(nrow_pr - 1), 2]) / 2
  avg_precision[1] = pr_mat[2,2] # the first precision value is returned as NaN -- set to next value
  daupr = drecall * avg_precision
  aupr = sum(daupr)

  res_df = perf_df %>%
    summarise(saz = sd(logFC[effect_size == 0]),
              nzc = cor(effect_size[effect_size != 0],
                        -logFC[effect_size != 0]),
              auc = auc_val,
              aupr = aupr)
} else{
  res_df = perf_df %>%
    summarise(saz = sd(logFC[effect_size == 0]),
              aupr = NA,
              auc = NA,
              nzc = NA)

}

save(obj, res, res_df,
     file = paste0('/mnt/bigData2/andrew/MPRA/sim_mpra/many_sims_mpranalyze/', sim_id, '.RData'))
return('donesk')
```

```r
}

ids_to_run = sim_grid %>%
  group_by(p_func, n_variants, n_bpa) %>%
  mutate(g_id = 1:n()) %>%
  ungroup %>%
  sample_frac() %>%
  arrange(g_id) %>%
  pull(sim_id)

 # This was stopped after 5 days of run time. Each simulation grid point had
 # finished at least 26 simulated assays.
ma_res = parallel::mclapply(ids_to_run,
                            run_ma,
                            mc.cores = 20)

ma_done = list.files('/mnt/bigData2/andrew/MPRA/sim_mpra/many_sims_mpranalyze', pattern = '.RData') %>%
  str_replace('.RData', '')

get_ma_res = function(sim_id){
  load(paste0('/mnt/bigData2/andrew/MPRA/sim_mpra/many_sims_mpranalyze/', sim_id, '.RData'))
  return(res_df)
}

ma_res = sim_grid %>%
  filter((sim_id %in% ma_done)) %>%
  mutate(sim_res = map(sim_id, get_ma_res))

ma_perf = ma_res$sim_res %>%
  bind_rows %>%
  bind_cols(ma_res) %>%
  group_by(p_func, n_variants, n_bpa) %>%
  summarise(med_saz = median(saz),
            med_aupr = median(aupr),
            med_auc = median(auc),
            med_nzc = median(nzc)) %>%
  ungroup %>%
  mutate(method = 'MPRAnalyze') %>%
  gather(metric, value, matches('med_'))

save(ma_perf,
     file = '/home/andrew/dev_malacoda/outputs/ma_perf.RData')
```

5 Compare all methods

We compare the performance on the simulated assays by median performance across four metrics:

- AUC - The area under the receiver-operating curve. Higher is better.
- AUPR - The area under the precision-recall curve. Higher is better.
- spread at zero - The standard deviation of TS estimates of the truly nonfunctional variants. Lower is better.
- nonzero correlation - The correlation between the true TS values and the TS estimates. Higher is better.

The first two metrics quantify the binary classification properties of the various methods, while the latter two metrics quantify the accuracy of the TS estimates themselves. The latter two metrics can be visualized in terms of figure 3A from the paper, reproduced here:

```
sim_id = 'zwnxvebu'

load(paste0('/mnt/bigData2/andrew/MPRA/sim_mpra/many_sims/', sim_id, '.RData'))
load(paste0('/mnt/bigData2/andrew/MPRA/sim_mpra/many_sims_malacoda/', sim_id, '/analysis_res.RData'))

sysfonts::font_add(family = 'Arial',
                   regular = '~/my_fonts/arial.ttf',
                                     italic = '~/my_fonts/Arial Italic.ttf')

plot_data =  left_join(current_sim, analysis_res,
                       by = 'variant_id')

example_comparison_plot = plot_data %>%
  ggplot(aes(effect_size, ts_post_mean)) +
  geom_point(size = .5) +
  geom_abline(lty = 2,
              color = 'grey40') +
  geom_segment(aes(x = 0, xend = 0,
                   y = -3.1, yend = -1.9),
               arrow = arrow(length = unit(2, 'mm'))) +
  annotate('text',
           x = 0, y = -3.5, label = '90%, 95%, or 100% of variants',
           size = 6) +
  geom_boxplot(fill = rgb(0,0,0,0),
               data = plot_data %>% filter(effect_size == 0),
               outlier.shape = NA,
               color = 'grey60') +
  labs(x = 'Simulated Transcription Shift',
       y = 'Estimated\nTranscription Shift') +
  theme_light()

example_comparison_plot
```

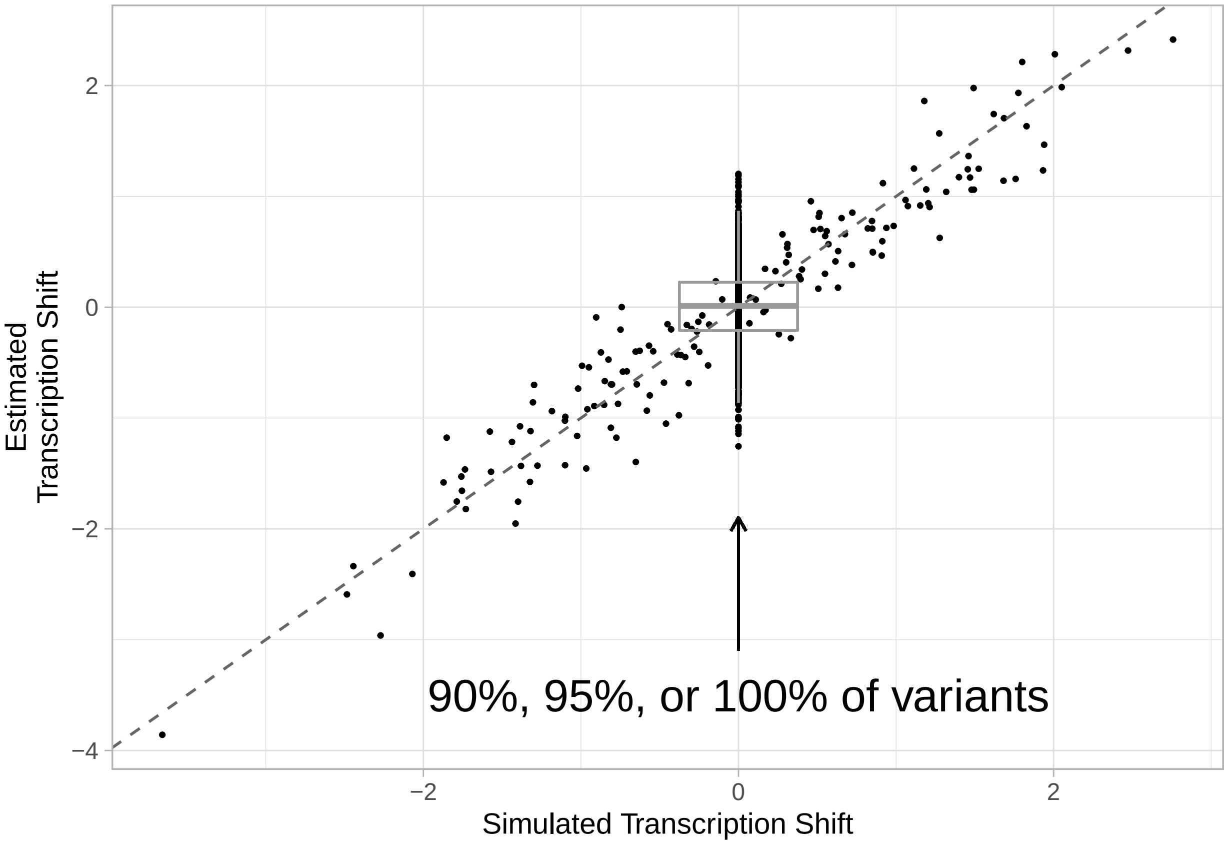

The cluster of points located at x = 0 are truly non-functional variants. An ideal analysis would shrink their TS estimates to zero. Thus, the "spread at zero" defined above can be used to quantify the accuracy of estimates on non-functional variants. The "nonzero correlation" measures the accuracy of estimates for the complement set of variants. The points located off the vertical line at x = 0 are truly functional variants. A better model will produce estimates that correlate more highly with the true values for a higher "nonzero correlation".

The code block below joins all of the performance metrics for each method and generates Figure 3 from the paper.

```
load('/mnt/bigData2/andrew/MPRA/sim_mpra/many_sims/sim_grid.RData')
load('/home/andrew/dev_malacoda/outputs/mprascore/ms_performance.RData')
load('/mnt/bigData2/andrew/MPRA/sim_mpra/mpralm_sim_performance.RData')
load('/mnt/bigData2/andrew/MPRA/sim_mpra/t_performance.RData')
load('/home/andrew/dev_malacoda/outputs/malacoda_perf.RData')
load('/home/andrew/dev_malacoda/outputs/ma_perf.RData')
load('/home/andrew/dev_malacoda/outputs/qm_perf.RData')

malacoda_perf %<>%
  left_join(sim_grid, by = 'sim_id') %>%
  group_by(p_func, n_variants, n_bpa) %>%
  summarise(med_auc = median(auc),
            med_saz = median(spread_at_zero),
            med_nzc = median(nonzero_cor),
            med_aupr = median(aupr)) %>%
  ungroup %>%
  mutate(method = 'malacoda') %>%
  gather(metric, value, matches('med_'))
```

```r
ms_performance %<>%
  unnest_legacy() %>%
  group_by(p_func, n_variants, n_bpa) %>%
  summarise(med_auc = median(auc),
            med_saz = median(spread_at_zero),
            med_nzc = median(nonzero_cor),
            med_aupr = median(aupr)) %>%
  ungroup %>%
  mutate(method = 'MPRAscore') %>%
  gather(metric, value, matches('med_'))

mpralm_sim_performance %<>%
  group_by(p_func, n_variants, n_bpa) %>%
  summarise(med_auc = median(auc),
            med_saz = median(`spread_at_0`),
            med_nzc = median(nonzero_cor),
            med_aupr = median(aupr)) %>%
  ungroup %>%
  mutate(method = 'mpralm') %>%
  gather(metric, value, matches('med_'))

t_perf %<>%
  group_by(p_func, n_variants, n_bpa) %>%
  summarise(med_auc = median(auc),
            med_saz = median(mean_saz),
            med_nzc = median(mean_nzc),
            med_aupr = median(aupr)) %>%
  ungroup %>%
  mutate(method = 't-test') %>%
  gather(metric, value, matches('med_'))

all_perf = bind_rows(malacoda_perf,
                     ms_performance,
                     mpralm_sim_performance,
                     t_perf,
                     ma_perf,
                     qm_perf)

save(all_perf,
     file = '/home/andrew/dev_malacoda/outputs/all_perf.RData')

color_map = c('malacoda' = '#aa0000',
              'mpralm' = '#4daf4a',
              't-test' = '#984ea3',
              'MPRAscore' = '#377eb8',
              'MPRAnalyze' = '#FF7F00',
              'QuASAR-MPRA' = '#F781BF')

sysfonts::font_add(family = 'Arial',
                   regular = '~/my_fonts/arial.ttf',
                                     italic = '~/my_fonts/Arial Italic.ttf')

all_perf_fig = all_perf %>%
```

```
  mutate(method = factor(method, levels = c('malacoda', 'MPRAnalyze', 'mpralm', 'QuASAR-MPRA', 't-test'
  filter(n_variants == 3000, p_func == .05, n_bpa == 10) %>%
  mutate(metric = str_replace_all(metric,
                                  c('med_auc' = 'median AUC',
                                    'med_saz' = 'median spread\nat zero',
                                    'med_nzc' = 'median nonzero\ncorrelation',
                                    'med_aupr' = 'median AUPR'))) %>%
  ggplot(aes(method, value, group = method)) +
  geom_col(aes(fill = method),
                      show.legend = FALSE) +
  scale_fill_manual(breaks = names(color_map),
                    values = color_map) +
  facet_grid(~metric) +
  labs(x = NULL) +
  theme_light(base_family = 'Arial') +
  theme(strip.text = element_text(color = 'black'),
        axis.text.x = element_text(angle = 45, hjust = 1),
        axis.title.y = element_blank(),
        text = element_text(family = 'Arial',
                            size = 40,
                            lineheight = .3))

ggsave(all_perf_fig,
      filename = '/home/andrew/dev_malacoda/outputs/figures/sim_comparison.png',
       dpi = 300,
      height = 3.5,
      units = 'in',
      width = 5.5)
```

The code block below generates the supplementary figures analogous to Figure 3 for the other grid points in the simulation. In cases where p_func = 0, the AUC, AUPR, and nonzero correlation values are not defined, so these panels are left blank.

```
load('/home/andrew/dev_malacoda/outputs/all_perf.RData')

library(magrittr)
library(tidyverse)

color_map = c('malacoda' = '#aa0000',
              'mpralm' = '#4daf4a',
              't-test' = '#984ea3',
              'MPRAscore' = '#377eb8',
              'MPRAnalyze' = '#FF7F00',
              'QuASAR-MPRA' = '#F781BF')

sysfonts::font_add(family = 'Arial',
                   regular = '~/my_fonts/arial.ttf',
                                    italic = '~/my_fonts/Arial Italic.ttf')

make_bar_plot = function(n_variants_plot, p_func_plot){

  b_plot = all_perf %>%
    mutate(method = factor(method, levels = c('malacoda', 'MPRAnalyze', 'mpralm', 'QuASAR-MPRA', 't-tes
    filter(n_variants_plot == n_variants, p_func == p_func_plot) %>%
```

```r
    mutate(metric = str_replace_all(metric,
                                    c('med_auc' = 'median AUC',
                                      'med_saz' = 'median spread at zero',
                                      'med_nzc' = 'median nonzero correlation',
                                      'med_aupr' = 'median AUPR')),
           n_bpa = paste('barcodes per allele = ', n_bpa)) %>%
    ggplot(aes(method, value, group = method)) +
    geom_col(aes(fill = method),
             show.legend = FALSE) +
    scale_fill_manual(breaks = names(color_map),
                      values = color_map) +
    facet_grid(n_bpa~metric) +
    labs(x = NULL) +
    theme_light() +
    theme(strip.text = element_text(color = 'black'),
          axis.text.x = element_text(angle = 45, hjust = 1),
          axis.title.y = element_blank(),
          text = element_text(family = 'Arial',
                              size = 24, lineheight = .3)) +
    labs(title = paste0('n_variants = ', n_variants_plot, ', p_func = ', p_func_plot))

  ggsave(b_plot,
         filename = paste0('/home/andrew/dev_malacoda/outputs/figures/b_plot_', n_variants_plot, '_', p[illegible]
         height = 3.5,
         units = 'in',
         width = 5.5)
}

## Command to make the plots:
# all_perf %>% select(1:2) %>%
#   arrange(-p_func) %>%
#   unique %>%
#   {parallel::mcmapply(make_bar_plot,
#                       .$n_variants, .$p_func,
#                       mc.cores = 3)}

image_list = all_perf %>% select(1:2) %>%
  arrange(-p_func) %>%
  unique %$%
  map2_chr(n_variants, p_func,
      ~paste0('/home/andrew/dev_malacoda/outputs/figures/b_plot_', .x, '_', .y, '.png'))

knitr::include_graphics(image_list, auto_pdf = TRUE)
```

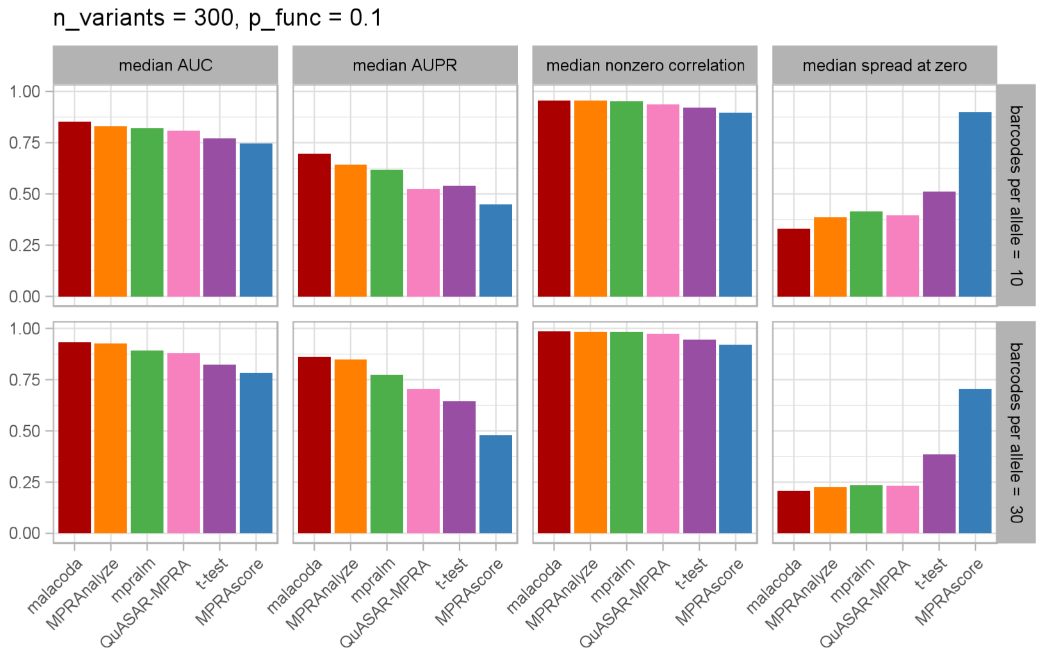
n_variants = 300, p_func = 0.1
median AUC
median AUPR
median nonzero correlation
median spread at zero
barcodes per allele = 10
barcodes per allele = 30
1.00
0.75
0.50
0.25
0.00
malacoda
MPRAnalyze
mpralm
QuASAR-MPRA
t-test
MPRAscore

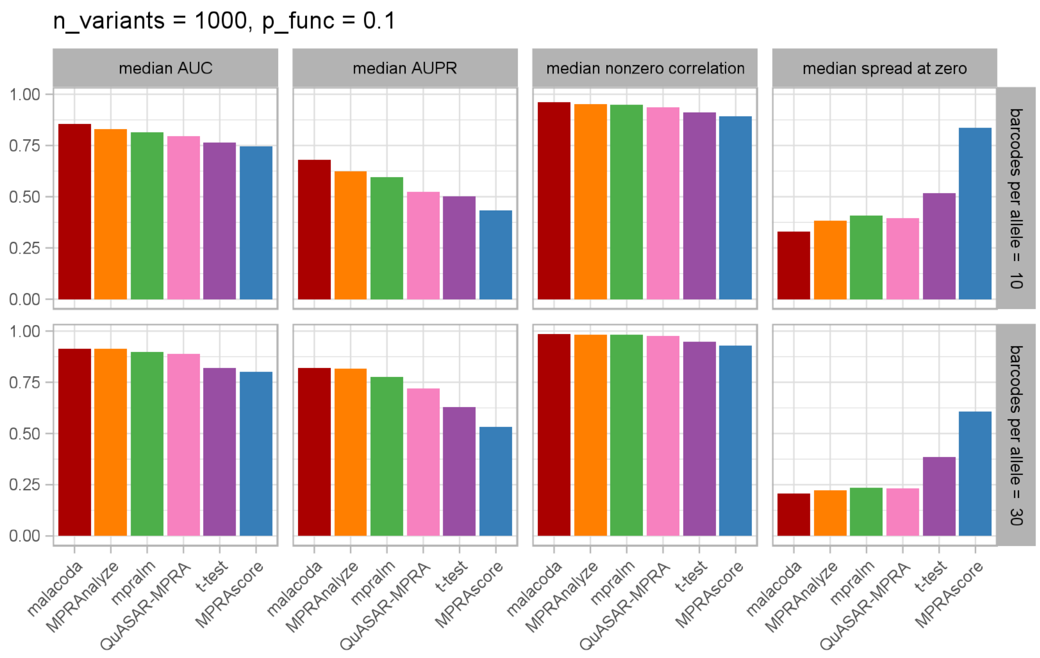
n_variants = 1000, p_func = 0.1
median AUC
median AUPR
median nonzero correlation
median spread at zero
barcodes per allele = 10
barcodes per allele = 30
1.00
0.75
0.50
0.25
0.00
malacoda
MPRAnalyze
mpralm
QuASAR-MPRA
t-test
MPRAscore

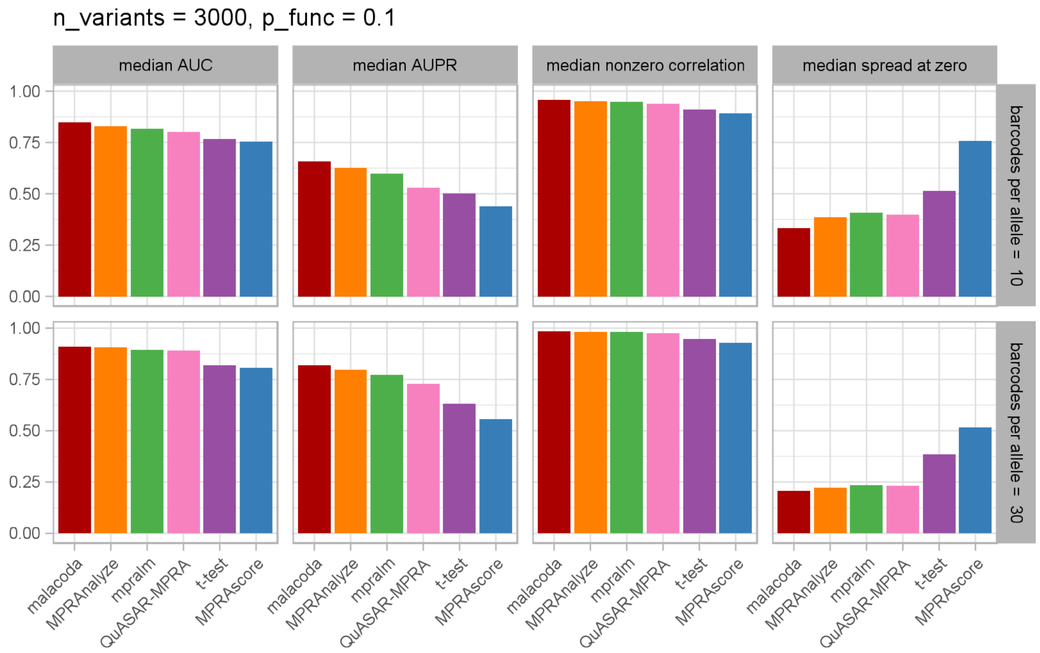
n_variants = 3000, p_func = 0.1
median AUC
median AUPR
median nonzero correlation
median spread at zero
barcodes per allele = 10
barcodes per allele = 30
0.00
0.25
0.50
0.75
1.00
malacoda
MPRAnalyze
mpralm
QuASAR-MPRA
t-test
MPRAscore

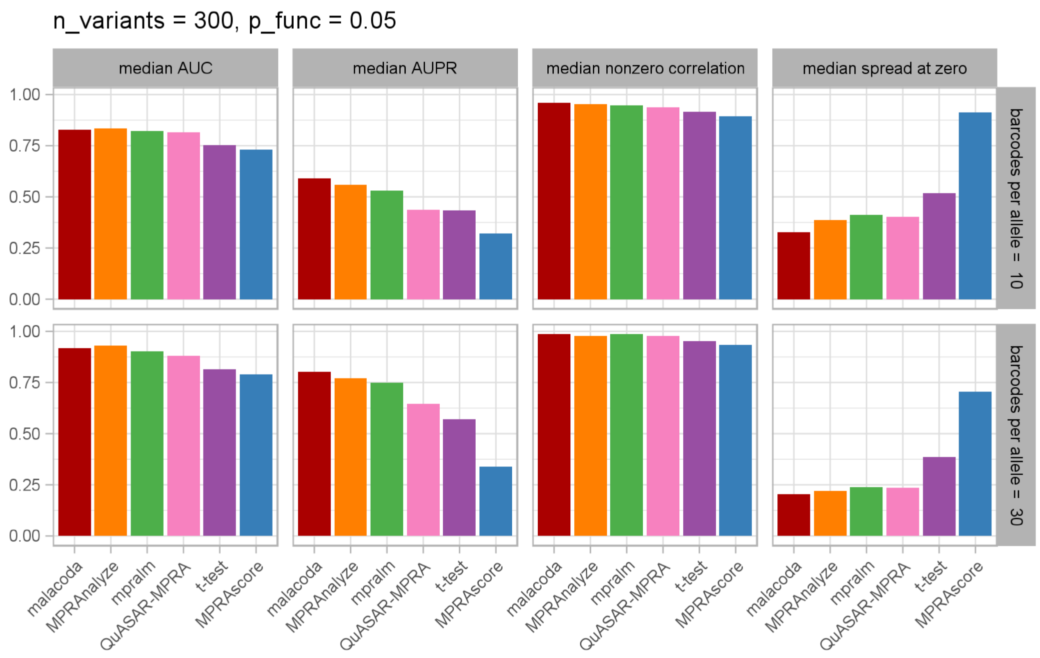
n_variants = 300, p_func = 0.05
median AUC
median AUPR
median nonzero correlation
median spread at zero
barcodes per allele = 10
barcodes per allele = 30
0.00
0.25
0.50
0.75
1.00
malacoda
MPRAnalyze
mpralm
QuASAR-MPRA
t-test
MPRAscore

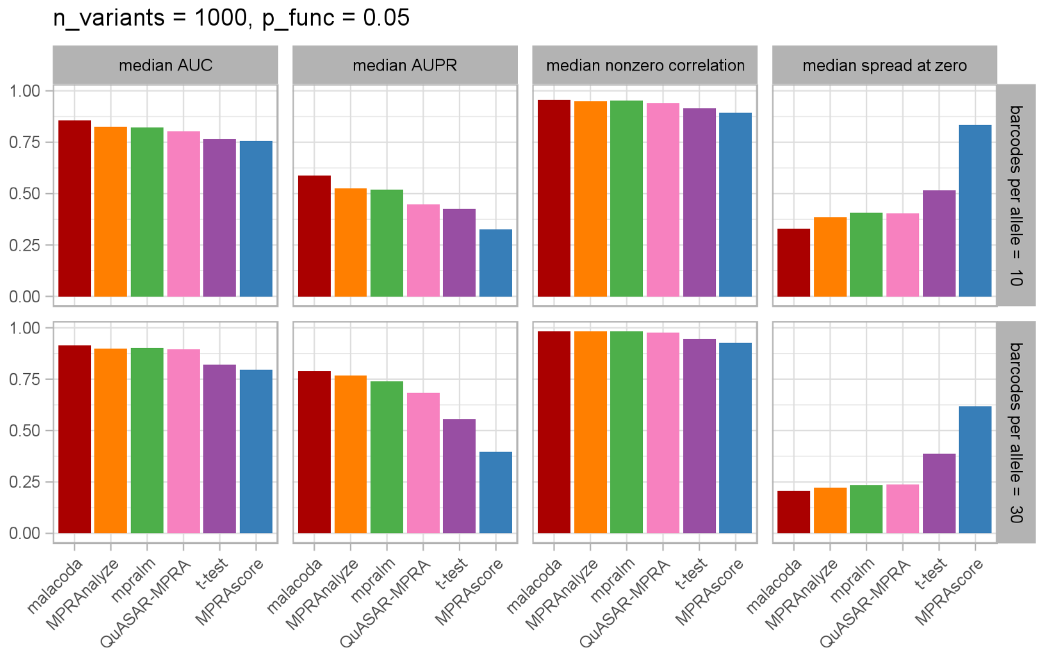
n_variants = 1000, p_func = 0.05
median AUC
median AUPR
median nonzero correlation
median spread at zero
barcodes per allele = 10
barcodes per allele = 30
0.00
0.25
0.50
0.75
1.00
malacoda
MPRAnalyze
mpralm
QuASAR-MPRA
t-test
MPRAscore

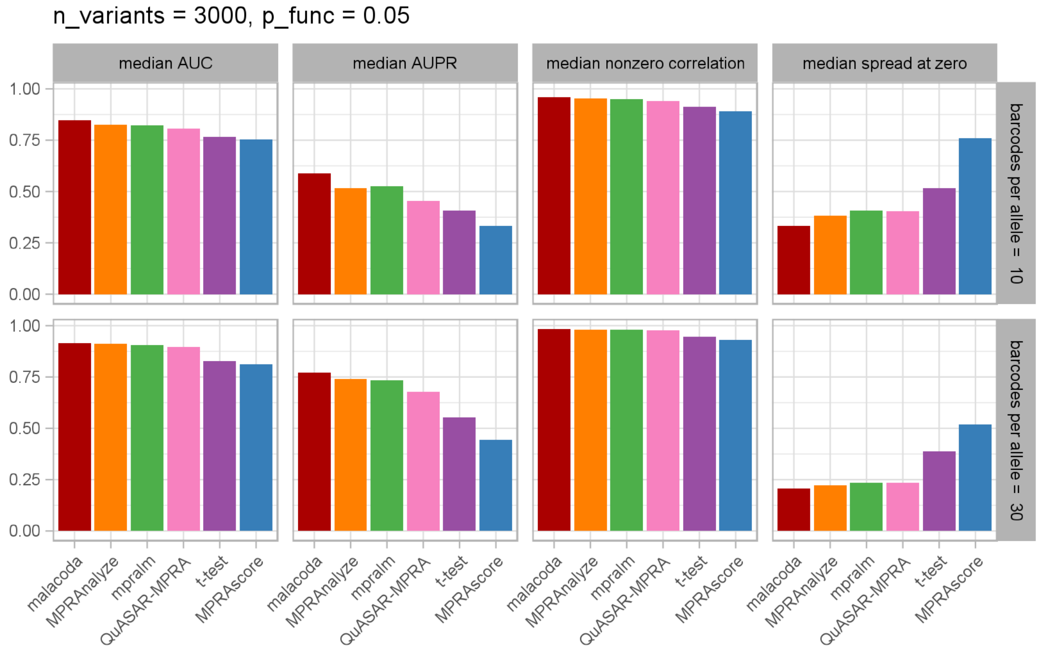
n_variants = 3000, p_func = 0.05
median AUC
median AUPR
median nonzero correlation
median spread at zero
barcodes per allele = 10
barcodes per allele = 30
0.00
0.25
0.50
0.75
1.00
malacoda
MPRAnalyze
mpralm
QuASAR-MPRA
t-test
MPRAscore

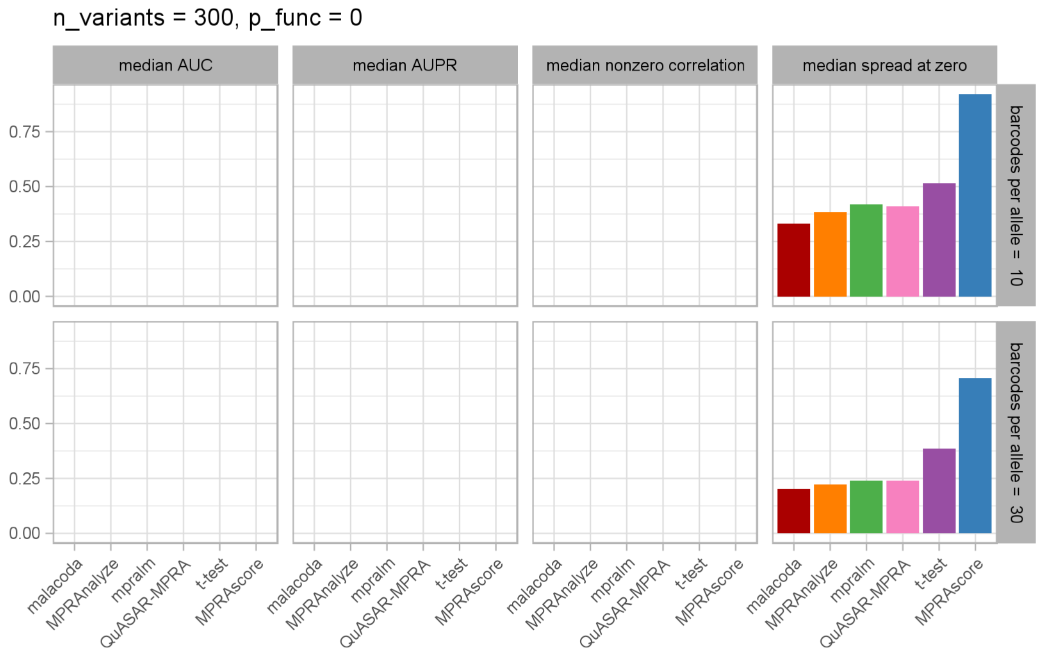
n_variants = 300, p_func = 0
median AUC
median AUPR
median nonzero correlation
median spread at zero
barcodes per allele = 10
barcodes per allele = 30
0.00
0.25
0.50
0.75
malacoda
MPRAnalyze
mpralm
QuASAR-MPRA
t-test
MPRAscore

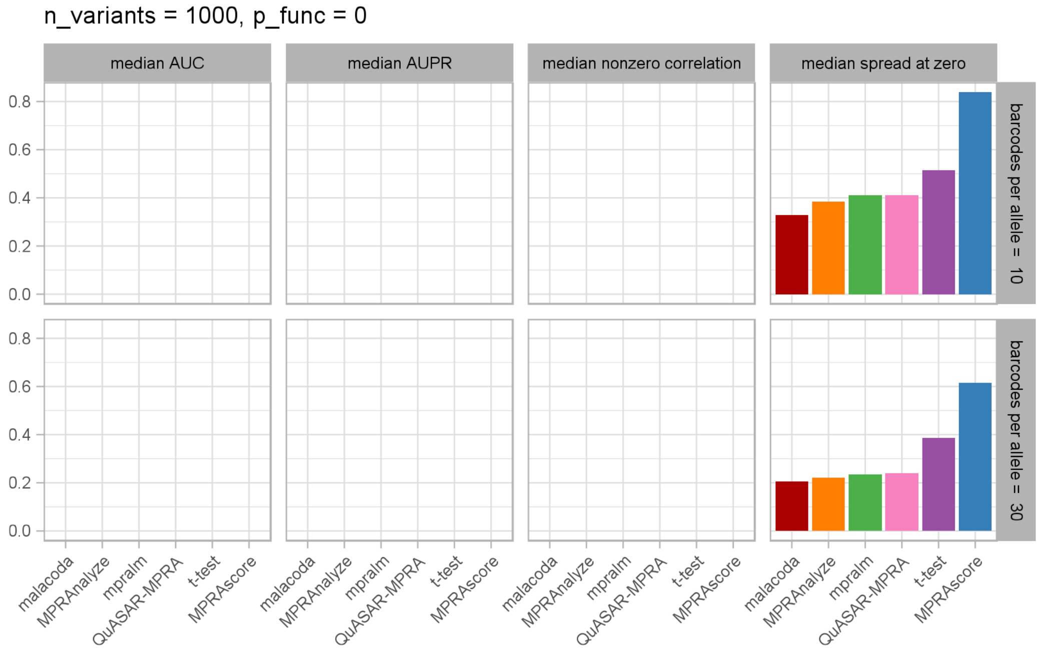
n_variants = 1000, p_func = 0
median AUC
median AUPR
median nonzero correlation
median spread at zero
barcodes per allele = 10
barcodes per allele = 30
0.0
0.2
0.4
0.6
0.8
malacoda
MPRAnalyze
mpralm
QuASAR-MPRA
t-test
MPRAscore

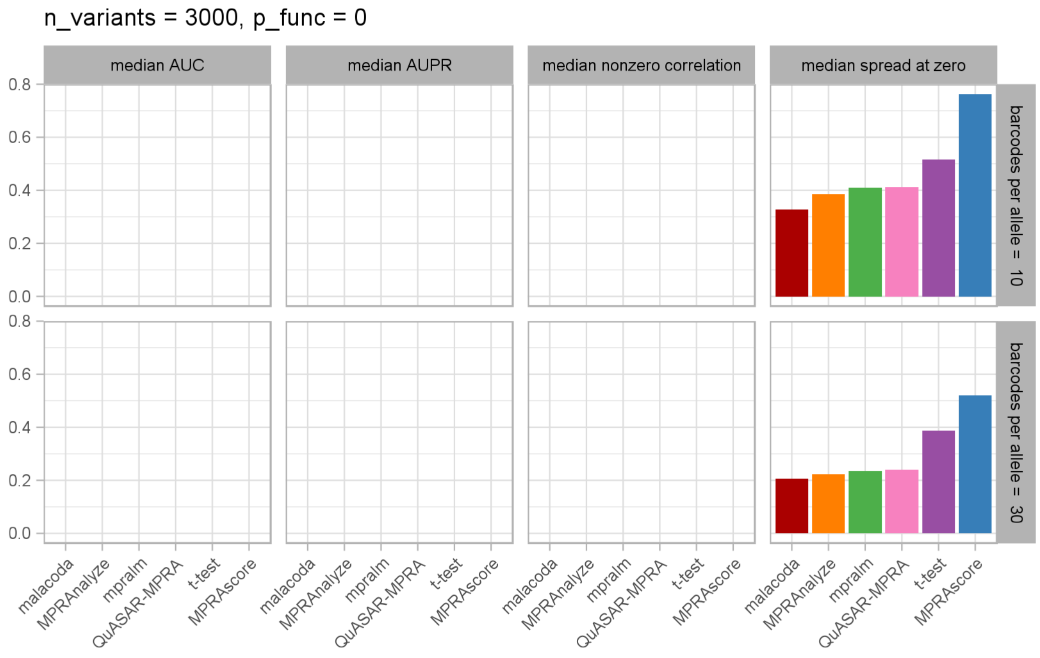

malacoda performs the best by each metric in all but a few cases where the differences are small and within the range of the simulation's Monte Carlo error.

6 malacoda regularization

MPRA simulations also present an opportunity to demonstrate the regularizing effect of the prior used by malacoda. Using the data from an assay with 5% functional variants out of 3000 and 30 barcodes per allele, we can plot the (unregularized) t-test estimates against the regularized malacoda estimates:

```
library(tidyverse)
library(magrittr)
library(ghzutils)

load('/mnt/bigData2/andrew/MPRA/sim_mpra/many_sims/sim_grid.RData')
load('/mnt/bigData2/andrew/MPRA/sim_mpra/many_sims_t/dtsrpkio_test_df.RData')
load('/mnt/bigData2/andrew/MPRA/sim_mpra/many_sims_malacoda/dtsrpkio/analysis_res.RData')


plot_data = test_df %>%
  left_join(analysis_res, suffix = c('_t', '_malacoda'), by = 'variant_id') %>%
  add_local_density(ts_estimate, ts_post_mean)

reg_plot = plot_data %>%
  ggplot(aes(ts_estimate, ts_post_mean)) +
  geom_point(aes(color = local_density)) +
  geom_abline(lty = 2) +
  stat_ellipse(data = plot_data %>% filter(abs(ts_estimate) < 1), color = 'red') +
  geom_curve(aes(x = .77, xend = .82,
                 y = .77, yend = .2),
             curvature = -.4,
             color = 'red',
```

```r
              arrow = arrow(length = unit(.02, 'npc'))) +
  geom_curve(aes(x = -.77, xend = -.82,
                 y = -.77, yend = -.2),
              curvature = -.5,
              color = 'red',
              arrow = arrow(length = unit(.02, 'npc'))) +
  theme_light() +
  scale_color_viridis_c(guide = FALSE) +
  labs(x = 'Unregularized estimates (t-test)',
       y = 'malacoda estimate',
       title = 'Simulated MPRA results demonstrate the\nregularizing effect of the prior') +
  theme(text = element_text(size = 15))
ggsave(reg_plot, filename = '~/dev_malacoda/outputs/regularization_plot.png')
```

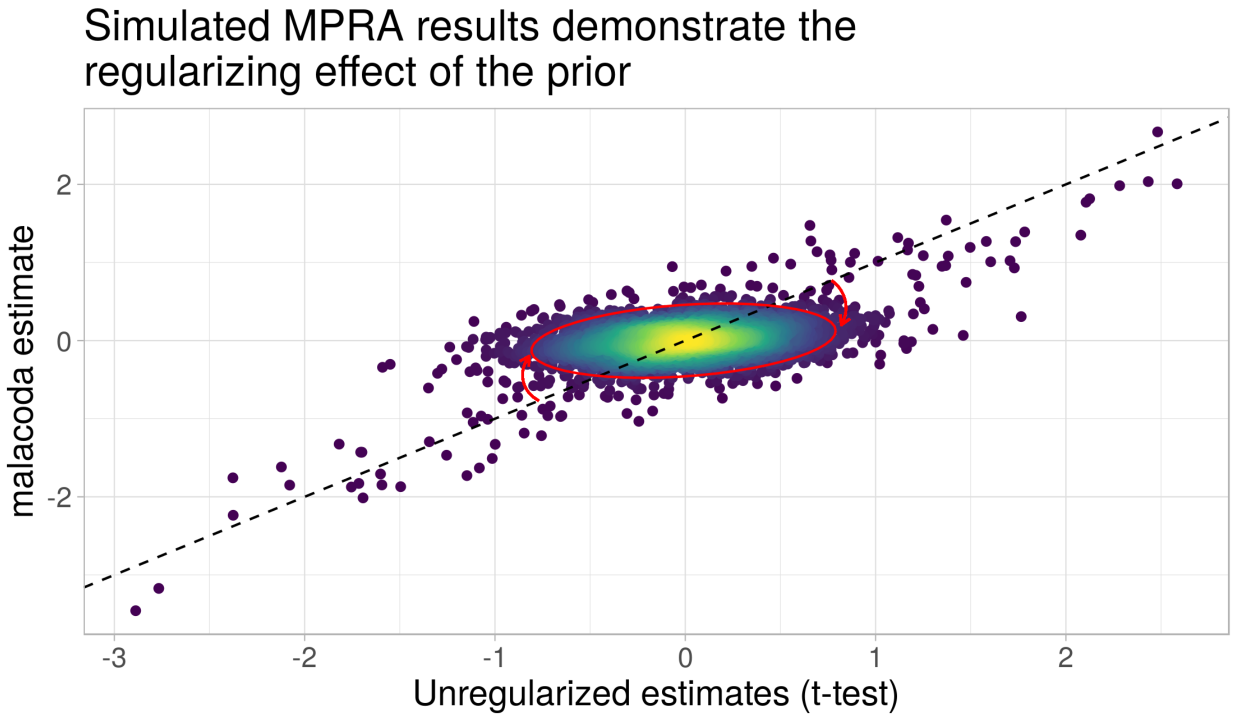

Because the empirical prior concentrates probability mass around TS = 0, posterior estimates of TS are shrunken towards 0 as well. This is indicated by the less than 45° angle of the red ellipse, containing the majority of non-functional variants. If the malacoda model did not impose regularization, this ellipse would tend to align with dotted line on y=x. The uncommon cases of malacoda estimates more extreme than the unregularized t-test estimates are the result of different model structures producing different estimates.

Nonetheless, any binarization process will result in false positives. malacoda users can mitigate the risk of false positives by increasing the TS HDI threshold used in `fit_mpra_model()` to 99% (from 95%). Alternatively, the function `malacoda::increase_regularization()` can be used to manually increase the regularization used by malacoda by a set factor. Both of these measures come at the cost of an increased false negative rate.

7 Conclusion

Using simulations we systematically compare the performance of the malacoda method for MPRA analysis against three alternative methods: t-test, mpralm and MPRAscore. The results demonstrate that malacoda

is superior to these alternatives under a variety of parameter contexts by several performance metrics: AUC, spread at zero, and correlation of estimates with true simulated transcriptional shift values.

8 Session Info

```
devtools::session_info()
```

```
## - Session info ----------------------------------------------------------
##  setting  value
##  version  R version 3.5.1 (2018-07-02)
##  os       Ubuntu 14.04.3 LTS
##  system   x86_64, linux-gnu
##  ui       X11
##  language (EN)
##  collate  en_US.UTF-8
##  ctype    en_US.UTF-8
##  tz       America/Chicago
##  date     2020-05-27
##
## - Packages --------------------------------------------------------------
##  package     * version date       lib source
##  assertthat    0.2.1   2019-03-21 [2] CRAN (R 3.5.1)
##  backports     1.1.4   2019-04-10 [1] CRAN (R 3.5.1)
##  broom         0.5.2   2019-04-07 [1] CRAN (R 3.5.1)
##  callr         3.3.1   2019-07-18 [1] CRAN (R 3.5.1)
##  cellranger    1.1.0   2016-07-27 [2] CRAN (R 3.5.1)
##  cli           1.1.0   2019-03-19 [2] CRAN (R 3.5.1)
##  colorspace    1.4-1   2019-03-18 [2] CRAN (R 3.5.1)
##  crayon        1.3.4   2017-09-16 [2] CRAN (R 3.5.1)
##  desc          1.2.0   2018-05-01 [2] CRAN (R 3.5.1)
##  devtools      2.0.1   2018-10-26 [2] CRAN (R 3.5.1)
##  digest        0.6.20  2019-07-04 [1] CRAN (R 3.5.1)
##  dplyr       * 0.8.3   2019-07-04 [2] CRAN (R 3.5.1)
##  ellipsis      0.2.0.1 2019-07-02 [1] CRAN (R 3.5.1)
##  evaluate      0.13    2019-02-12 [2] CRAN (R 3.5.1)
##  forcats     * 0.4.0   2019-02-17 [2] CRAN (R 3.5.1)
##  fs            1.2.7   2019-03-19 [2] CRAN (R 3.5.1)
##  generics      0.0.2   2018-11-29 [2] CRAN (R 3.5.1)
##  ggplot2     * 3.2.1   2019-08-10 [1] CRAN (R 3.5.1)
##  glue          1.3.1   2019-03-12 [2] CRAN (R 3.5.1)
##  gtable        0.3.0   2019-03-25 [1] CRAN (R 3.5.1)
##  gtools        3.8.1   2018-06-26 [1] CRAN (R 3.5.1)
##  haven         2.1.0   2019-02-19 [2] CRAN (R 3.5.1)
##  highr         0.8     2019-03-20 [2] CRAN (R 3.5.1)
##  hms           0.5.0   2019-07-09 [1] CRAN (R 3.5.1)
##  htmltools     0.3.6   2017-04-28 [2] CRAN (R 3.5.1)
##  httr          1.4.0   2018-12-11 [2] CRAN (R 3.5.1)
##  jsonlite      1.6     2018-12-07 [2] CRAN (R 3.5.1)
##  kableExtra    1.1.0   2019-03-16 [1] CRAN (R 3.5.1)
##  knitr         1.22    2019-03-08 [2] CRAN (R 3.5.1)
##  labeling      0.3     2014-08-23 [2] CRAN (R 3.5.1)
##  lattice       0.20-38 2018-11-04 [2] CRAN (R 3.5.1)
```

```
##  lazyeval      0.2.2   2019-03-15 [2] CRAN (R 3.5.1)
##  lifecycle     0.1.0   2019-08-01 [1] CRAN (R 3.5.1)
##  lubridate     1.7.4   2018-04-11 [2] CRAN (R 3.5.1)
##  magrittr    * 1.5     2014-11-22 [2] CRAN (R 3.5.1)
##  memoise       1.1.0   2017-04-21 [2] CRAN (R 3.5.1)
##  modelr        0.1.4   2019-02-18 [2] CRAN (R 3.5.1)
##  munsell       0.5.0   2018-06-12 [2] CRAN (R 3.5.1)
##  nlme          3.1-137 2018-04-07 [2] CRAN (R 3.5.1)
##  pillar        1.4.2   2019-06-29 [1] CRAN (R 3.5.1)
##  pkgbuild      1.0.4   2019-08-05 [1] CRAN (R 3.5.1)
##  pkgconfig     2.0.2   2018-08-16 [2] CRAN (R 3.5.1)
##  pkgload       1.0.2   2018-10-29 [2] CRAN (R 3.5.1)
##  plyr          1.8.4   2016-06-08 [2] CRAN (R 3.5.1)
##  prettyunits   1.0.2   2015-07-13 [2] CRAN (R 3.5.1)
##  processx      3.4.1   2019-07-18 [1] CRAN (R 3.5.1)
##  ps            1.3.0   2018-12-21 [2] CRAN (R 3.5.1)
##  purrr       * 0.3.2   2019-03-15 [2] CRAN (R 3.5.1)
##  R6            2.4.0   2019-02-14 [2] CRAN (R 3.5.1)
##  Rcpp          1.0.1   2019-03-17 [2] CRAN (R 3.5.1)
##  readr       * 1.3.1   2018-12-21 [2] CRAN (R 3.5.1)
##  readxl        1.3.1   2019-03-13 [2] CRAN (R 3.5.1)
##  remotes       2.0.2   2018-10-30 [2] CRAN (R 3.5.1)
##  reshape2      1.4.3   2017-12-11 [2] CRAN (R 3.5.1)
##  rlang         0.4.0   2019-06-25 [1] CRAN (R 3.5.1)
##  rmarkdown     1.12    2019-03-14 [2] CRAN (R 3.5.1)
##  rprojroot     1.3-2   2018-01-03 [2] CRAN (R 3.5.1)
##  rstudioapi    0.10    2019-03-19 [2] CRAN (R 3.5.1)
##  rvest         0.3.2   2016-06-17 [2] CRAN (R 3.5.1)
##  scales        1.0.0   2018-08-09 [2] CRAN (R 3.5.1)
##  sessioninfo   1.1.1   2018-11-05 [2] CRAN (R 3.5.1)
##  stringi       1.4.3   2019-03-12 [2] CRAN (R 3.5.1)
##  stringr     * 1.4.0   2019-02-10 [2] CRAN (R 3.5.1)
##  sysfonts      0.8     2018-10-11 [1] CRAN (R 3.5.1)
##  tibble      * 2.1.3   2019-06-06 [1] CRAN (R 3.5.1)
##  tidyr       * 1.0.0   2019-09-11 [2] CRAN (R 3.5.1)
##  tidyselect    0.2.5   2018-10-11 [2] CRAN (R 3.5.1)
##  tidyverse   * 1.2.1   2017-11-14 [2] CRAN (R 3.5.1)
##  usethis       1.4.0   2018-08-14 [2] CRAN (R 3.5.1)
##  vctrs         0.2.0   2019-07-05 [1] CRAN (R 3.5.1)
##  viridisLite   0.3.0   2018-02-01 [2] CRAN (R 3.5.1)
##  webshot       0.5.1   2018-09-28 [1] CRAN (R 3.5.1)
##  withr         2.1.2   2018-03-15 [2] CRAN (R 3.5.1)
##  xfun          0.5     2019-02-20 [2] CRAN (R 3.5.1)
##  xml2          1.2.0   2018-01-24 [2] CRAN (R 3.5.1)
##  yaml          2.2.0   2018-07-25 [2] CRAN (R 3.5.1)
##  zeallot       0.1.0   2018-01-28 [1] CRAN (R 3.5.1)
##
## [1] /home/andrew/R/x86_64-pc-linux-gnu-library/3.5
## [2] /usr/local/src/R-3.5.1/library
```
