# Supplementary figures and images for: Bayesian modelling of high-throughput sequencing assays with malacoda
Source: PLoS Comput Biol. 2020 Jul 21;16(7):e1007504. doi: 10.1371/journal.pcbi.1007504 (PMC7394446; doi:10.1371/journal.pcbi.1007504)

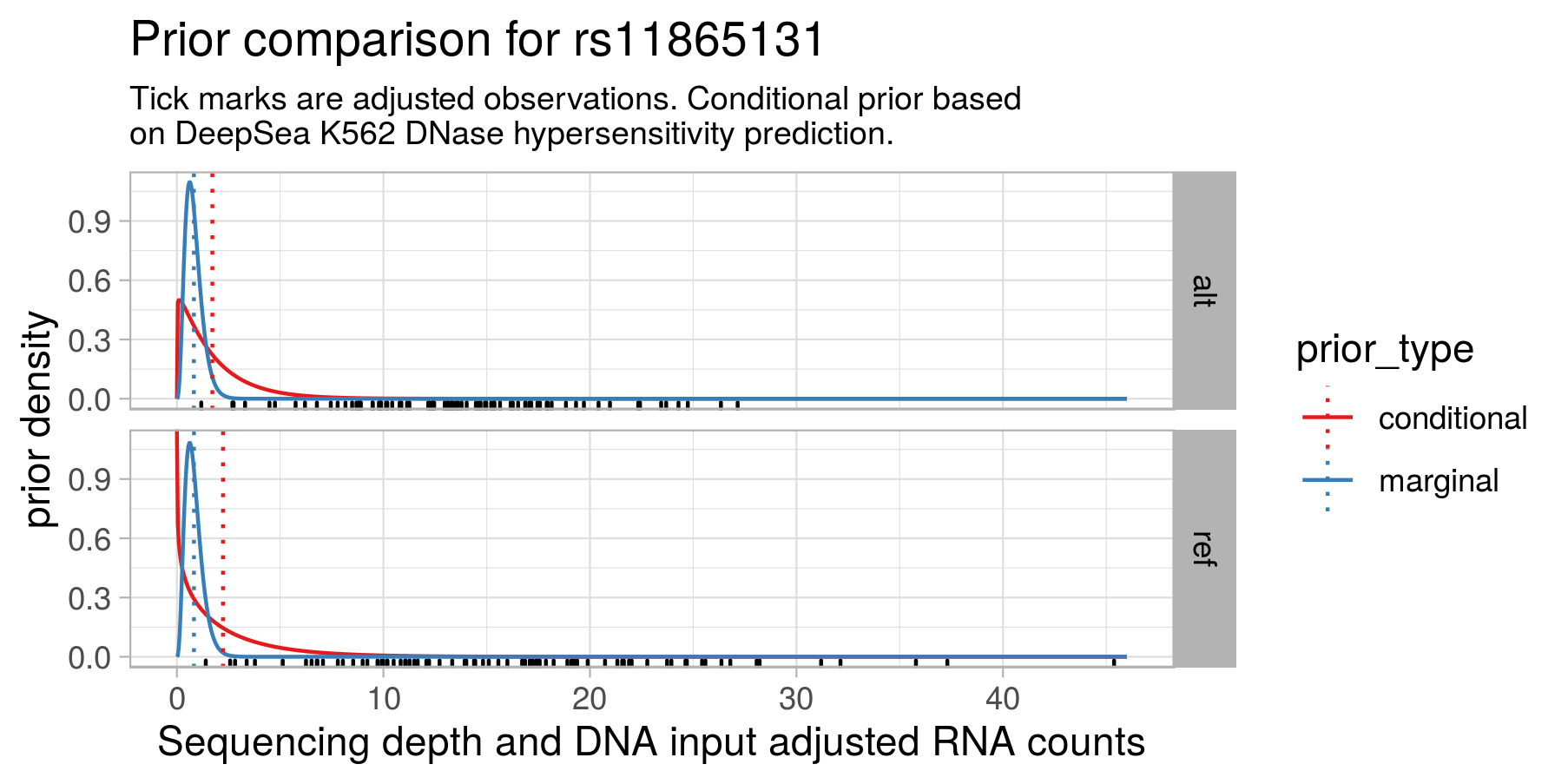

Supplement: S1 Fig — This figure compares the allelic priors for the RNA activity for both alleles of rs11865131. The blue line shows the marginal prior, the red line the conditional prior based on the DeepSea K562 DNase hypersensitivity prediction. Dotted lines show the prior means. Black tick marks show the RNA count observations adjusted for sequencing depth and DNA input. Because this variant tended to show higher than usual activity in both alleles, both priors shrink the activity considerably. Notably however, the conditional prior shrinks less than the marginal, particularly in the reference allele. The allele-specific difference in shrinkage is what allowed the conditional prior-based analysis to identify this variant as functional. (TIF) [file pcbi.1007504.s006.tif]

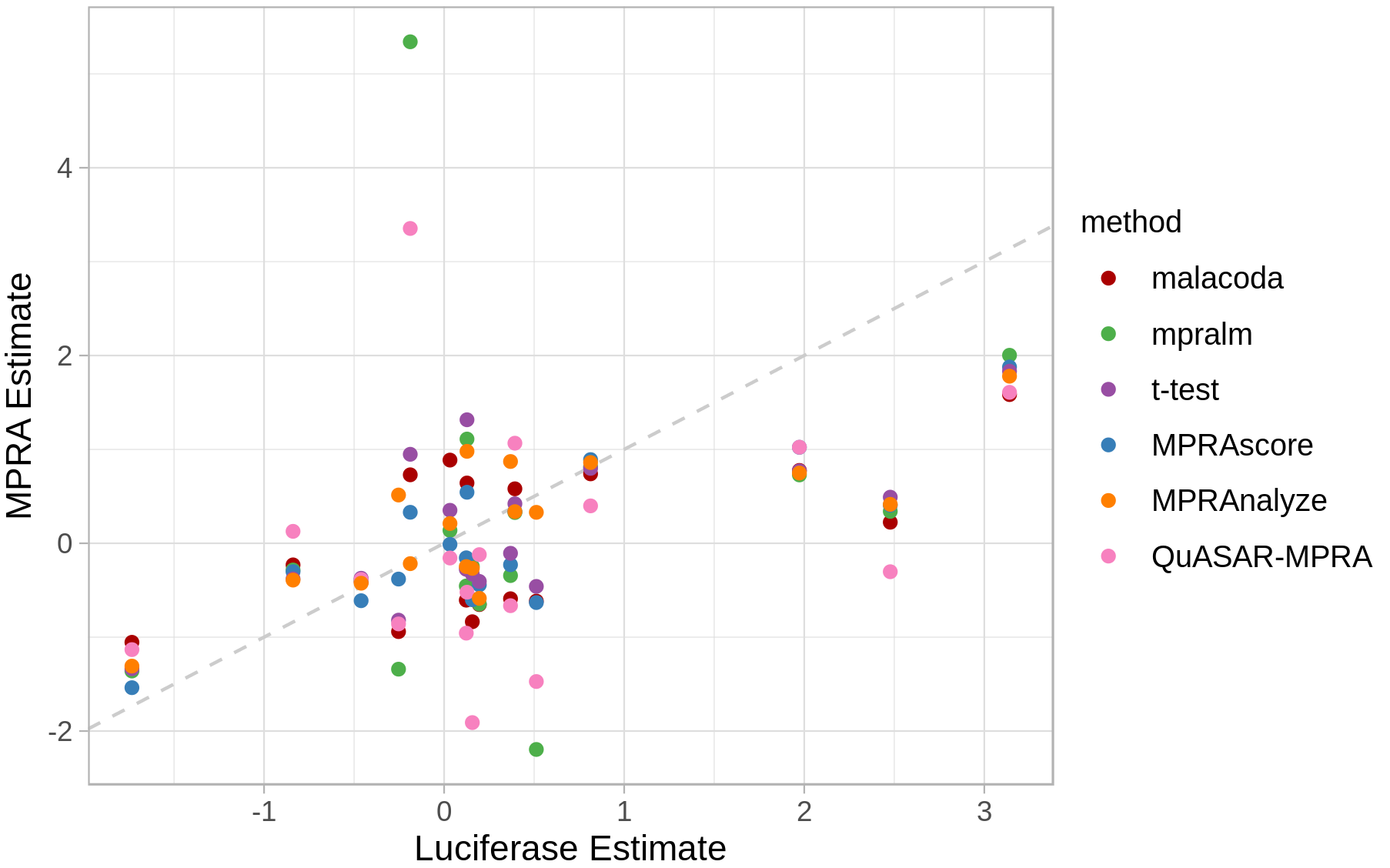

Supplement: S2 Fig — A scatterplot demonstrates the relationship between luciferase-based estimates of TS against MPRA-based estimates from each MPRA analysis method. (TIF) [file pcbi.1007504.s007.tif]
