# Supplementary material for: Bayesian modelling of high-throughput sequencing assays with malacoda
Source: PLoS Comput Biol. 2020 Jul 21;16(7):e1007504. doi: 10.1371/journal.pcbi.1007504 (PMC7394446; doi:10.1371/journal.pcbi.1007504)
Supplement: Attachment — Submitted filename: malacoda_resubmission_letter.docx [file pcbi.1007504.s008.docx]

Dear Editor,

Thank you for the detailed and thoughtful reviews of our manuscript “Bayesian modelling of high-throughput sequencing assays with malacoda” (PCOMPBIOL-D-19-01801R1). The reviewers found our manuscript made important contributions to the field, but they asked many substantive questions and raised concerns for us to address. We carefully considered each of the questions as well as the editor and reviewer comments, and we have substantially refined and added to the manuscript to address all of the points that were raised.

As pointed out by one of the reviewers, perhaps the most remarkable contribution of malacoda is its statistical model for high-throughput NGS screen data. This model is important because it presents a careful and well substantiated consideration of the statistical characteristics and dispersion properties in this important class of biological data. We have shown how alternative methods have incompletely addressed these issues. As computational and statistical scientists this methodologic contribution is both satisfying and an important contribution to advance the field of high throughput NGS screens -- not just MPRA, but also CRISPR screens and other assays -- including those currently in use and those that will come in the future.

Key elements we have added to this major revision are:

1) New supplemental documentation that details the malacoda model and that provides all step by step code elements in the malacoda implementation -- code that is also freely available on Github.
2) A new supplement that presents the computational details of the Bayesian techniques employed by malacoda to determine results. These details were requested by the reviewers and substantiate that malacoda is convergent and performing as expected.
3) Additional wet bench validation data demonstrating that the new biological discovery for rs11865131 made with malacoda is recapitulated in wet bench experiments.
4) Additional simulation data demonstrating malacoda’s performance against four alternative methods in a variety of metrics.
5) All raw data, including the barcode counts of our primary MPRA data, as used in the malacoda evaluation are now available in the S5 Dataset, as required by the journal and as requested by the reviewers.

We again thank the reviewers for their thoughtful review of our work.

Regards,

Andrew Ghazi, Xianguo Kong, Ed Chen, Leonard Edelstein, and Chad Shaw

“Bayesian modelling of high-throughput sequencing assays with malacoda.”

PCOMPBIOL-D-19-01801R1

Response to Reviewers

In our resubmission some document elements have changed names. These changes are displayed in a table below for clarity.

| Initial submission | Resubmission |
| --- | --- |
| Figure 3D | Figure 5A |
| S1 Appendix | S2 Appendix |
| S2 Appendix | S3 Appendix |
| S3 Dataset | S4 Dataset |
| S4 Dataset | S5 Dataset |

Detailed responses to each question and concern are presented below. We have left the original review comments indented once and our response to each point unindented immediately afterwards.

Reviewer #1: Summary:

This manuscript presents malacoda, a Bayesian method for identifying alleles with significantly different abilities to activate gene expression in an MPRA. malacoda accounts for variation due to unknown DNA concentrations and allows for users to incorporate prior information about variants, properties that existing methods do not have. In addition, unlike previous methods, it does not summarize the data before modeling. The manuscript reports results from simulations that show that malacoda is able to detect functional variants with higher accuracy than previous methods across multiple numbers of bar-codes per allele, array sizes, and fractions of functional variants. The manuscript also shows that malacoda tends to agree more with other methods on real MPRA data than the other methods agree with each other. In addition, the manuscript presents luciferase assays that malacoda’s results for sixteen variants, including multiple functional variants that were not identified as functional by other methods. Overall, I think that malacoda is a useful tool for using MPRA data to determine if non-coding variants are functional that is likely to be widely used because it is more accurate than existing methods and a thorough description of how it works as well as code needed to run it have been made publicly available.

Major Comments:

1. Figure 3A makes me concerned that malacoda’s precision is not very high because a large percentage of the simulated variants with transcription shifts greater than zero seem to be non-functional. Figure 3D enhances this concern because many of the luciferase estimates near zero seem to correspond to MPRA estimates that are further from zero. The manuscript would be more convincing if the authors could show that malacoda had high precision or at least substantially higher precision than other methods. In addition, since Figures 3A and 3D do suggest that malacoda does not report any non-functional variants to have transcription shift > 1.5, including the precision at different transcription shift cutoffs might also be helpful.

We have added area under precision-recall (AUPR) panels to the updated simulation results. Because malacoda is based on MCMC and some simulated variants had pseudo-pvalues of exactly zero, we used a conservative linear approximation in order to estimate the extremely low p-values for these high-signal variants. This was necessary in order to ensure a well-defined AUPR metric. There are no transcription shift cutoffs used to define malacoda’s classification results. The procedure we used is presented in detail in section four of the updated S3 Appendix. The results show that malacoda outperforms the alternative methods in terms of AUPR in all of the simulation scenarios we tested. For clarity, we have now reorganized Figure 3 to present only simulation results and moved all bench data results to Figure 5 to avoid confusion.

2. The paper mentions primary MPRA data, but this data is never described in detail. If the data is new to this paper, then a description of the data, including how the tested sequences were selected, what MPRA protocol was used, and how the plasmids were constructed, needs to be added. If the data was taken from another publication, then that publication needs to be cited.

We have updated S5 Dataset to include the full set of barcode counts with anonymized variant IDs from our primary dataset; information concerning the MPRA assay performed is also presented in the simulation and validation studies section. The counts will enable readers to replicate and compare results or extend the MPRA analysis described in this work. The focus of our manuscript is on our statistical method and software; we do not intend to present the biological results of our primary MPRA study in this manuscript, although we do present a new biological discovery overlooked by different methods in a previously published MPRA report.

Minor Comments:

Introduction:

1. The value of malacoda is not that it is Bayesian but that it does not have the drawbacks of previous methods – ignoring variation due to unknown DNA concentrations, summarizing the data prior to modeling, and not accounting for relevant prior information. I would re-structure the final paragraph of the introduction to emphasize its strengths over existing methods instead of its Bayesian nature.

We agree with the reviewer that the malacoda statistical model is perhaps the most important and enduring contribution of this work. We have adjusted the language in the last paragraph of the introduction on lines 113-119 to shift emphasis to the practical implications of the model as suggested.

Methods:

1. In line 144, the second dot should be a comma.

This has been fixed.

2. In lines 144-145, the reasons behind the definitions of the means for the negative binomial distribution were clear to me, but I am not sure if they would be clear to someone who has not thought about MPRAs before. It therefore might be useful to add a sentence explaining the reasons.

We have now added thorough description in the model text and S1 Appendix to provide a more detailed description of the model.

3. The definition of “95% highest density interval on TS” in line 171 was not clear to me; it would be great if this could be clarified.

We have added a component to figure 2B that illustrates the concept. We have also added clarifications for determining p-value-like quantities based on our HDI interval estimates so that we can more easily compare our results with the alternative methods as shown in Figure 3.

4. The reason that the method for learning the gamma priors removes the need for post-hoc multiple testing correction, as claimed in line 193, was not clear to me. My understanding is that, even though all of the variants in the MPRA are used to learn the gamma priors, the effect of each variant on transcription is still tested separately, which would mean that multiple testing correction should still be required.

We would first like to emphasize that Figure 3 demonstrates the superior performance of malacoda’s empirical prior approach with respect to the risk of false positives. Without any *post hoc* multiple testing correction malacoda displays superior binary classification performance. To address the reviewer’s concern, we have altered the language in our manuscript to more accurately present the regularizing effect of a prior distribution as an alternative to *post hoc* multiple testing correction. We have also added a section to the simulation supplement that demonstrates this effect. The diagram included below illustrates the concept behind a regularizing prior. A binary decision to accept or reject the null hypothesis can be viewed as checking whether or not the 95% confidence interval includes zero. In circumstances involving multiple testing, the interval is artificially widened to control either the family-wise error rate (the Bonferroni correction) or the false discovery rate (the Benjamini-Hochberg procedure) while leaving the estimate of the effect size (the central dot) intact. In contrast to this, regularizing priors shrink the estimate toward the prior mean, i.e. zero, while leaving the interval width largely unchanged. This has the added benefit of moderating the reported effect sizes.

The precise magnitude of the interval widening and estimate shrinkage as shown above depends on the details of the p-value adjustment method and/or prior in use. However in our demonstrative example above the adjusted confidence interval fails to reject the null hypothesis while the regularized posterior interval does not include zero as a credible value. Users of malacoda can use the functions sample_from_prior(), plot_prior_samples(), and increase_regularization() to ensure that the estimated empirical priors match their prior beliefs about the distribution of potential transcription shifts in their assay.

5. If an existing software package was used for model fitting, then the package and settings that were used should be added.

We have added a brief description of the computational procedures to the methods section of the manuscript. We have also added the components of the probability model describing the distributions for the means and dispersions. The newly added S1 Appendix describes the model fitting procedure in detail. This supplement presents concise descriptions of the the computational algorithms underlying our method (such as the No-U-Turn Sampler), the code used to run these algorithms, and the diagnostic methods used to ensure the algorithms converge.

6. In the section on Simulation and Validation Studies, it would be great if a complete list the parameters used in the simulations could be provided (providing them as a supplemental table would be sufficient). If the supplemental websites contain every parameter used in the simulations, then directing the reader to the appropriate supplemental website should be sufficient.

We have added a bulleted list of the statistical parameters used in the Simulation and Validation Studies to sections 2 and 3 of S3 Appendix. The code used to generate the simulated assays is included in S3 appendix, so we have also added a sentence in the main text pointing this out.

7. It would be great if the authors could also compare area under the precision-recall curve because there are probably more non-functional variants than functional variants, so high sensitivity and specificity does not guarantee high precision.

We have now extended the S3 Appendix to evaluate the AUPR among the other performance metrics. We have updated Figure 3C with this information. As detailed in the updated manuscript and appendix, malacoda outperforms the alternatives we tested by AUPR in addition to the other three metrics (AUC, correlation, and standard deviation of null variants).

8. In the section on Experimental Procedures, it would be helpful to add a description of the full experimental procedure for the new MPRA in this manuscript.

We have added a brief description of the experimental conditions used in our primary dataset to line 253-255.

9. In the section on Experimental Procedures, it would be helpful to direct readers to the supplemental file containing the variants tested in luciferase assays.

We have added a sentence to the end of the experimental methods section on line 377 pointing out these data.

Results:

1. At the beginning of the section on Biological Results, it would be helpful to include an explanation of why the number of luciferase reporter assays was not enough to overcome the amount of noise inherent to light intensity-based measurements or provide a citation of a paper that explains this.

We have updated the language in this section starting at line 459 to emphasize that the variants chosen for luciferase testing were predominantly selected from borderline variants. Several of the variants turned out non-functional in the luciferase assays, so this subset of variants contaminated with non-functional findings lacks the true positives necessary to assess the relative performance of the analysis methods. We have added content to the results section to clarify this point.

2. Lines 294-296 state that the results from the luciferase assay are consistent with the MPRA estimates, but this seems to occur for only the largest transcription shifts. I think that this should be modified to say that the results are consistent for large transcription shifts.

We have adapted the language in the manuscript to reflect this.

3. Figure 4 suggests that evaluations were done using DeepSea-based priors, but there is no description of these results other than that the effect of rs11865131 was found using the DeepSea based priors. Figure 4B suggests that the results using the DeepSea-based priors were highly correlated with those without the priors. It would be helpful to add a summary of these results to the Results section.

We have added a sentence starting on line 429 that briefly explains the strong correlation between the marginal and conditional prior fits. In short, the two fits agree for most variants because DeepSea only produces strong predictions for a minority of variants. The majority of variants in the Ulirsch dataset have DeepSea prediction score close to zero, in which case the conditional prior tends to approach the marginal. Any predictor that could strongly change the result from the marginal prior fit would be a cause for concern. Such a scenario would imply that the predictor contains an amount of information comparable to what is coming from the assay itself. As with any analysis using a prior distribution, it’s important to ensure that the prior does not unduly influence the posterior. The empirical approach we utilize ensures that our prior distributions are reasonable in the context of each assay.

4. The results provide evidence that rs11865131 is functional because it affects the activity of an intronic enhancer. The results about this variant would be more compelling if the authors added that this variant is not close to any exon-intron boundary, so it is unlikely to also affect splicing.

We have added a sentence to the end of the biological results section on line 497 detailing this point. Additionally we have performed a new luciferase assay demonstrating the transcriptional functionality of rs11865131.

5. It would be helpful to add a description of how the analysis of rs11865131 overlap with ChIP-seq peaks was done (For example, were all peaks or only reproducible peaks used? When there were datasets from multiple labs for a TF or histone modification, which dataset was used?) and what the TFs and histone modifications are whose peaks overlap it.

The ChIP-seq analysis was performed with HaploReg, a non-coding variant annotation tool. It uses data from the ENCODE project consortium, and in the case of rs11865131 includes experiments in K562 cells from several labs under multiple treatments. The URL below contains the detailed information in this database for this variant:

https://pubs.broadinstitute.org/mammals/haploreg/detail_v4.1.php?query=&id=rs11865131

6. If a luciferase assay was done for rs11865131, it would be helpful to add a description of the assay’s results in the Results section in addition to having them in a supplemental file.

We performed a luciferase assay of rs11865131 and confirmed its functionality. This result is now presented in figure 5B. These results have also been added to the table of luciferase results given in S4 Dataset.

Discussion:

1. The paper would be easier to follow if the description of how the models were fit was moved to the Methods section.

We have moved the initial description of the model fitting process with Stan into the Software subsection in the Methods section starting around line 329. We also have added an overview of the default fitting parameters used by our package.

2. The phrase “seemingly worthwhile” in line 334 should be defined.

We have removed this ambiguous phrasing and refined the wording to clarify that the variational first pass defaults to a check using an 80% posterior interval.

3. Line 342 mentions “an additional layer of hyper-parameters.” If these hyper-parameters were used to obtain the results in this paper, then a description of them should be added to the Methods section. If not, then it would be helpful to clarify that they are an option that the user can add but were not used to obtain this paper’s results.

We have clarified that the fully hierarchical model was not used in this work. We have added to the discussion that this is a potential future direction.

4. It is not clear how much the DeepSea priors helped for the MPRAs described in this study. It would be helpful to add a section to the Discussion that provides some guidance to users about when priors are likely to be helpful.

We have added a paragraph with guidance on prior estimation to the Discussion section. We emphasize that external annotations that strongly impact the empirical prior should be treated with caution, as this would imply that the annotations are impacting the posterior as much as the data itself. We prefer for the informative prior to behave in a way that allows rare, strongly functional variants values to show strong estimates and help refine the binary decision in borderline cases (such as with rs11865131). At the same time the prior should continue to shrink the estimates of non-functional variants toward zero.

The figure below (the new S6 Figure) compares the RNA components of the marginal and conditional priors for rs11865131. The depth- and DNA-adjusted RNA counts are shown as tick marks on the x-axis, while the prior means are indicated by vertical dotted lines. Both priors enforce fairly strong shrinkage (prior means are well below the means of the observations), but the change from the marginal to conditional prior makes several important differences. First, the conditional prior shows reduces the shrinkage (i.e. increases the prior mean) for both alleles, showing that similarly annotated variants also showed higher-than-average expression. Second, the prior mean is substantially higher for the reference allele, increasing the prior probability of a negative transcription shift.

Because Figure 4 shows the TS estimates for all variants in the assays, it does not emphasize the subset of variants that are most impacted by the conditional prior.

Figures:

1. According to Figure 3A, the transcription shift for many non-functional variants is higher than the transcription shift for functional variants. It may appear to be this way because the difference between the density of points at the origin and on other parts of the y-axis is not viewable by eye. Modifying this figure to show the difference in density would be helpful. One option is to use smaller points. Alternatively, another part of the figure could be made that zooms in on the y-axis.

We have also added a grey boxplot at x = 0 to figure 3A to clarify the distribution of estimated transcription shifts for truly non-functional variants. We agree with the reviewer that some non-functional variants have estimated TS higher than functional variants, but this is in the nature of stochastic data. Our AUPR and AUC metrics, as shown in Figure 3, indicate that malacoda is performing extremely well compared to the alternatives.

2. It would be helpful to add a panel to Figure 3 that illustrates how the results from malacoda are used to determine which variants are “functional.”

This has been addressed by a component added to Figure 2B. We have also added to the caption for Figure 2B to explain the binary classification procedure. We have reorganized Figure 3 to include only simulation results.

3. It would be helpful to add precision recall curves to Figure 3 to help the reader understand how frequently variants that are called positives are false positives.

We have added precision-recall curves to the new Figure 3C. The results demonstrate that malacoda exhibits superior performance over alternatives by AUPR .

4. It would be helpful to make the x- and y-axes the same scale in Figure 3D or add a line for y = x.

We have added a grey dotted line on y = x in Figure 5A (formerly 3D).

5. It would be helpful to add an explanation of the plots on the diagonals of the plot tables in Figure 4.

We have added this description to the caption for figure 4. We have also removed the potentially confusing marginal distributions on the diagonals in favor of moving the method labels to the diagonal for the sake of simplicity.

Supplement:

1. In the Description of point estimates of variance parameters section, it would be helpful to add a description of how the curve between maximum log-likelihood estimates of means and variances is fit.

There is no universally accepted method of mean-variance curve-fitting used in the RNA-Seq analysis literature, but we have added language that briefly describes the process used by DESeq2 and points the reader to the relevant sections of the DESeq2 publication in Love et al., Genome Biology 2014.

2. In the Simulating variance parameter point estimates section, it would be helpful to add an explanation of why the chosen values are representative of the data from an MPRA or provide a citation of a paper that has such an explanation.

We have added a brief comparison to estimates of the average means and dispersions in the Ulirsch dataset. We have also pointed out that the difference between the simulation parameters we use and the values demonstrated from the Ulirsch dataset would exacerbate the problems flagged in the appendix.

3. In the Simulating variance parameter point estimates section, it would be helpful to add an explanation of why the maximum likelihood estimates are systematically lower than the true value.

This is an inherent property of maximum likelihood estimation of variance-related parameters. Because the maximum likelihood function must estimate the mean in order to estimate the variance, the variance estimates are biased to be too small. We have added a brief description of how we handle this in the prior MLE section of the new S1 Appendix.

4. The NB likelihood surfaces section claims that the simulated draws described on the supplemental website “usually” allow for the true value. It was not clear to me why they do not always allow for the true value; in other words, I know that the true value will not always be drawn, but I am not sure why drawing the true value is not always possible.

Our previous usage of the word “usually” in this sentence was unintentionally misleading. There is non-zero likelihood attributed to all positive values of mean and dispersion. We have adjusted the phrasing at this point to emphasize that the main probability mass of the likelihood surface is relatively near the true value.

5. In the Effect on mean estimation section, the dotted and orange lines on the x-axis were confusing to me. I would recommend removing them.

We have removed the lines on the x-axis.

Reviewer #2: The authors presented a method and an R package called malacoda to model MPRA read count data with negative binomial distribution. The main contribution lies in modeling the mean and dispersion parameters as variables sampled from unknown gamma distribution. The paper is well written in general but there are some missing technical details. Major comments are below.

1. Authors should describe the Bayesian model in much more detail as it is now in the manuscript

a. One page 7, line 144 and 145, authors outlined the two generative NB models for DNA and RNA counts. Then they use Figure 2B to depict the hyperparameter of the gamma distributions that are used to model depath_s\mu_{bc} and \varphi_{DNA} for Counts_DNA. Similarly, another gamma distribution is used eto model depath_s\mu_{bc}\mu{allele} and \varphi_{RNA} variables.

b. Just the generative model itself is not fully described in the main text. What’s distribution of \mu_{bc}? What’s the distribution of \varphi_{RNA}? If some of them are gamma distributed, what are the hyperparameters of these gamma distributions? Fixed or also estimated? Etc etc. No detail described here whatsoever.

We have added the appropriate lines to the probability model presented in the text on lines 174-179 in order to show the distribution of all model parameters. Section 1 of the new S1 Appendix provides a statistical description of the model and a line-by-line walkthrough of the Stan model code used by our method.

c. The details on how the model is inferred is completely omitted. I understand the authors use out of the box optimizer Stan library to do the model fitting part. But that does not mean that there is no need to describe the necessary detail on the Bayesian inference.

The new model fitting appendix includes a walk-through of the model fitting process, including a detailed description of the probability model and the code used to call to fit the model with Stan.

d. In particular, given that \mu_{bc} is unknown and is both DNA and RNA NegBin model, how is the coordinate ascent work in the variational Bayesian and how is it sampled in the no-U-turn Hamiltonian Monte Carlo sampling?

e. If variational Bayesian is used, what’s the proposed distribution?

f. If Hamiltonian Monte Carlo sampling, what’s the leap frog steps? What’s the step size? How the parameters are sampled? Together (not tractable)? Or separately in what order?

We have addressed these three comments in detail in section 2 of the new S1 Appendix. In brief, the models are fit through Stan’s variational interface with the R function rstan::vb() or Stan’s No-U-Turn sampler with rstan::sampling(). The former of these automatically transforms the parameters to the space of real numbers before using a Gaussian variational approximation. The sampler uses an adaptive number of leapfrog at each MCMC iteration and samples from the joint parameter space for each variant individually.

We have also added to the Methods and Results with details about the run time and convergence.

2. In Figure 3D, where authors used their in-house data to show malacoda, what’s the correlation across the 4 methods? It looks like malacoda is not better than the other 3 methods.

The correlation between the luciferase and MPRA estimates of transcription shift in figure 5A (formerly 3D) are as follows:

| **method** | **correlation** |
| --- | --- |
| malacoda | 0.618 |
| mpralm | 0.277 |
| MPRAscore | 0.817 |
| t-test | 0.702 |

We do not feel that these differences are practically significant given that there are only 17 data points. Moreover, the assays shown were not selected to be representative of the most strongly functional variants. Several of the variants turned out non-functional in the luciferase assays, so this subset of variants contaminated with non-functional findings lacks the true positives necessary to assess the relative performance of the analysis methods. We have added content to the results section to clarify this point.

3. For rs11865131, in page 14 line 299, what’s the prediction scores from the other methods? Why is the variant not identified by those methods but by malacoda?

The transcription shift estimates for the other methods for rs11865131 are as follows:

| **method** | **TS estimate** | **p-value** | **95% HDI or FDR Q-value** |
| --- | --- | --- | --- |
| malacoda - marginal prior | -0.169 | NA | (-0.39, 0.05) |
| malacoda - conditional prior | -0.291 | NA | (-0.54, -0.03) |
| MPRAscore | -0.255 | 0.258 | 0.925 |
| mpralm | -0.348 | 0.016 | 0.177 |
| t-test | -0.154 | 0.087 | 0.515 |

The fourth column in the table above (95% HD or FDR Q-value) represents the metrics that would be used in practice to identify functional variants. rs11865131 is not called as functional by the other methods because of its low signal in the dataset. While mpralm and the t-test assign it relatively low p-values, these do not pass the <5% FDR threshold used to identify hits. We have briefly summarised this point in the manuscript on line 370.

4. Page 14 line 306: How many such variants (i.e., rs11865131) exclusively identified by malacoda but not other methods? Can you count the number of ENCODE ChIP-seq peaks that each of the top 100 variants co-localize and compare them with the other 3 methods?

All of the methods evaluated tend to agree on high-signal variants, but each method has some number of variants uniquely identified as functional.

We attempted to perform a comparison of ChIP-seq peak overlaps between methods using the Ulirsch dataset. We did this using the haploR package which interfaces with the HaploReg database in order to annotate the variants with the K562 ChIP-seq peaks they overlapped. HaploReg did not return annotations for around 1500 variants, leaving around 6700 variants. We tried several metrics to concisely quantify the variation in ChIP-seq outcomes and by-method call rate, finally settling on the mean of the log2 number of ChIP-seq bound proteins for variants with at least one bound protein. We compared this quantity between the top 100 hits for each method and all non-hits for each method, yielding the table below. This metric shows some variability in the mean number of peaks among the hits of each method, but we could not find a result that seemed to show a conclusive relationship between ChIP-seq annotations and MPRA outcome in this dataset. While we did not identify a clear relationship, this does not preclude the possibility that future assays designed specifically to examine the transcription shifts of variants with differential ChIP-seq annotations could provide more definitive results.

| **method** | **Hits - Mean log2 number of K562 peaks +/- sd** | **Non-hits - Mean log2 number of K562 peaks +/- sd** |
| --- | --- | --- |
| malacoda - marginal prior | 2.20 +/- 2.25 | 1.57 +/- 1.59 |
| malacoda - conditional prior | 2.72 +/- 2.22 | 1.57 +/- 1.58 |
| t-test | 2.64 +/- 1.97 | 1.54 +/- 1.57 |
| MPRAscore | 3.21 +/- 2.42 | 1.56 +/- 1.58 |
| mpralm | 0.53 +/- 0.92 | 1.59 +/- 1.60 |

5. Authors says their model takes 50,000 MCMC samples, do the model converge in the end? Please show the plot of joint posterior or Hamiltonian energy (since authors is using HMC sampler) as a function of iterations.

We have added detail on the posterior convergence metrics to section 2.4 of S3 Appendix. We have also updated the language (now on lines 510-523) in our manuscript to clarify that the 50,000 sample chains we used were deliberately excessively long in order to obtain very high Monte Carlo precision. This number of samples is usually not needed in order to obtain relatively precise convergence.

6. Regarding more advanced prior, the authors should be aware that there are more advance supervised learning method that is train to predict MPRA signals using sequence features and epigenomic features. See this paper:

a. Li, Y., Shi, A., Tewhey, R., Sabeti, P., Ernst, J., & Kellis, M. (2017). Genome-wide regulatory model from MPRA data predicts functional regions, eQTLs, and GWAS hits. bioRxiv. <http://doi.org/10.1101/110171>

We thank the reviewer for this suggestion. We view this type of predictive modelling as a complementary approach to assay design, rather than a more sophisticated method for estimating informative empirical priors. We would emphasize that the conditional prior estimation presented in our manuscript provides a per-allele gamma distribution selectively biased by prior annotations for each variant. The machine learning paper pointed out by the reviewer only provides numerical scores rather than the distribution that is needed to run a Bayesian analysis.

We checked the correlation between the K562-cell-type-specific scores presented in this paper and found no correlation between the scores presented and the transcription shift values observed in either our primary data or the Ulirsch dataset. It is possible that the machine learning methods presented do not generalize well to novel datasets. However note that this does not preclude the validity of the predictive scores. An assay designed around these scores, that specifically tested a spectrum of high and low scoring variants would likely show a better correlation.
