# Supplementary material for: Bayesian modelling of high-throughput sequencing assays with malacoda
Source: PLoS Comput Biol. 2020 Jul 21;16(7):e1007504. doi: 10.1371/journal.pcbi.1007504 (PMC7394446; doi:10.1371/journal.pcbi.1007504)
Supplement: Attachment — Submitted filename: malacoda_resubmission_letter_2.docx [file pcbi.1007504.s009.docx]

Dear Editor,

We thank the reviewers and editors for their thoughtful comments to our revised submission. We have considered and addressed each of the issues that were raised, and we are pleased to resubmit the revised manuscript addressing these questions as well as our point-by-point responses included below.

We are hopeful that the editor and reviewers will be satisfied with our additional work. Our work represents an important statistical and methodologic contribution to the analysis of high-throughput screen data and is a fully Bayesian framework. We argue that this contribution is a milestone of broad interest and importance to genomics and computational molecular biology.

The key elements of this revision:

1) Reviewer one identified two additional analysis algorithms to be added as comparators to our *malacoda* method. We have added these comparisons to our results; the analyses affirm the performance advantage of malacoda over the alternatives.
2) Reviewer 2 expressed frustration in the formatting of our prior response. We apologize for not presenting our previous response in the format expected by the reviewer. In our response below, after our answer to each question, we have added to the response letter the textual changes made to our manuscript. We hope that this improved formatting facilitates the review and our communication with the reviewer and editors.
3) We further explicate the challenge of orthogonal data comparisons with additional graphical displays and data, and we have added content to the Discussion section accordingly.

The only other minor update of note to our work is the addition of a conditional prior fit to Figure 4A which was done for consistency with 4B.

Below is our point-by-point response to the reviewers comments.

Regards,

Andrew R. Ghazi, Xianguo Kong, Ed S. Chen, Leonard C. Edelstein, and Chad A. Shaw

Reviewer #1

I have one new major comment:

1. I recently became aware of two relevant papers that the authors did night cite: Ashuach et al., Genome Biology, 2019 (https://genomebiology.biomedcentral.com/articles/10.1186/s13059-019-1787-z) and Kalita et al., Bioinformatics, 2018 (https://academic.oup.com/bioinformatics/article/34/5/787/4209990). It would be great if the authors could add an explicit explanation of the key advantages of malacoda over the methods described in these papers or the key differences between the types of problems that malacoda and these methods are capable of solving. If these methods are solving the same problem as malacoda, it would be great if the authors could add comparisons to these methods.

We thank the reviewer for this comment, and we have added these methods to our comparisons, including updates to Figures 3 and 4 and S3 Appendix. The results demonstrate that malacoda is superior to these and the other alternative methods in terms of the various metrics used in S3 Appendix.

I have a few new minor comments:

Introduction:

1. In line 126, I would replace “wet bench” with “luciferase.”

We have made this change.

Appendices:

1. The additional details in Appendix S1 are extremely informative for understanding exactly how malacoda works and how to run different parts of the method. malacoda seems to require the selection of multiple settings, including the number of warm-up samples, the number of samples per chain, and the total number of samples for MCMC. It would be great if the authors could add a description of how the recommended settings were selected.

We thank the reviewer for their positive comment. We would first like to point out that the settings mentioned by the reviewer are preset to default values; these defaults strike a balance between fast exploratory analysis and precision of the posterior approximations. As can be seen in the traceplots of section 2.4.2 of S1 Appendix (now shown at the end of this letter on page 13-14 below), the sampler converges quite quickly, so the choice of defaulting to 2000 samples was motivated not by convergence, but by the desire to obtain precise edges for the 95% posterior intervals.

We have updated the main text in the following sentences in the Computational methods and software section in order to explicitly point out and motivate these settings: "By default, each variant is first checked with a variational first pass. Then, if the variant passes the posterior interval check, MCMC is performed with 4 chains using 200 warmup and 500 post-warmup samples per chain for a total of 2000 posterior samples. These default settings can refine the TS posterior interval with satisfactory precision within a short run time.”

We have also added the bracketed phrase below into the revised manuscript to reiterate this point:

“The computational performance was first evaluated using the default settings of the malacoda package [which are set to strike a balance between speed and precision for exploratory analysis].”

When using the default settings, the software package also prints a recommendation stating that longer MCMC chains should be used for highly precise analyses.

2. Appendix S2 provides a helpful explanation of why having a prior on φ in the negative binomial distribution has the potential be beneficial, but it was clear what if any part of example 6 was illustrating malacoda’s prior on φ. It would be great if this could be clarified.

We thank the reviewer for their remarks. The intention of section 6 in S2 Appendix is to demonstrate the importance of probabilistically modeling the uncertainty on ϕ rather than using a point estimate. This is a general point about estimating parameters of negative binomial distributions that has potentially been overlooked or misunderstood in molecular genomics and that is not specific to malacoda. In this way, the malacoda approach is new to NGS screen data and it represents a significant advance.

Section 6 in S2 Appendix presents the simple case of inferring a single negative binomial distribution with two parameters, μ and ϕ. A larger analysis of realistically simulated MPRA variant data involves many interrelated parameters which would obscure section 6’s intended message about the impact of probabilistically modelling ϕ on the inference on μ. Furthermore, we implemented more realistic MPRA simulations in sections 2-4 in S3 Appendix and showed the practical outcomes on MPRA inference in the main manuscript in Figure 3 and the S3 Appendix. The superior performance of malacoda in the simulated MPRA is partially due to the fact that none of the alternative methods utilize probabilistic inference on ϕ.

We have added the following paragraph to the end of S2 Appendix section 6 in order to guide the reader through these points:

“The model used by malacoda is more complicated than estimating a single negative binomial distribution as shown in this example. The example shown is intended to communicate clearly the value of quantifying the uncertainty on ϕ. In an MPRA context, ϕ is unknown. By modelling ϕ as an unknown parameter rather than utilizing a point estimate, we obtain more accurate inference on not only for ϕ but also for μ and related quantities including the transcription shift. The simulation results presented in Figure 3 of the manuscript drive home the performance benefits of this approach.”

Reviewer #2

It’d have been great if the authors were to describe what they have done to address my comments as opposed to just pointing me to a somewhere in the document. Especially, the changes are not highlighted relative to the original draft.

- For example, in response to my comment 1 on the distributions of the model, authors pointed me to line 174-179, where there is no distribution specified there but rather lines 151-156 have some added distributions. Authors point me to “Section 1 of the new S1 Appendix” on the rest of my comments expecting me to find the answers myself.

We apologize for the inconvenience and difficulty. We now include more detail in the responses to facilitate the review. The incorrect line numbers was an unfortunate mistake; we did not realize at the time that Microsoft Word counted comment content in the line numbering thus making manuscript versions with and without with tracked changes show different line numbering. This regrettably caused some confusion, but the reviewer correctly inferred that we intended to refer to lines 151-156 in the clean version.

We thoroughly agreed with the reviewer’s comment in the first round of reviews that the model should have been described more clearly in our initial submission. Therefore we provide a detailed walkthrough of the model, which is section 1 of S1 Appendix. This appendix supplies the statistical model definitions, the listing of parameters and also step-by-step code blocks that show the implementation of the model.

Below is the introduction of section 1 of S1 Appendix with minor reordering for clarity in the context of this letter. This serves as the most direct response to the reviewer’s request for a more detailed model description. It also includes the Stan model code itself -- which defines the model -- with comments added for clarity. The subsequent sections in S1 Appendix go into further detail breaking down how each code block works and how the model is fit and evaluated in practice.

“In statistical notation, the malacoda model is as follows:

with *s* varying from 1 to the total number of sequencing samples of each type and *bc* varying from 1 to the total number of barcodes. μ_RNA_ and ϕ_RNA_ each have two elements for the ref and alt alleles. The α and β parameters are estimated empirically, while the depth_s_ factors are directly proportional to the number of barcode reads in each sequencing sample.”

The statistical model is implemented in Stan, and section 1 of S1 Appendix also presents the Stan model explicitly:

“This section overviews the probability model underlying malacoda. The code block below gives a complete copy of bc_mpra_model.stan, the Stan file used in the package to analyze the MPRA data for a single variant given a previously estimated empirical prior (the prior estimation process is overviewed in the “Model fitting walkthrough” section below). Stan model files are broken into “blocks”:

- data describes the numeric data to be passed to the model
- parameters describes the model parameters to be evaluated when fitting the model
- model describes the probabilistic relationships between the data and parameters
- generated quantities computes downstream quantities of interest that are directly computable from a given set of parameter values

The following subsections give a detailed description and walkthrough of each block in the malacoda Stan model, which here is presented in full:

```
data {
  int<lower=0> n_rna_samples;
  int<lower=0> n_dna_samples;
  int<lower=1> n_ref; // number of reference barcodes
  int<lower=1> n_alt; // number of alternate barcodes
  int<lower=0> ref_dna_counts[n_ref, n_dna_samples];
  int<lower=0> alt_dna_counts[n_alt, n_dna_samples];
  int<lower=0> ref_rna_counts[n_ref, n_rna_samples];
  int<lower=0> alt_rna_counts[n_alt, n_rna_samples];

  real<lower=0> rna_depths[n_rna_samples];
  real<lower=0> dna_depths[n_dna_samples];

  real<lower=0> dna_m_a;
  real<lower=0> dna_m_b;

  real<lower=0> dna_p_a;
  real<lower=0> dna_p_b;

  real<lower=0> rna_m_a[2]; // 1 = ref, 2 = alt
  real<lower=0> rna_m_b[2];
  real<lower=0> rna_p_a[2];
  real<lower=0> rna_p_b[2];
}
parameters {
  vector<lower=0>[n_ref] dna_m_ref;
  vector<lower=0>[n_alt] dna_m_alt;
  real<lower=0> dna_p;

  vector<lower=0>[2] rna_m; // rna mean
  vector<lower=0>[2] rna_p; // rna phi aka size
}
model {
  // DNA priors
  for (bc in 1:n_ref) {
    dna_m_ref[bc] ~ gamma(dna_m_a, dna_m_b);
  }

  for (bc in 1:n_alt) {
    dna_m_alt[bc] ~ gamma(dna_m_a, dna_m_b);
  }

  dna_p ~ gamma(dna_p_a, dna_p_b);

  // DNA likelihood
  for (s in 1:n_dna_samples) {
    for (bc in 1:n_ref) {
      ref_dna_counts[bc,s] ~ neg_binomial_2(dna_m_ref[bc] * dna_depths[s], dna_p);
    }

    for (bc in 1:n_alt) {
      alt_dna_counts[bc,s] ~ neg_binomial_2(dna_m_alt[bc] * dna_depths[s], dna_p);
    }
  }

  // RNA priors
  for (allele in 1:2) {
    rna_m[allele] ~ gamma(rna_m_a[allele], rna_m_b[allele]);
    rna_p[allele] ~ gamma(rna_p_a[allele], rna_p_b[allele]);
  }

  // RNA likelihood
  for (s in 1:n_rna_samples) {
    for (bc in 1:n_ref) {
      ref_rna_counts[bc, s] ~ neg_binomial_2(rna_m[1] * rna_depths[s] * dna_m_ref[bc], rna_p[1]);
  }

    for (bc in 1:n_alt) {
       alt_rna_counts[bc, s] ~ neg_binomial_2(rna_m[2] * rna_depths[s] * dna_m_alt[bc], rna_p[2]);
    }
  }
}
generated quantities {
  real ref_act;
  real alt_act;
  real transcription_shift;
  ref_act = log(rna_m[1]);
  alt_act = log(rna_m[2]);
  transcription_shift = alt_act - ref_act;
}
```

”

- For the variational Bayesian comments, the authors said “Stan’s variational interface with the R function rstan::vb(), …, former of these automatically transforms the parameters to the space of real numbers before using a Gaussian variational approximation.” How does the Gaussian approximation work on Negative Binomial? It may seems that my comments are too harsh. However, because this is a technical methodology paper, to me, the main contribution of this paper is on this detailed modeling.

The variational Bayes method dramatically speeds up the posterior evaluation in malacoda, so that the more precise MCMC calculations are focused on situations that are relevant as potentially positive findings. It’s also worth pointing out that the variational first pass can easily be switched off by users that are concerned by the variational approximation.

In the case of a negative binomial distribution parameterized by the mean and dispersion, both parameters are constrained to be positive real numbers. The variational interface will therefore use a log transform in order to transform these parameters before fitting the Gaussian approximating distribution on this transformed scale. rstan::vb automatically applies this transformation separately to each parameter being estimated by the model. It is important to emphasize that the Gaussian approximation applies to the parameters -- the data are still modelled as discrete counts from a negative binomial distribution.

We have added the following sentence to section 2.2 of S1 Appendix to clarify this point:

“Because each of the mean and dispersion parameters estimated in a malacoda fit are all constrained to be positive real numbers, rstan::vb() will first apply a log transformation to each parameter (thus transforming their support to the entire real axis) before fitting the Gaussian variational distribution.”

It’s difficult to visualize how this works for the entirety of a malacoda model fit because of the model’s high dimensionality (given that there is a mean parameter being estimated for each barcode). However as a demonstrative example we have plotted below the log-transformed posterior marginal distributions for four parameters of interest for variant 1_155271258_1-3 from the Ulirsch dataset (the same example variant used throughout S1 Appendix). These histograms represent 50000 draws from the true posterior for each of these parameters. While there is slight non-normality visible in each of these, it is clear that a Gaussian approximating distribution would work quite well.

- If the authors go through this round of the review, I would like to see in their response the model details instead of pointing me to somewhere else in the documents.

We have provided these details above in response to reviewer #2’s first comment. The content added to S1 Appendix is now also included in this response letter.

- For my comment 2, the correlation for malacoda is worse than the other two competitors (MPRAscore and even simple t-test). Authors said that this is not practically significant. But I thought the reason the authors show this scatter plot is to demonstrate that their method is better than other methods. Authors also said that “the assays shown were not selected to be representative of the most strongly functional variants”. But why not select the “representative of the most strongly functional variants” to test?

We thank the reviewer for their comment. The variants we chose for the luciferase assays were originally selected because they had discordant results between the marginal and conditional malacoda fits. We initially intended to demonstrate the impact of the conditional prior with these luciferase assays. In hindsight this was a mistake, because this set of variants included several borderline variants with true effects seemingly very close to zero. These “true negatives” essentially only add noise to the inter-method comparison. Because the variants tested were selected in this way -- where they are contaminated with likely true negatives that are in fact non-functional -- the differences between methods in correlation with the luciferase results are neither statistically nor practically significant. Given the small number of noisy luciferase assays and inclusion of nonfunctional variants, one should not ascribe much meaning to the fact that some methods correlate with the noise more highly than malacoda. Given the totality of these considerations and the fact that these luciferase assays are both oblique to the main message of our paper and cause confusion, we have changed what was previously Figure 5A to supplementary S7 Figure.

We would emphasize that the malacoda method did identify a novel, luciferase-validated functional variant missed by the other approaches. This is a biologically relevant intronic variant rs11865131 that was experimentally validated.

- On my comment 4, authors show a table comparing the consistency between top 100 variant predicted by each method with ChIP-seq peaks. It seems MPRAscore once again does better than malacoda. Even t-test has better lower false positive rate and comparable true positive rate. This does not seem to support malacoda as the method of choice.

We appreciate this comment from the reviewer and recognize its importance. The concept of utilizing an independent data set to differentiate MPRA analyses is highly desirable. Unfortunately, as we show, there are considerable obstacles, and at present this analysis is not feasible. The key insight we wish to convey is a) the compounding error inherent in the MPRA and annotation data b) the misalignment between MPRA experiments -- which are concerned with function in a local genomic context -- and global binding or accessibility assays that may identify sites that are truly functional but are not identifiable as functional in a MPRA study. These MPRA false negatives can occur because they require a larger span of genomic context to function or for other reasons that cannot be reproduced in the MPRA context. Together, these obstacles present formidable challenges to the desired analysis.

To address the reviewer comment, we re-examine the differences presented in the original table which is reproduced below. This table compares the log2 number of ChIP-Seq peaks that overlap MPRA hits and nonhits by each method in K562 transcription factor binding data. While there is some variability in the mean value between methods, the differences are not significant relative to the standard deviations shown in the table. Given the extent to which these intervals overlap, it does not show that MPRAscore or indeed any method does any better than any other, nor does it speak to true/false positive rates. No method has a significantly higher ChIP-Seq signal than the others.

As stated in our previous response, it is difficult to find a clear systematic relationship between ChIP-Seq annotations and MPRA outcomes in general, and there is not enough precision in the datasets to discriminate the MPRA analysis methods. We would propose that this is due to the number of intermediate steps separating these two measures of gene regulation, each of which introduces a degree of noise. There is error in the MPRA detection of functional variants, there is error in ChIP-Seq peak calling, and a large number of MPRA variants were not annotated by peaks. Most importantly, transcription factor binding does not guarantee MPRA functionality, so these two types of data are fundamentally misaligned and will have only an imperfect correlation in the ideal case.

| **method** | **Hits - Mean log2 number of K562 peaks +/- sd** | **Non-hits - Mean log2 number of K562 peaks +/- sd** |
| --- | --- | --- |
| malacoda - marginal prior | 2.20 +/- 2.25 | 1.57 +/- 1.59 |
| malacoda - conditional prior | 2.72 +/- 2.22 | 1.57 +/- 1.58 |
| t-test | 2.64 +/- 1.97 | 1.54 +/- 1.57 |
| MPRAscore | 3.21 +/- 2.42 | 1.56 +/- 1.58 |
| mpralm | 0.53 +/- 0.92 | 1.59 +/- 1.60 |

In a further attempt to respond to the reviewer’s comments and insights, we tried repeating this analysis using DeepSea DNase hypersensitivity annotations in place of the ChIP-Seq annotations from HaploReg. We hoped this alternative would be more powerful to detect differences because it provides a real valued score to nearly all variants in the Ulirsch MPRA, avoiding the steps of dropping un-annotated or zero-count variants in the HaploReg analysis. Unfortunately, this result is equally inconclusive to the K562 bound protein result, and serves to reinforce the difficulty of this analytic approach.

| **method** | **Hits - mean absolute DeepSea DH score (+/- SD)** | **Non-hits - mean absolute DeepSea DH score (+/- SD)** |
| --- | --- | --- |
| malacoda - conditional | 2.15 +/- 2.07 | 1.42 +/- 1.22 |
| malacoda - marginal | 2.00 +/- 1.98 | 1.43 +/- 1.23 |
| mpralm | 2.13 +/- 2.01 | 1.43 +/- 1.24 |
| MPRAnalyze | 1.80 +/- 1.86 | 1.44 +/- 1.25 |
| MPRAscore | 2.27 +/- 2.36 | 1.43 +/- 1.23 |
| QuASAR-MPRA | 1.97 +/- 2.08 | 1.45 +/- 1.26 |
| t-test | 2.68 +/- 2.59 | 1.43 +/- 1.23 |

The red dots and bars represent the mean and standard deviation values presented in the table above. In this case, there is a small, consistent difference between hits and non-hits by this metric. Nonetheless, it is clear that there is insufficient data to distinguish any effect of analysis method on this outcome. To quantify this analysis and to reinforce our visual conclusion, we performed a two-way ANOVA in R. The input data frame and results are shown below, with the small F-statistic for the analysis method term shown in bold. This analysis fails to reject the null hypothesis that the mean DeepSea DNase annotations are different by method:

```
> aov_input
# A tibble: 58,205 x 4
   variant_id     DeepSeaDNaseScore method     is_hit
   <chr>                      <dbl> <fct>      <lgl>
 1 1_3684954_2-3             0.995  MPRAnalyze FALSE
 2 1_3687739_2-3             2.86   MPRAnalyze FALSE
 3 1_3691240_2-3             2.34   MPRAnalyze FALSE
 4 1_3691528_2-3             3.52   MPRAnalyze FALSE
 5 1_3691727_2-3             0.0645 MPRAnalyze FALSE
 6 1_3693909_2-3             1.27   MPRAnalyze FALSE
 7 1_3694796_2-3             0.254  MPRAnalyze FALSE
 8 1_3697291_2-3             0.230  MPRAnalyze FALSE
 9 1_39992878_2-3            2.09   MPRAnalyze FALSE
10 1_39998523_2-3            1.06   MPRAnalyze FALSE
# … with 58,195 more rows

> levels(aov_input$method)
[1] "conditional" "marginal"    "mpralm"      "MPRAnalyze"  "MPRAscore"   "QuASAR-MPRA" "t-test"

> summary(aov(DeepSeaDNaseScore ~ is_hit + method, data = aov_input))
               Df Sum Sq Mean Sq F value Pr(>F)
is_hit          1    825   825.2 507.656 <2e-16 ***
method          6      4     0.6   0.374  0.896
Residuals   58197  94603     1.6
---
Signif. codes:  0 ‘***’ 0.001 ‘**’ 0.01 ‘*’ 0.05 ‘.’ 0.1 ‘ ’ 1
```

This ANOVA result does not change with any normalizing transformations we tried on the data.

In summary, although it is desirable to use an orthogonal data set to comparatively evaluate the MPRA methods, such an evaluation is not currently possible. We have now added text to the Discussion regarding this point.

“It is desirable to identify an orthogonal gold-standard data set to differentiate the accuracy of MPRA analysis approaches. Such an analysis would define an independent score of functionality for all variants, and then hits and not hits from each MPRA analysis method could be compared for their concordance or correlation with this independent score. We attempted such an analysis using the Ulirsch dataset, ENCODE K562 bound protein levels, and DeepSea DNase hypersensitivity annotations. Unfortunately these analyses were inconclusive, showing no clear difference in annotation scores between analysis methods. There are at least two possible explanations for this difficulty. First, the noise present in both the MPRA and annotation data lowers the power to differentiate the methods. Secondly, there is misalignment between MPRA functionality and are differentially scoring in the annotation data. Both of these factors likely contribute to the negative result. We would postulate that if there were an idealized dataset showing high correspondence in variants that are potentially functionalizable by MPRA and simultaneously differentially scored in the orthogonal annotation data, then this hypothetical dataset could be used to compare the efficacy of the various analysis methods. At present, we know of no data source that would meet these requirements.”

- Comment 5 on 50,000 MCMC samples, instead of pointing to appendix, please add the plots in your response *if* the authors pass through this round.

The code and trace plots we referred to in our first response letter are included below. The first shows the initial 4000 samples of our long MCMC fit (with the warmup samples shaded in grey). The second plot demonstrates how to look at other parameters in the fit, showing the first 100 samples for transcription shift. In this case, with our example variant the sampler finds the reasonable range of parameter values within roughly 20 samples. We also provide below the NUTS energy diagnostic plot, showing the strong alignment between the marginal and transition energy histograms which demonstrates that the sampler efficiently explores the tails of the posterior distribution.

As mentioned in our previous letter, the 50,000 sample analysis run was deliberately excessively long in order to obtain extremely high Monte Carlo precision. We point this out explicitly in the manuscript:

“This initial analysis run of 8251 variants from the Ulirsch dataset took 29 minutes when parallelized across 18 threads on two Intel Xeon X5675 3.07GHz processors. We compared this to a highly precise analysis run on the same dataset with no variational first pass and excessively long 50,000 iteration MCMC chains for all variants, which took fifteen hours with the same number of cores on the same processors”

```
bayesplot::mcmc_trace(post_draws[1:4000,,],
      pars = 'ref_act', n_warmup = 2000) +
 labs(x = 'MCMC iteration') +
 scale_color_viridis_d(end = .92)

bayesplot::mcmc_trace(post_draws[1:100,,],
      pars = 'transcription_shift') +
 labs(x = 'MCMC iteration') +
 scale_color_viridis_d(end = .92)
```
