# Supplementary material for: Bayesian modelling of high-throughput sequencing assays with malacoda
Source: PLoS Comput Biol. 2020 Jul 21;16(7):e1007504. doi: 10.1371/journal.pcbi.1007504 (PMC7394446; doi:10.1371/journal.pcbi.1007504)
Supplement: Attachment — Submitted filename: malacoda_letter_may.docx [file pcbi.1007504.s010.docx]

Dear Editor,

We thank the reviewers for their continued thoughtful consideration of our work. We have addressed each point brought up by the reviewers in their most recent response and hope that this resubmission will address their final concerns. Our point-by-point response is included below this letter.

One request we would like to make at this time is that, should our paper be accepted, we think it would be preferable to include only the HTML versions of the supplements with the online version of the article. We originally included the PDF versions on the chance that they were preferred by the editor or reviewers. However we believe the HTML versions are easier to read, and we have not heard any contrary opinions throughout the review process. We think it would be in the interest of readers to provide only the HTML versions of the supplements alongside the online article.

Regards,

Andrew R. Ghazi, Xianguo Kong, Ed S. Chen, Leonard C. Edelstein, and Chad A. Shaw

**Reviewer #1**

The changes that the authors have made to incorporate my and the other reviewers’ feedback have further improved the manuscript. I think that a major limitation of this manuscript is that there is little evaluation on real data other than in K562, an immortalized cancer cell line that is unlikely to be representative of any cell type in the human body. However, the ideal data for such evaluation does not exist, and the authors stated this limitation very clearly in the Discussion section, so I do not think that this limitation should hold back this manuscript from being published.

We thank the reviewer for this comment. We want to emphasize that our work is methodologic in nature, introducing a Bayesian approach to the analysis of NGS screen data. There is no reason *a priori* to believe that the method applicability would be tied to NGS from a specific cell model. We analyzed the method by simulation comparison to five other methods; we also applied the method to two data sets including a previously published study where our method identified a new finding that is confirmed by experiment. We agree with the reviewer’s point that restricting our work to K562 is an important limitation to our work, and we have added a statement to the discussion regarding this point:

“Finally, our work is limited to MPRA performed in K562 cells, however there is nothing cell-type specific about the malacoda model. Our method can be used in MPRA performed in alternative cell-types so long as they follow the experimental structure outlined in the Methods section.”

I have come up with a few ideas that I have listed below that might help further demonstrate the value of malacoda relative to other methods. I also identified a few parts of the manuscript and github page that I would recommend further clarifying. Since the authors have made all of the code publicly available and provided details in their appendices about how to run malacoda, I think that malacoda could be come a widely used method for using MPRA data to identify putatively functional variants.

I have one new major comment:

1. I think that the paper would benefit tremendously from comparing malacoda to other methods on an additional real MPRA dataset. I recently became aware of a dataset that might be ideal for such a comparison: the dataset in Tewhey et al., Cell, 2016 (https://www.ncbi.nlm.nih.gov/pubmed/27259153). Since there is substantial eQTL data from lymphoblastoid cell lines from datasets like Geuvadis, I think that additionally showing that the putatively functional variants that malacoda detects in this dataset are more likely to overlap with eQTLs (or SNPs in linkage disequilibrium with eQTLs) than those detected by other methods would make the value of malacoda relative to other methods more apparent.

We appreciate the reviewer’s comment that our malacoda method and software could become a widely used method; we certainly hope so. Our objective is to share our work and our software to enhance the field of NGS screen analysis.

We appreciate the question regarding orthogonal evaluation of results. We point out to the reviewer that the Tewhey experimental design is based on eQTLs taken from the Geuvadis dataset, and therefore a confirmatory analysis using Geuvadis would be circular and not an independent evaluation or confirmation. Furthermore, eQTL association signals in general would not serve as an effective metric to quantify MPRA analysis effectiveness because of the fact that eQTLs with strong associations are inherently contaminated with many non-functional variants due to LD. Distinguishing linked functional and non-functional variants in eQTL datasets is one of the original intended purposes of MPRA.

Secondly, there are a number of experimental considerations that would make incorporating this data into our work non-trivial. The Tewhey dataset is severely undersequenced: roughly 80% of entries in the count matrix are zero, and across samples the data shows much higher variance than either the Ulirsch or our primary dataset. These deficiencies further obscure inter-method comparison beyond the challenges outlined in our previous letter. Additionally, the barcodes in the Tewhey dataset are added to the oligonucleotides randomly by PCR rather than being designed as part of the oligo prior to synthesis. This adds orders of magnitude to the experimental scale, but also introduces potentially non-inert barcodes into the experiment. It also greatly increases the experimental complexity and sequencing demands. We have discussed these points with the lead author of Tewhey et al. and to our understanding this method has fallen out of favor among the broader MPRA experimentalist community. All this is to say that an equitable presentation of this dataset would necessitate a large digression into experimental methods for a paper that is intended to focus on statistical methods. Ultimately we feel that adding another dataset would necessitate greatly expanding the length and scope of manuscript only to re-emphasize the same points made by our existing analysis of simulations, our primary MPRA data, and the previously published Ulirsch dataset.

I have a few new minor comments:

Response to other reviewer:

1. The other reviewer brought up the important point that the putatively functional variants identified by MPRAscore are more likely to overlap ChIP-seq peaks than those identified by malacoda. The authors responded by comparing the DeepSEA DNase annotations between those variants across methods and evaluating the similarity between the annotations with a two-way ANOVA test. I found the description of the two-way ANOVA test a little confusing. My understanding is that the null hypothesis of the two-way ANOVA is that the means of the DeepSEA DNase scores are equal across methods. Thus, my understanding is that not rejecting the null hypothesis, does not imply that the null hypothesis is correct; it implies that the null hypothesis cannot be rejected. If the authors re-did the analysis on LCLs and found more overlap between putatively functional variants identified by malacoda than those identified by other methods, then I would not be as concerned about the ChIP-seq result. Alternatively, the authors could evaluate if the putatively functional variants identified by malacoda tend to be closer to TSS’s or closer to K562 DNase peak summits than those identified by other methods.

We thank the reviewer for the remark. The ANOVA analysis indeed shows only that we fail to reject the null hypothesis that the methods are equivalent in their annotation scores of hits and non-hits. However, we have previously outlined the limitations of this type of inter-method comparison analysis: the annotations are indirect and noisy, and the annotation results identify variants that are functional *in vivo* but are non-functional in an MPRA context. In other words the annotation data superimpose both functional variants detectable by MPRA and actually functional variants that cannot be detected in a MPRA because of construct constraints -- i.e. local genomic context. These same limitations would apply to any annotation metric that could be proposed, including the distances to TSS’s or DNase peak summits suggested by the reviewer. Given our knowledge of these limitations, we interpret the ANOVA result of our previous ChIP-Seq / DeepSea analyses in the following way: there is no difference in these metrics between methods because there are too many sources of error between the process generating the annotation metric and the process generating the MPRA results. Ultimately this is the reason we need to perform MPRA to begin with, because there is no way of knowing what the results will be.

To address this point we have added the following statement to the discussion:

“While this limits our ability to quantify the performance of MPRA analysis methods, it speaks to the value of MPRA themselves. MPRA produce a unique biological signal that cannot be easily measured by other types of experiments or data.”

Introduction:

1. An exciting application of using MPRAs to identify putative functional variants is to help determine which of multiple variants in linkage disequilibrium that have all been associated with a disease are likely to be causal. I think that mentioning this near the beginning of the introduction might encourage more researchers to read the rest of the paper.

We have added the following sentence to the paragraph introducing MPRA in the introduction:

“MPRA can thus be used to identify functional driver variants among sets of statistically significant GWAS variants that are difficult to distinguish in observational studies because of linkage disequilibrium.”

2. In lines 101-102, I think that “transcription binding” should be replaced with “transcription factor binding.”

We have made this change.

3. In line 106, I would replace “has been unclear” with “has not been developed” (if that is accurate).

We have made this change.

Methods:

1. In line 181, I would replace “learn” with “evaluate.”

We have made this change.

Results:

1. The way that the p-values were computed was clear from the Appendix, but the way that the p-values were used to compute AUC and AUPR was not immediately intuitive to me. It would be helpful if this were described in more detail.

The ROC, AUC values, and PR curves were all computed using the pROC R package by giving the pROC::roc() function the true functional/non-functional labels and the associated p-values for each simulated variant. This package does not provide a method for computing the AUPR, so we calculated those values manually from the PR curve coordinates using the trapezoidal rule.

Discussion:

1. The Discussion section describes some extensions to malacoda that are available in the software package but are not described in detail or analyzed in this paper. I would recommend removing these and writing another paper about them that describes them in detail. Other researchers might be reluctant to use them if they do not have access to a clear explanation of how they work.

We have left the mention of these methods in the paper, but have added the following sentence to the beginning of the paragraph introducing them to further emphasize that these are “future directions” that help to emphasize the long term potential of our work.

“The statistical method and validation work presented in this article present many future directions in the statistical analysis of high-throughput sequencing assays.”

Figures:

1. The color-coding at the bottom of Figure 1 might be a little confusing to some readers because the red RNA could be interpreted as meaning that all of the RNA is coming from the first variant. I might instead make the RNA corresponding colors to the variant that produced it or make it similar to the brown color of the DNA that will get transcribed in the middle of the figure.

We have reworked several aspects of Figure 1. We have changed the color of the RNA to green instead of brown to emphasize that it is a different type of nucleic acid than the brown ORF. We have also added a column of miniaturized versions of the genomic context to the bottom section in order to indicate that the barcodes correspond to the same 8 oligonucleotides shown throughout the figure. We have also added miniaturized plasmids to the section depicting the plasmid library in order to heighten the correspondence between sections.

We have also expanded the caption for Figure 1 in response to these changes:

“**Fig 1. Diagram of MPRA.** MPRA simultaneously assess the transcription shift of thousands of variants. The diagram shows eight oligonucleotides for two variants (red and blue X’s) falling within different regions of genomic context (light red and light blue bars) with two barcodes for each allele of each variant. In practice the complexity and size of the oligonucleotide library is limited only by cost. A typical MPRA has tens to hundreds of thousands of oligonucleotides to assay thousands of variants. The oligonucleotides are cloned into a plasmid library in front of an inert ORF (brown). DNA sequencing of the plasmid library is used to count the input representation of each barcode, then RNA sequencing of the mRNA (green) is used to count the output RNA version of each barcode.”

Appendices:

1. The code is generally easy to follow; removing the commented-out code would make some parts even easier to follow.

We have gone through the code blocks in the appendices and removed superfluous commented-out code.

github page:

1. I think that a more detailed description of the inputs would make malacoda easier to use. Specifically, a description of the exact format of mpra_data would be helpful.

We have added a small example to the Example section of the Github README and pointed to the part of the documentation containing the specific formatting requirements of the input.

2. I had trouble finding the help documentation on the github. It would be great if a link to it could be added in a prominent location.

We have added a Help section to the README file on the Github page listing ways to access the documentation and walkthrough vignette.

3. There seem to be some R scripts in the github that are not explained anywhere on the github. It would be great if a brief description of each R script could be added.

We have added a README.R file in the R/ directory of the package that overviews the purpose of each .R file.

**Reviewer #2:**

Authors have addressed the technical concern and clarity comments that I have. Please add legends to Figure 3B and 3C.

We have added inset color legends to 3B and 3C

Also, Figure 3D median spear at zero is confusing. Authors should just add error bar to the bars displayed on the first three panels for median AUC, AUPR, non-zero correlation.

The spread at zero metric in Figure 3D does not represent the error in the other panels. The spread at zero metric quantifies how well each method shrinks the estimates of truly non-functional variants in the simulated assays. This metric is complementary to the non-zero correlation metric: the non-zero correlation measures how well we estimate the effect of functional variants, while the spread at zero metric quantifies how well we shrink the estimates of truly non-functional variants.

We have adjusted the language in the manuscript to make this point more explicit:

“The last metric, “spread at zero”, particularly emphasizes the regularization effect, showing that while malacoda tends to produce the most accurate effects for functional variants, it can simultaneously provide the smallest estimates for truly non-functional variants.”
